# Insights into the environmental reservoir of pathogenic *Vibrio parahaemolyticus* using comparative genomics

**DOI:** 10.3389/fmicb.2015.00204

**Published:** 2015-03-24

**Authors:** Tracy H. Hazen, Patricia C. Lafon, Nancy M. Garrett, Tiffany M. Lowe, Daniel J. Silberger, Lori A. Rowe, Michael Frace, Michele B. Parsons, Cheryl A. Bopp, David A. Rasko, Patricia A. Sobecky

**Affiliations:** ^1^School of Biology, Georgia Institute of TechnologyAtlanta, GA, USA; ^2^Division of Foodborne, Waterborne, and Environmental Diseases, National Center for Emerging and Zoonotic Infectious Diseases, Centers for Disease Control and PreventionAtlanta, GA, USA; ^3^Division of Scientific Resources, National Center for Emerging and Zoonotic Infectious Diseases, Centers for Disease Control and PreventionAtlanta, GA, USA; ^4^Institute for Genome Sciences, University of Maryland School of MedicineBaltimore, MD, USA; ^5^Department of Microbiology and Immunology, University of MarylandBaltimore, MD, USA

**Keywords:** genomics, *Vibrio parahaemolyticus*, environment, phylogenomics, O3:K6

## Abstract

*Vibrio parahaemolyticus* is an aquatic halophilic bacterium that occupies estuarine and coastal marine environments, and is a leading cause of seafood-borne food poisoning cases. To investigate the environmental reservoir and potential gene flow that occurs among *V. parahaemolyticus* isolates, the virulence-associated gene content and genome diversity of a collection of 133 *V. parahaemolyticus* isolates were analyzed. Phylogenetic analysis of housekeeping genes, and pulsed-field gel electrophoresis, demonstrated that there is genetic similarity among *V. parahaemolyticus* clinical and environmental isolates. Whole-genome sequencing and comparative analysis of six representative *V. parahaemolyticus* isolates was used to identify genes that are unique to the clinical and environmental isolates examined. Comparative genomics demonstrated an O3:K6 environmental isolate, AF91, which was cultured from sediment collected in Florida in 2006, has significant genomic similarity to the post-1995 O3:K6 isolates. However, AF91 lacks the majority of the virulence-associated genes and genomic islands associated with these highly virulent post-1995 O3:K6 genomes. These findings demonstrate that although they do not contain most of the known virulence-associated regions, some *V. parahaemolyticus* environmental isolates exhibit significant genetic similarity to clinical isolates. This highlights the dynamic nature of the *V. parahaemolyticus* genome allowing them to transition between aquatic and host-pathogen states.

## Introduction

*Vibrio parahaemolyticus* is halophilic aquatic bacterium that is ubiquitous in coastal marine and estuarine environments. The majority of isolates derived from environmental sources, that is water and sediments, are believed to be non-pathogenic (Depaola et al., 1990; Nair et al., 2007); however some *V. parahaemolyticus* isolates are capable of causing human illness, and are primarily associated with food-borne derived gastroenteritis and diarrhea. *V. parahaemolyticus* infection can also be associated with wound infections and sepsis (CDC, 2007, 2008; Tena et al., 2010). In 1996, an increase in diarrheal illness in India associated with *V. parahaemolyticus* infections were attributed to the emergence of a novel genetic variant in 1995 that had the O3:K6 serotype (Okuda et al., 1997). This novel disease-associated O3:K6 clone rapidly disseminated worldwide and is considered to be pandemic (Vuddhakul et al., 2000; Myers et al., 2003; Quilici et al., 2005; Ottaviani et al., 2008). Previously, isolates belonging to the post-1995 O3:K6 clone were identified with the serotypes O1:Kuk, O1:K25, and O4:K68, indicating the O3:K6 clone has undergone serogroup conversion in the years since the original clonal expansion (Nair et al., 2007; Chen et al., 2011). The disease-associated *V. parahaemolyticus* clinical isolates usually carry one or both of the thermostable direct hemolysins (*tdh* and *trh*) (Kaper et al., 1984; Nishibuchi et al., 1992; Nishibuchi and Kaper, 1995; Makino et al., 2003; Nair et al., 2007). In addition to the hemolysins, two type III secretion systems (T3SS) have been demonstrated to secrete effectors that induce cytotoxicity or enterotoxicity (Park et al., 2004; Lynch et al., 2005; Ono et al., 2006; Caburlotto et al., 2010; Broberg et al., 2011; Ham and Orth, 2012; Zhang and Orth, 2013). A previous study revealed a second version of T3SS2, T3SS2β, which was identified in clinical isolates that also possess the *trh* gene (Okada et al., 2009). Although the hemolysins and type III secretion have been identified as a major components of the *V. parahaemolyticus* virulence mechanism (Park et al., 2004; Burdette et al., 2008; Caburlotto et al., 2010; Ham and Orth, 2012; Zhang and Orth, 2013), disease-associated isolates have been identified that do not encode the thermostable direct hemolysins (Yu et al., 2006; Bhoopong et al., 2007; Meador et al., 2007), suggesting there may be additional, as yet, uncharacterized genes contributing to *V. parahaemolyticus* virulence mechanisms.

The genetic diversity of *V. parahaemolyticus* has been investigated using numerous molecular methods, including the identification of known virulence genes (Meador et al., 2007; Noriea et al., 2010; Jones et al., 2012), multi-locus sequence typing (MLST) (Chowdhury et al., 2004; González-Escalona et al., 2008; Gavilan et al., 2013; Turner et al., 2013), phylogenetic analysis of housekeeping genes (Thompson et al., 2005), microarray (Han et al., 2008), and pulsed-field gel electrophoresis (PFGE) (Parsons et al., 2007; Ludeke et al., 2014). MLST was used to identify two new clonal complexes in addition to a clonal complex of the post-1995 O3:K6 isolates (González-Escalona et al., 2008). The second clonal complex consisted of O4:K12 and O12:K12 isolates from the Pacific coast of the United States, and the third clonal complex was comprised primarily of isolates from oysters in the Gulf of Mexico (González-Escalona et al., 2008).

Genome sequencing and comparative analysis of the post-1995 *V. parahaemolyticus* O3:K6 isolate RIMD2210633 (Makino et al., 2003) revealed seven genomic islands, including four that are characteristic of post-1995 O3:K6 isolates (Hurley et al., 2006; Boyd et al., 2008; Chen et al., 2011). Genomic subtraction demonstrated that an 80-kb pathogenicity island (Vp-PAI) encoding T3SS2 was associated with the post-1995 O3:K6 pandemic isolates (Okura et al., 2005). The sequencing of additional *V. parahaemolyticus* genomes has confirmed that the emergence of the post-1995 O3:K6 pandemic isolates coincided with the acquisition of genomic islands as these regions were mostly absent from the genomes of pre-1995 O3:K6 isolates (Makino et al., 2003; Boyd et al., 2008; Chen et al., 2011).

The purpose of this study was to investigate the genetic diversity of *V. parahaemolyticus* isolates from human clinical (stool, blood, wound specimens, or unknown sample types) or environmental (sediment, water, oysters) sources using multiple molecular methods including a PCR assay of known *V. parahaemolyticus* virulence-associated genes, phylogenetic analysis of housekeeping genes, PFGE, and whole-genome sequencing. Investigation of the genomic diversity of two clinical isolates and four environmental isolates by whole-genome sequencing and comparative analysis identified genes that are shared or exclusive to the clinical or environmental isolate genomes sequenced. These methods highlight the genetic similarity among clinical and environmental isolates, and the different combinations of virulence-associated genes demonstrate the dynamic nature of the *V. parahaemolyticus* genome.

## Materials and methods

### Bacterial isolates and media

*V. parahaemolyticus* clinical isolates included in this study were provided by the Centers for Disease Control and Prevention (Atlanta, GA). The *V. parahaemolyticus* environmental isolates were cultured from sediment, water, and oysters of Skidaway Island, GA, and Apalachicola Bay, FL in September 2006, and Skidaway Island, GA in September 2007 (Hazen et al., 2009). Additional environmental isolates were obtained from the rhizosphere sediment of a salt marsh in North Inlet, NC (Bagwell et al., 1998). The environmental isolates were cultured by plating environmental samples on thiosulfate citrate bile salts sucrose (TCBS) agar (Difco) and incubating them overnight at 30°C. Water samples were directly plated onto TCBS, while sediment, and oysters were homogenized with sterile water then plated onto TCBS agar. Presumptive *V. parahaemolyticus* colonies that were green on TCBS were confirmed by PCR by screening for the thermolabile hemolysin (*tl*) as previously developed (Bej et al., 1999), which is characteristic of *V. parahaemolyticus* (Meador et al., 2007). The culture collection strain, ATCC 17802, was used as a reference isolate for molecular characterizations of *V. parahaemolyticus*.

### Serotyping

Serotypes were determined using *V. parahaemolyticus* Seiken typing antisera (Denka Seiken, Tokyo, Japan).

### PCR assay of virulence-associated genes

The known *V. parahaemolyticus* virulence-associated genes were detected by PCR assay for all *V. parahaemolyticus* clinical and environmental isolates examined in this study using primers listed in Supplemental Table 5. All isolates that were positive for *tl* as described above were then PCR screened for previously-characterized virulence-associated genes. The thermostable direct hemolysins *tdh* and *trh* were detected as described (Bej et al., 1999; Meador et al., 2007). In addition, the ORF8 gene of the pandemic phage f237 was detected using primers that were previously developed (Myers et al., 2003). The presence of the T3SS1 and T3SS2 were determined by PCR assay for two effectors and one gene involved in translocation from each T3SS. The T3SS1 effectors *vp1680* and *vp1686* and the structural gene *vp1670* was identified by PCR assay using previously developed (Vora et al., 2005; Meador et al., 2007) primers, and additional primers made in this study that are listed in Supplemental Table 5. The presence of T3SS2α and T3SS2β was determined by PCR assay for the effectors *vpa1346* and *vpa1362*, and the export protein-encoding gene *vpa1354* using primers listed in Supplemental Table 5.

### Phylogenetic analysis of housekeeping genes

The genetic similarity was investigated for 116 *V. parahaemolyticus* clinical and environmental isolates examined in this study by phylogenetic analysis of a concatenation of four housekeeping genes (*recA, gyrB, pyrC, dtdS*) using previously developed primers (González-Escalona et al., 2008). The genes were PCR amplified using NEB Phusion high-fidelity polymerase (NEB; Ipswich, MA) and purified by separation on a 0.7% Seakem LE agarose gel (Lonza; Allendale, NJ). The target amplicon was excised from the gel and the DNA was recovered using the Sigma GenElute gel extraction kit (Sigma Aldrich; St. Louis MO). Sequencing was performed with M13 primers at the Georgia Tech Genome Center on an ABI 3130 Genetic Analyzer (Applied Biosystems) using BigDye Terminator chemistry (Applied Biosystems). Sequences were assembled in BioEdit (v. 7.0.4.1) (Hall, 1999) and aligned using MEGA5 (Tamura et al., 2011), and all sequences for a particular gene were trimmed to the same length. The partial sequences of each housekeeping gene analyzed were concatenated in the same order for each *V. parahaemolyticus* isolate, generating a single representative sequence. A maximum-likelihood phylogeny with 100 bootstrap replicates was generated using RAxML v7.2.8 (Stamatakis, 2006) and visualized using FigTree v1.3.1 (http://tree.bio.ed.ac.uk/software/figtree/).

The genetic similarity was further investigated for 52 of the clinical and environmental isolates by analysis of three additional genes (*dnaE, tnaA, pntA*) for a total of seven genes. These three additional genes were PCR amplified and described above using previously developed primers (González-Escalona et al., 2008). Phylogenetic analysis of all seven (*recA, gyrB, dnaE, pyrC, dtdS, tnaA, pntA*) of the conserved genes for this subset of clinical and environmental isolates was performed as described above.

### PFGE

Pulsed-field gel electrophoresis of 44 *V. parahaemolyticus* clinical and environmental isolates was performed according to the *V. parahaemolyticus* PulseNet USA standardized protocol (Parsons et al., 2007). Restriction endonuclease profiles were generated using the enzymes *Sfi*I and *Not*I (Roche, Mannheim, Germany). Restricted plugs were run on a CHEF Mapper™ electrophoresis system (Bio-Rad Laboratories, Hercules, CA). *Salmonella Braenderup* H9812 restricted with 50 U of *Xba*I (Roche, Mannheim, Germany) was used as a control strain for gel normalization. PFGE patterns were analyzed with BioNumerics v. 5.1 (Applied-Maths, Kortrijk, Belgium) and dendrograms were generated using the Dice coefficient and unweighted pair group method with arithmetic averages (UPGMA) with a band position tolerance and optimization of 1.5% for cluster analysis.

### Genome sequencing and assembly

Following the molecular characterization of the *V. parahaemolyticus* clinical and environmental isolates, we generated high-quality draft genome sequences of two clinical isolates (K1275, K1461) and four environmental isolates (AF91, SG176, J-C2-34, 22702) (**Table 2**). The clinical isolates analyzed have unique combinations of the known virulence-associated genes compared to the epidemic post-1995 O3:K6 isolates (Supplemental Table 1). The environmental isolates analyzed by genome sequencing were obtained from samples of three different states (NC, GA, FL) (Supplemental Table 1). The *V. parahaemolyticus* isolates analyzed by whole-genome sequencing were grown overnight in Luria Bertani (Difco) at 37°C with shaking (225 rpm). Genomic DNA was isolated from the overnight cultures using the Sigma GenElute genomic kit (Sigma Aldrich; St. Louis MO). The genome sequences of *V. parahaemolyticus* isolates K1461, K1275, SG176, J-C2-34, and AF91 were generated using the Roche 454-Titanium sequencing platform at the Centers for Disease Control and Prevention. The 454 reads were assembled into high-quality draft genomes at the Institute for Genome Sciences, using the Mira assembler (Chevreux et al., 1999), and the assemblies were filtered to contain contigs ≥500 bp.

The genome sequence of *V. parahaemolyticus* 22702 was generated using paired-end libraries with 300 bp inserts on the Illumina HiSeq2000 at the Institute for Genome Sciences, Genome Resource Center. The Illumina reads generated for 22702 were assembled into a high-quality draft genome using the Velvet assembly program (Zerbino and Birney, 2008) with kmer values determined using VelvetOptimiser v2.1.4 (http://bioinformatics.net.au/software.velvetoptimiser.shtml), and the assembly was filtered to contain contigs ≥500 bp.

Information regarding the genome assembly size, number of contigs, and the GenBank accession numbers for each of the genomes sequenced in this study are listed in **Table 2**.

### Comparative genomics

Phylogenomic analysis of the *V. parahaemolyticus* genomes sequenced in this study compared to previously sequenced *V. parahaemolyticus* genomes available in the public domain, was performed as previously described (Sahl et al., 2011). The genomes were aligned using Mugsy (Angiuoli and Salzberg, 2011), and the aligned regions were concatenated then used to construct a maximum-likelihood phylogeny with 100 bootstrap values using RAxML v7.2.8 (Stamatakis, 2006), and visualized using FigTree v1.3.1 (http://tree.bio.ed.ac.uk/software/figtree/).

BLAST score ratio (BSR) analysis was performed as previously described (Rasko et al., 2005) and used to identify the presence of virulence-associated genes in each of the genomes analyzed (**Table 2**). Briefly, the predicted amino acid sequences of virulence-associated genes and genomic regions (Hurley et al., 2006; Chen et al., 2011; Salomon et al., 2013) were compared using TBLASTN (Gertz et al., 2006) to all the *V. parahaemolyticus* genomes analyzed in this study. The protein-encoding genes that were considered present with significant similarity had BSR values ≥0.8.

Genetic similarity of chromosomes I and II of the O3:K6 isolate RIMD2210633 to genes in each of the genomes sequenced in this study was determined using BLASTN (Altschul et al., 1990) BSR analysis as previously described (Rasko et al., 2005). A circular display of the BLASTN BSR values was generated using Circos 0.65 (Krzywinski et al., 2009).

Large-scale BSR analysis (Hazen et al., 2013; Sahl et al., 2013, 2014) was used to identify the shared and unique features present in the *six* genomes sequenced in this study compared with eight previously sequenced genomes listed in **Table 2**. Each protein-encoding gene was considered present with high identity (BSR value ≥0.8), present but with sequence divergence (BSR value ≥0.4, ≤0.8), or absent (BSR value <0.4). A representative sequence of each predicted protein-encoding gene is included in Supplemental Data Set 1. The predicted function of protein-encoding genes was identified using the RAST annotation server (Overbeek et al., 2014).

### Plasmid and phage analyses

The number of extrachromosomal elements (plasmids and prophage) was determined for a subset of the isolates using a modified acid phenol extraction method (Kieser, 1984; Sobecky et al., 1997). PCR amplification for sequencing was performed using NEB Phusion high-fidelity polymerase reaction mix with GC buffer and reaction and cycle conditions as recommended by the manufacturer (NEB; Ipswich, MA). Primers used to PCR amplify *rstA* are listed in Supplemental Table 5. The M13 sequence at the 5′ end of each *rstA* primer was used for sequencing. Sequencing was performed as described for the housekeeping genes. The sequences were aligned using MEGA5 (Tamura et al., 2011), and a maximum-likelihood phylogeny using the Kimura 2-parameter model (Kumar et al., 2004) and 1,000 bootstrap replications was constructed using MEGA5 (Tamura et al., 2011). Bootstrap values ≥50 are shown.

### Nucleotide sequence accession numbers

All individual gene sequences generated in this study are deposited in GenBank under the accession numbers FJ847518-FJ847829. The genome sequences are deposited in GenBank under the accession numbers JMMO00000000, JMMP00000000, JMMQ00000000, JMMR00000000, JMMS00000000, and JMMT00000000.

## Results and discussion

### Identification of virulence-associated genes in a collection of clinical and environmental *V. parahaemolyticus*

As a measure of the virulence potential of *V. parahaemolyticus* clinical and environmental isolates analyzed, we detected common markers of virulence including: the ORF8 gene of the filamentous vibriophage, the hemolysins (*tdh* and *trh*), the type III secretion systems of chromosome I (T3SS1), and chromosome II (T3SS2α and T3SS2β) (Table 1). These genes have been previously identified in association with illness-associated *V. parahaemolyticus*, and the hemolysins and T3SS2 were characterized for their role in pathogenesis (Kaper et al., 1984; Nishibuchi and Kaper, 1995; Nasu et al., 2000; Park et al., 2004; Lynch et al., 2005; Ono et al., 2006; Nair et al., 2007; Broberg et al., 2011; Ham and Orth, 2012; Zhang and Orth, 2013). The ORF8 gene encoded by the filamentous vibriophage f237, which has previously been linked to the post-1995 O3:K6 pandemic clinical isolates (Nasu et al., 2000), was identified in 32% of the clinical isolates, and none of the environmental isolates in this study (Table 1). The ORF8 gene was identified in all post-1995 O3:K6 isolates analyzed, and only 13% of the non-O3:K6 clinical isolates (Table 1; Supplemental Table 1). The T3SS1 genes and *tl* were detected among all *V. parahaemolyticus* isolates, which is consistent with previous reports that these genes are universal among *V. parahaemolyticus* isolates (Vora et al., 2005) (Table 1, Supplemental Table 1). The T3SS2α genes were present in 93% (13/14) of the O3:K6 clinical isolates and 100% (4/4) of the O4:K8 clinical isolates, but only 25% (12/49) of the clinical isolates that had other serotypes (Table 1). T3SS2β was detected in 90% (9/10) of the O4:K12 isolates, and 46% (22/48) of the clinical isolates with other serotypes (Table 1). Included in this study were 10 clinical isolates with the O4:K12 serotype, and all but one (K4358) of these isolates were *tdh*+/*trh*+/T3SS2β+ (Table 1, Supplemental Table 1). Similar virulence-associated gene content (*tdh*+/*trh*+/T3SS2β+) was identified in clinical isolates of six other serotypes (O4:K53, O4:K63, O1:K56, O8:K21, O6:K18, O11:Kuk) (Supplemental Table 1). While T3SS2α was not identified in any of the environmental isolates, including the O3:K6 environmental isolate AF91, T3SS2β was present in two environmental isolates that were *trh*+ (Table 1, Supplemental Table 1). *V. parahaemolyticus* environmental isolates that possess T3SS2 have been demonstrated to adhere to eukaryotic cells and disrupt membrane tight junctions (Caburlotto et al., 2010). This study demonstrated that *V. parahaemolyticus* isolates residing in the environment that possessed some of the known virulence factors also had the potential to cause disease.

**Table 1:** **Identification of *V. parahaemolyticus* virulence-associated genes in a collection of *V. parahaemolyticus* clinical and environmental isolates using PCR assays**.

**Source**	**Serotype**	**No. isolates**	**No. of isolates with virulence genes (%)**					
			**ORF8**	***tdh***	***trh***	**T3SS1**	**T3SS2α**	**T3SS2β**
Clinical	O3:K6	14	14 (100)	13 (93)	0 (0)	14 (100)	13 (93)	0 (0)
	O4:K8	4	0 (0)	4 (100)	0 (0)	4 (100)	4 (100)	0 (0)
	O4:K12	10	1 (10)	10 (100)	9 (90)	10 (100)	0 (0)	9 (90)
	Other serotypes	48	9 (19)	29 (60)	14 (29)	48 (100)	12 (25)	22 (46)
Total clinical	All serotypes	76	25 (32)	57 (74)	23 (30)	77 (100)	29 (38)	31 (40)
Environmental	O3:K6	1	0 (0)	0 (0)	0 (0)	1 (100)	0 (0)	0 (0)
	Other serotypes	56	0 (0)	0 (0)	2 (3)	56 (98)	0 (0)	2 (3)
Total environmental	All serotypes	57	0 (0)	0 (0)	2 (3)	57 (100)	0 (0)	2 (3)

There were also clinical isolates that had an atypical combination of virulence genes, or were missing most of the known virulence-associated genes. Several clinical isolates contained only *tdh* (K0071, F5828, K4358, K4279), or both *tdh* and *trh* genes (K5067), but lacked detectable T3SS2 genes (Table 2). In addition, the clinical isolates K4763, K3528, and K4305 contained *tdh* and T3SS2β genes, which is unusual as the *tdh* gene is typically associated with T3SS2α (Sugiyama et al., 2008). It is possible these isolates may have contained both *tdh* and *trh*, similar to O4:K12, and they may have lost *trh* during infection or during laboratory passage. None of the isolates analyzed contained the *trh* gene and also the T3SS2α genes. Three clinical isolates (F8950, F8937, K4377) contained T3SS2α but lacked *tdh* and *trh*, and five clinical isolates (K0456, K4237, K4638, K5323G, K5330) contained T3SS2β but lacked *tdh* and *trh* (Supplemental Table 1). The presence of T3SS2 genes and the absence of hemolysins in clinical isolates has been previously described (Meador et al., 2007). There were 12 clinical isolates (K1275, K0851, K0850, F9974, F6658, F8132, F7979, F6179, K4434, F8190, K4981, K1000) that did not encode *tdh, trh*, or the T3SS2 genes (Supplemental Table 1). These isolates were obtained from blood (K1275), wound infections (F8132, K4434), or unknown clinical sample types (F6658, F6179, F8190, K4981, K1000). Although the *V. parahaemolyticus* clinical isolates that lacked the hemolysin and/or T3SS2 genes were obtained from clinical specimens, they may have been co-occurring in the host with other *V. parahaemolyticus* isolates that did encode the hemolysins or T3SS2 genes and were the primary cause of illness. A previous study demonstrated that multiple *V. parahaemolyticus* isolates were present in disease-associated samples; however, some of these isolates lacked the hemolysin genes (Bhoopong et al., 2007). Another possible explanation is that these isolates may have contained the hemolysin genes and T3SS2 genes and may have lost them following passage through a host or during passage in the laboratory. This was previously observed for the enteropathogenic *Escherichia coli* isolate E2348/69, which exhibited loss of the EPEC virulence plasmid in a subset of culturable isolates following passage through adults in a clinical trial (Levine et al., 1985). Also, it may be possible that some of these *V. parahaemolyticus* isolates have as yet uncharacterized virulence factors. Further research is necessary to determine whether these *V. parahaemolyticus* clinical isolates are capable of causing disease without the hemolysin genes and/or T3SS2 genes. These findings highlight the many combinations of virulence-associated genes in *V. parahaemolyticus* clinical isolates, demonstrating the dynamic nature of the virulence repertoires of *V. parahaemolyticus* isolates.

**Table 2:** **Genome characteristics and *in silico* detection of virulence-associated genes in the sequenced *V. parahaemolyticus* genomes**.

								Hemolysin		T3SS2		Post-1995 O3:K6 isolate RIMD2210633 genomic islands^b^							Other virulence-associated regions			
Strain Id	Serotype	Year	Isolate source	Sample type^a^	Accession no.	No. contigs	Genome size (Mb)	*tdh*	*trh*	T3SS2α (VPaI-7)	T3SS2β	VPaI-1 (VP0380-VP0403)	VPaI-2 (VP0635-VP0643)	VPaI-3 (VP1071-VP1094)	VPaI-4 (VP2131-VP2144)	VPaI-5 (VP2900-VP2910)	VPaI-6 (VPA1253-VPA1270)	VPaI-7 (VPA1312-VPA1398)	f237 (VP1549-VP1562)	T6SS1 (VP1386-VP1414)	T6SS2 (VPA1025-VPA1046)	Super- integron (VP1787-VP1865)
Post-1995 O3:K6 Clinical Isolates																						
RIMD2210633	O3:K6	1996	Thailand (Japan airport)	Clinical (NK)	NC_004603, NC_004605	2	5.15	+	−	+	−	+	+	+	+	+	+	+	+	+	+	+
K5030	O3:K6	2005	India	Clinical (NK)	ACKB00000000	164	5.03	+	−	+	−	+	+	^c^+	+	+	+	+	+	+	+	+
AN5034^d^	O4:K68	1998	Bangladesh	Clinical (NK)	ACFO00000000	54	5.20	+	−	+	−	+	+	+	+	+	+	+	+	+	+	+
Peru−466	O3:K6	1996	Peru	Clinical (NK)	ACFM00000000	149	5.04	+	−	+	−	+	+	^c^+	+	+	+	+	+	+	+	+
Pre−1995 O3:K6 Clinical Isolates																						
AQ4037	O3:K6	1985	Maldive Islands	Clinical (NK)	ACFN00000000	164	4.94	−	+	−	+	−	−	−	−	−	−	−	−	+	+	−
AQ3810	O3:K6	1983	Singapore	Clinical (NK)	AAWQ00000000	1037	5.77	+	−	+/−	−	−	−	+/−	−	−	−	+/−	−	−	+	−
10329	O4:K12	1998	Washington, USA	Clinical (NK)	AFBW00000000	33	5.09	+	+	−	+	−	−	−	−	−	−	−	−	+/−	+	−
K1275	O3:K54	2004	Texas, USA	Clinical (blood)	JMMP00000000	63	5.11	−	−	−	−	−	+/−	−	−	−	−	−	−	+/−	^c^+	−
K1461	O4:K12	2004	Massachusetts, USA	Clinical (stool)	JMMO00000000	65	5.17	+	+	−	+	−	−	−	−	−	−	−	−	+/−	^c^+	−
Environmental Isolates																						
AF91	O3:K6	2006	Florida, USA	Environmental (sediment)	JMMS00000000	111	5.17	−	−	−	−	−	−	+/−	−	−	−	−	−	+/−	^c^+	+/−
BB22OP	O4:K8	1980s	Bangladesh	Environmental (NK)	CP003972, CP003973	2	5.10	+	−	+/−	−	−	+/−	−	−	−	−	+/−	−	+/−	+	−
22702	O5:Kuk	1998	Georgia, USA	Environmental (sediment)	JMMT00000000	43	4.95	−	−	−	−	−	−	−	−	−	−	−	+/−	−	+	−
J−C2−34	O5:K19	1998	North Carolina, USA	Environmental (sediment)	JMMR00000000	91	5.15	−	−	−	−	−	−	−	−	−	−	−	−	−	+	−
SG176	O5:Kuk	2006	Georgia, USA	Environmental (water)	JMMQ00000000	48	4.95	−	−	−	−	−	−	−	−	−	−	−	+/−	−	^c^+	−

a NK, not known;

b A + indicates a gene or all genes within a region were detected with BSR values ≥0.8, a +/− indicates that at least half of the genes were detected with BSR values ≥0.8;

c All genes were detected with BSR values ≥0.8, except one gene that was <0.8;

d *This isolate was described as having seroconverted from O3:K6 to O4:K68 (Chen et al., 2011)*.

### Molecular analysis of the genetic similarity of clinical and environmental *V. parahaemolyticus*

**Figure 1:**
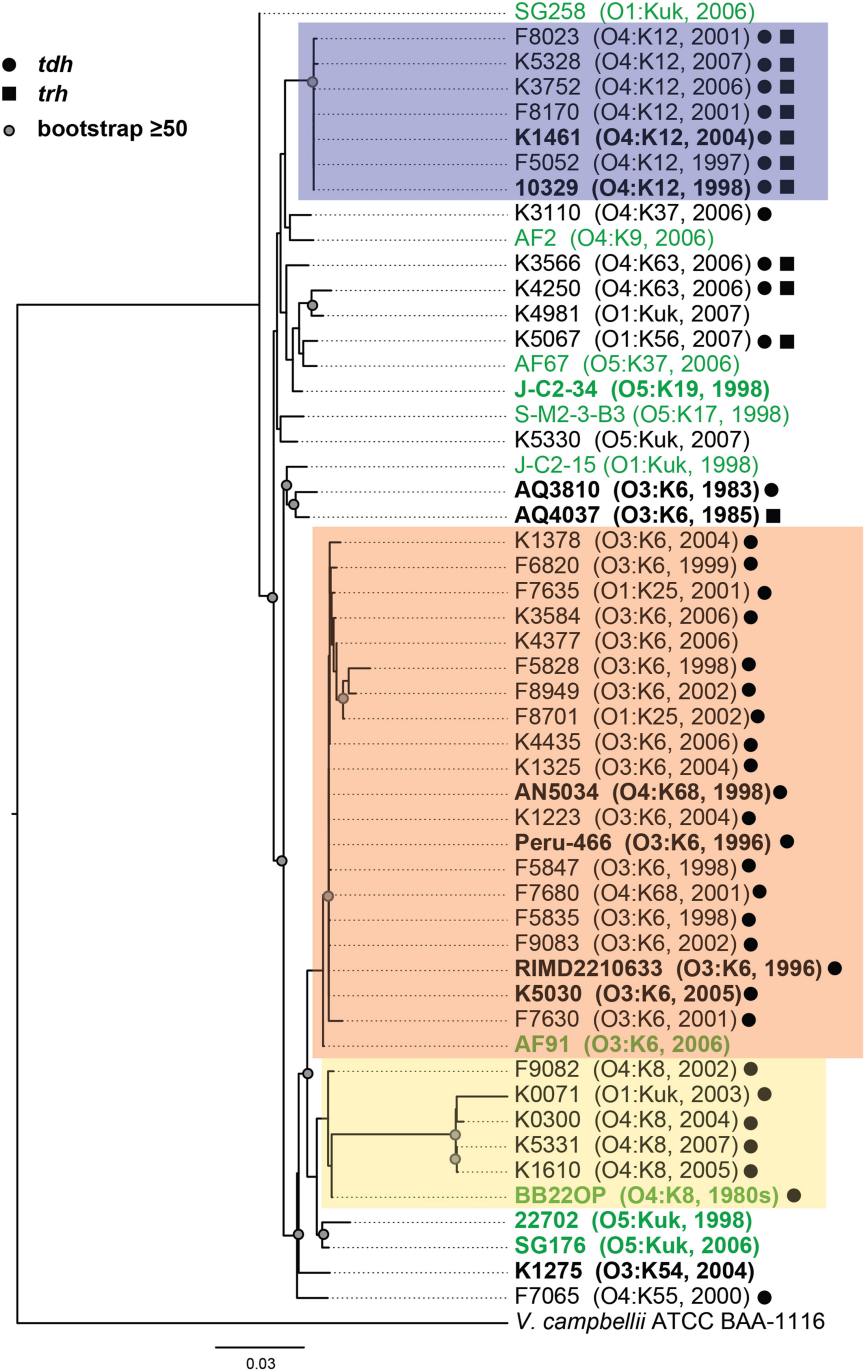
**Maximum-likelihood phylogeny of *V. parahaemolyticus* clinical and environmental isolates analyzed in this study compared to *V. parahaemolyticus* isolates that have been previously characterized by complete or draft genome sequencing and are available in the public domain**. The nucleotide sequences of seven conserved genes (*recA, gyrB, pyrC, dtdS, tnaA, dnaE*, and *pntA*) were concatenated for each *V. parahaemolyticus* isolate, and *V. campbellii* ATCC BAA-1116 was included as an outgroup. The phylogeny was constructed using RAxML (Stamatakis, 2006) with 100 bootstrap replications, and visualized using FigTree v1.3.1 (http://tree.bio.ed.ac.uk/software/figtree/). Only bootstrap values ≥50 are shown. The scale bar represents 0.03 nucleotide substitutions per site. The genomes that were sequenced in this study or in previous studies that are available in the public domain are indicated in bold and are listed in Table 2. The post-1995 *V. parahaemolyticus* O3:K6 isolates are indicated by an orange box, the O4:K12 isolates are indicated by a purple box, and the O4:K8 isolates are indicated in yellow. The *V. parahaemolyticus* isolates obtained from environmental sources are indicated in green, while the isolates from clinical sources are indicated in black. The presence of the virulence-associated thermostable direct hemolysins, *tdh* and *trh*, in each of the genomes is indicated by symbols.

Phylogenetic analysis of housekeeping genes was used to investigate the genetic similarity of *V. parahaemolyticus* clinical and environmental isolates representing diverse serotypes, isolation sources, and date of isolation (Figure 1, Supplemental Table 1). This approach has previously been used to investigate the evolutionary relationships of isolates within a single *Vibrio* species (Chowdhury et al., 2004; Boyd et al., 2008; González-Escalona et al., 2008; Turner et al., 2013), and among isolates belonging to multiple *Vibrio* species (Thompson et al., 2005, 2007, 2008; Sawabe et al., 2007, 2013; Lin et al., 2010). A phylogeny analyzing the genetic relatedness of 52 *V. parahaemolyticus* isolates (42 clinical, 10 environmental) was constructed using the partial nucleotide sequences of seven housekeeping genes (Figure 1). The phylogeny contained three distinct clades (colored boxes), which were primarily comprised of isolates with the O4:K12, O3:K6, and O4:K8 serotypes (Figure 1). Notably, all but two of the isolates (AF91 and BB22OP) that formed these three clades were derived from clinical sources (Figure 1). The other 18 isolates analyzed that were outside of these three clades included a mixture of clinical and environmental isolates that have diverse serotypes, and these isolates exhibited considerable phylogenetic diversity (Figure 1). Furthermore, this demonstrated that clinical isolates with serotypes other than O3:K6, O4:K12, and O4:K8 had genetic similarity to the environmental isolates analyzed in this study (Figure 1).

To further investigate the genetic diversity observed for the clinical and environmental isolates that had serotypes other than O3:K6, O4:K12, and O4:K8, we analyzed partial sequences of two housekeeping genes of chromosome I (*recA* and *gyrB*), and two housekeeping genes of chromosome II (*pyrC* and *dtdS*) in a larger collection of 116 *V. parahaemolyticus* clinical and environmental isolates (Supplemental Figure 1). In a phylogenetic analysis of the concatenation of all four genes, there were four clinical isolates that formed a sub-clade with a long branch. To investigate whether the long branch of this sub-clade resulted from sequence divergence within a particular analyzed gene we generated individual phylogenies for each gene. Three of these genes (*gyrB, pyrC*, and *dtdS*) had similar topologies to the concatenated phylogeny, while the *recA* phylogeny demonstrated there was additional sequence divergence within *recA* for the four clinical isolates with the longer branch. Therefore, we analyzed the diversity of these four housekeeping genes by constructing a phylogeny for three of the genes (Supplemental Figure 1A), compared with a separate phylogeny of only *recA* sequences (Supplemental Figure 1B). Overall, phylogenetic analysis of the three genes (*gyrB, pyrC, dtdS*) indicated the clinical and environmental isolates analyzed have extensive genetic diversity (Supplemental Figure 1). Previous studies have demonstrated there is considerable genetic diversity of *V. parahaemolyticus* isolates from around the world (Chowdhury et al., 2004; González-Escalona et al., 2008).

The genetic relatedness of *V. parahaemolyticus* clinical and environmental isolates was also investigated using PFGE, which is a cost-effective method for routine identification of disease-associated bacteria, including *V. parahaemolyticus* (Parsons et al., 2007). PFGE was performed on a total of 37 clinical isolates and 7 environmental isolates (Supplemental Figure 2). Analysis of the *Sfi*I and *Not*I patterns of these isolates demonstrated the presence of three main clades corresponding to those identified based on phylogenetic analysis of the housekeeping genes and the phylogenomic analysis (Figure 1, Supplemental Figure 1). A notable exception is that of the O3:K6 environmental isolate, AF91, which was not present in the O3:K6 clade by PFGE analysis as it was in the housekeeping gene phylogenies (Figure 1, Supplemental Figure 1). Similar to the housekeeping gene phylogenies, PFGE also demonstrated there is considerable genetic diversity among the clinical and environmental isolates analyzed that had serotypes other than those of the three main clades (O3:K6, O4:K12, and O4:K8) (Supplemental Figure 2). The PFGE pattern of the *V. parahaemolyticus* environmental isolate AF91 was different from the O3:K6 clinical isolates and the other environmental isolates examined. The *NotI* pattern of AF91 was similar to that of other *V. parahaemolyticus* isolates; however, the *Sfi*I pattern had multiple large bands that ranged from approximately 485- to 693-kb. In addition, the *Sfi*I pattern of this strain was missing four or more small bands that were present in the *Sfi*I patterns of the other *V. parahaemolyticus* isolates. The presence of the larger bands in the *Sfi*I pattern of AF91 suggested the absence of several *Sfi*I restriction sites that may correlate with the absence of the genomic islands of the post-1995 O3:K6 isolates (Hurley et al., 2006).

### Comparative genomics of clinical and environmental *V. parahaemolyticus*

**Figure 2:**
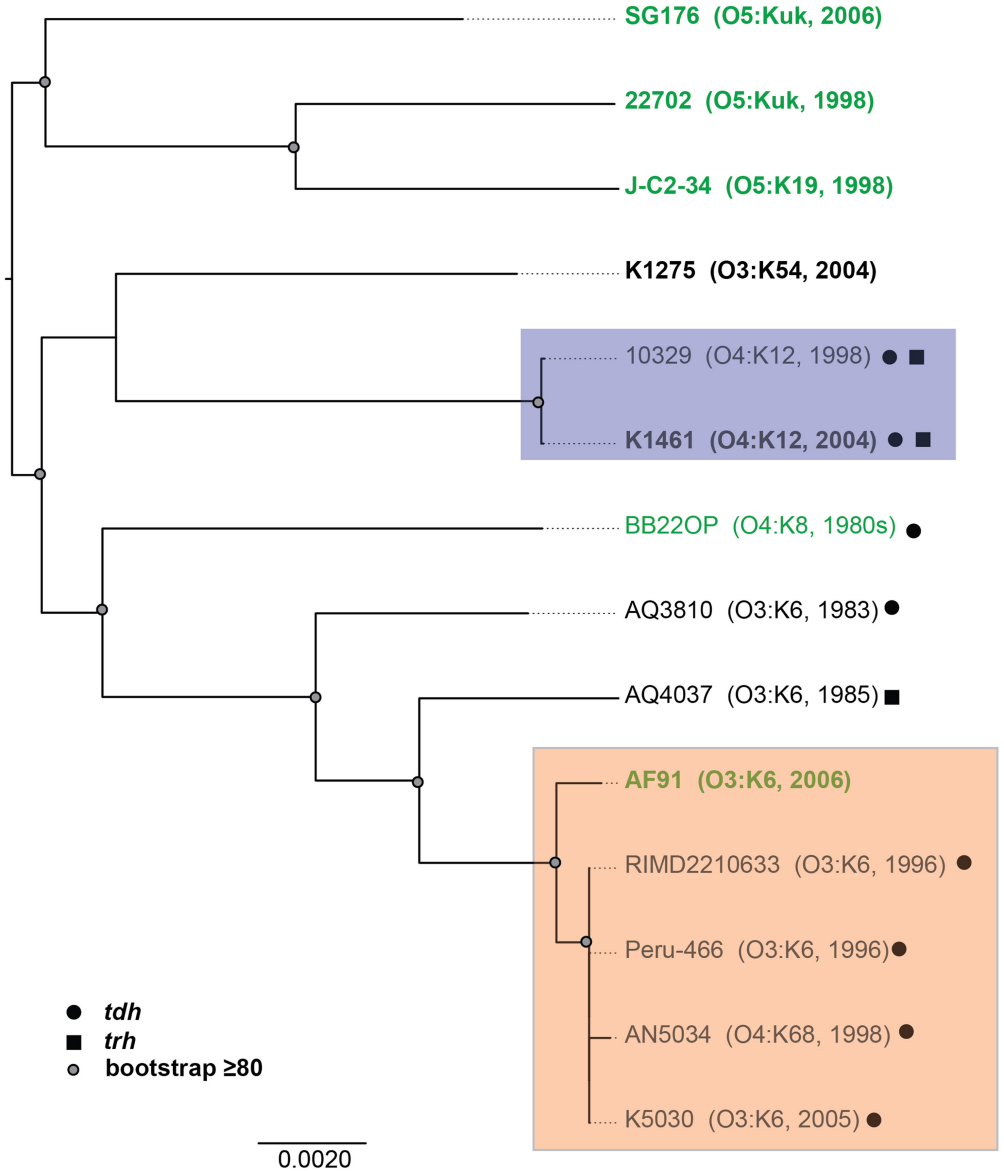
**Phylogenomic analysis of the six *V. parahaemolyticus* clinical and environmental isolates sequenced in this study (indicated in bold) compared to eight *V. parahaemolyticus* genomes that have been previously sequenced (Makino et al., 2003; Boyd et al., 2008; Chen et al., 2011; Gonzalez-Escalona et al., 2011; Jensen et al., 2013) and are available in the public domain**. The genomes were aligned using Mugsy (Angiuoli and Salzberg, 2011), and an approximately 4.0 Mb region of each genome that aligned was concatenated to generate a single sequence for each isolate as previously described (Sahl et al., 2011). A maximum-likelihood phylogeny with 100 bootstrap replicates was constructed using RAxML (Stamatakis, 2006) and visualized using FigTree v1.3.1 (http://tree.bio.ed.ac.uk/software/figtree/). Bootstrap values ≥80 are indicated by a circle. The genomes of *V. parahaemolyticus* isolates from environmental sources are indicated in green, while the isolates from clinical sources are indicated in black. The post-1995 O3:K6 isolates are identified by the orange box, and the O4:K12 isolates are indicated by a purple box. The presence of the virulence-associated thermostable direct hemolysins, *tdh* and *trh*, in each of the genomes is indicated by symbols.

To investigate whether there are shared or exclusive genome features of *V. parahaemolyticus* clinical and environmental isolates, we generated high-quality draft genome sequences of six *V. parahaemolyticus* isolates (K1461, K1275, SG176, J-C2-34, AF91, and 22702) that had diverse isolation sources, serotypes, and virulence factor content (Supplemental Table 1). Phylogenomic analysis of the six *V. parahaemolyticus* genomes sequenced in this study compared to previously sequenced *V. parahaemolyticus* genomes (Table 2) demonstrated there is considerable genomic diversity among isolates from clinical and environmental sources (Figure 2). Three of the environmental isolate genomes (22702, SG176, and J-C2-34) grouped together in the whole-genome phylogeny, while the other two environmental isolate genomes (AF91, BB22OP (Jensen et al., 2013) were within a larger group that contained the clinical isolate genomes (Figure 2). The phylogenomic analysis further confirmed that the O3:K6 environmental isolate, AF91, was more related to the post-1995 O3:K6 genomes than to the pre-1995 O3:K6 isolate genomes that have been previously sequenced (Figure 2).

*In silico* identification of the known *V. parahaemolyticus* virulence-associated genes and genomic islands (Hurley et al., 2006; Boyd et al., 2008) in the clinical and environmental genomes sequenced demonstrated that these regions were primarily identified in the post-1995 O3:K6 genomes (Table 2). However, some but not all of the genes in a few of these regions (VPaI-2, VPaI-3, and VPaI-7 encoding T3SS2α) were identified in some of the other clinical or the environmental isolate genomes (Table 2). This finding is similar to previous studies that demonstrated the T6SS gene cluster of chromosome I (T6SS1) is more frequently associated with *V. parahaemolyticus* clinical isolates than environmental isolates (Yu et al., 2012). The genes of T6SS1 were identified in nearly all the clinical isolate genomes, except the pre-1995 O3:K6 isolate AQ3810, and they were not identified in the genomes of the environmental isolates except for AF91 and BB22OP, which encode genes with similarity to those of T6SS1 (Table 2). However, AF91 and BB22OP have the serotypes O3:K6 and O4:K8, respectively, which are serotypes that have been linked to cases of human illness (Okuda et al., 1997; Matsumoto et al., 2000; Chowdhury et al., 2013; Ma et al., 2014). Further investigation is necessary to determine whether these environmental isolates may be more likely to cause disease than other environmental isolates that do not possess the T6SS1 genes.

Comparison of the *V. parahaemolyticus* genome content was analyzed using large-scale BLAST score ratio (LS-BSR) (Sahl et al., 2014) and further demonstrated the extent of the overall genome similarity among the clinical and environmental isolate genomes analyzed (Table 3). There were a total of 7782 genes identified in the 14 genomes analyzed in this study, 3494 of these genes were present with significant similarity (LS-BSR value ≥0.8) in all of the genomes analyzed (Table 3). Of the total genes identified there were 755 that were present in one or more of the clinical isolate genomes with significant similarity (LS-BSR value ≥0.8) that were not identified (LS-BSR value <0.4) in any of the environmental genomes sequenced (Table 3, Supplemental Table 2). Among these were genes encoding T3SS proteins, which likely belong to T3SS2 since these genes were not identified in any of the environmental isolates sequenced (Table 2). Also included among these genes were a multidrug resistance efflux pump and a putative RTX toxin (Supplemental Table 2). There were a similar number of genes (838) that were identified in one or more of the environmental isolate genomes that were not identified in any of the clinical isolate genomes (Table 3, Supplemental Table 2).

**Table 3:** **LS-BSR analysis of the genomic similarity of select *V. parahaemolyticus* clinical and environmental isolates**.

**Genomes**	**No. of genomes**	**No. of gene clusters**	
		**All**	**≥ 1^c^**
All genomes analyzed	14	3473	7782
All clinical isolate genomes	9	0 (26)^b^	755 (407)^d^
All environmental isolate genomes	5	0 (20)^b^	838 (230)^d^
Clinical Isolate Genomes			
Post-1995 O3:K6 (including AF91)^a^	5	17	423
Post-1995 O3:K6 (not including AF91)^a^	4	78	198
Pre-1995 O3:K6	2	26	391
O4:K12	2	169	244

a *This comparison group includes an O4:K68 isolate that is a seroconversion from O3:K6, and the environmental isolate AF91 where indicated in parentheses*.

b *Genes are highly conserved (LS-BSR ≥0.8) in all genomes of the group and divergent (LS-BSR <0.8, ≥0.4) or absent (LS-BSR < 0.4) in the other genomes. The number of gene clusters in parentheses is conserved in all genomes when BB22OP and AF91 are not included*.

c *Genes that are highly conserved (LS-BSR ≥ 0.8) in one or more of the genomes of the group and divergent or absent (LS-BSR < 0.8) in the other genomes*.

d *The number of gene clusters that are highly-conserved (LS-BSR ≥0.8) in one or more genomes of this group, and absent (LS-BSR <0.4) from the other genomes. The number in parentheses is the number of gene clusters that are highly-conserved (LS-BSR ≥0.8) in one or more genomes of this group, but are divergent (LS-BSR <0.8, ≥0.4) in one or more of the other genomes*.

There were no genes identified in all of the clinical isolate genomes that were not present in one or more of the environmental isolate genomes, or vice versa (Table 3). This is likely due to significant genetic similarities between clinical and environmental isolate genomes such as the O3:K6 environmental isolate AF91 and the O3:K6 clinical isolates (Figures 1, 2). The inability to identify genes that are exclusive to all clinical isolate genomes also can likely be attributed to the inclusion of the environmental isolate BB22OP, which encodes known virulence-associated genes such as *tdh* (Jensen et al., 2013). However, upon exclusion of the AF91 and BB22OP genomes from the analysis, there were 26 genes that were highly-conserved (LS-BSR values ≥0.8) in all clinical isolate genomes that were divergent (LS-BSR values <0.8, ≥0.4) or absent (LS-BSR values < 0.4) from the three remaining environmental isolate genomes (22702, J-C2-34, SG176), and 20 that were highly-conserved (LS-BSR values ≥0.8) in the three environmental isolate genomes that were divergent (LS-BSR values <0.8, ≥0.4) or absent (LS-BSR values <0.4) from the clinical isolate genomes (Table 3). The small number of genes that were universal to clinical or environmental isolates could also be a result of the genetic diversity or misclassification of the clinical and environmental isolates (Figures 1, 2).

The number of genes that were exclusive (LS-BSR values ≥0.8, and <0.4 in all other genomes) to the six *V. parahaemolyticus* genomes sequenced in this study ranged from 20 to 173 (Supplemental Table 3). The fewest number of exclusive genes (20) was identified in the genome of the O4:K12 isolate K1461, which can be attributed to the significant genomic similarity of this isolate to the previously sequenced genome of the O4:K12 isolate 10329 (Gonzalez-Escalona et al., 2011) (Figure 2). The genome of the *V. parahaemolyticus* clinical isolate that was *tdh−/trh−/*T3SS2−, K1275, encoded 150 genes that were exclusive to this isolate (Supplemental Table 3). Among these unique genes were many hypothetical proteins and other genes that lacked similarity to any previously characterized genes, which suggests there is extensive genomic diversity that has yet to be characterized from *V. parahaemolyticus* isolates (Supplemental Table 3).

Many of the genes identified as exclusive to a particular genome were hypothetical or were similar to genes of mobile genetic elements including plasmids and phage (Supplemental Table 3), highlighting the contribution of mobile elements such as plasmids and phage to the diversification of *V. parahaemolyticus*. In addition to being present in the post-1995 O3:K6 genomes, the protein-encoding genes of the filamentous vibriophage f237 of RIMD2210633 were identified in the genomes of two environmental isolate genomes (22702, SG176) (Table 2). Sequence analysis of partial nucleotide sequences of the vibriophage replication protein-encoding gene, *rstA* obtained from *V. parahaemolyticus* clinical and environmental isolates revealed these sequences had 95–100% nucleotide identity to the *rstA* of the filamentous phage f237 (Nasu et al., 2000). Phylogenetic analysis of the partial nucleotide sequences of *rstA*, demonstrated there is no discernible pattern of genetic similarity of the filamentous vibriophage based on serotype, isolation source, or geographical location, with the exception of the O4:K12 isolates and the post-1995 O3:K6 isolates (Supplemental Figure 3).

In addition to the identification of genetic similarity of filamentous vibriophage from clinical and environmental isolates, there was an approximately 90-kb phage-like element identified in the genome sequences of the O4:K12 clinical isolate K1461, and the environmental isolate and J-C2-34. Analysis of the plasmid content of K1461 and J-C2-34 using a modified acid-phenol extraction method (Kieser, 1984; Sobecky et al., 1997) demonstrated that both of these isolates contain a single large extrachromosomal element that is approximately 90-kb, which is likely the prophage identified in the genomes of these isolates. Sequence characterization of these prophage demonstrated they encode numerous phage-like genes with a conserved organization that exhibited 80–100% nucleotide identity to each other (Supplemental Figure 4). This finding provides additional evidence of the horizontal transfer of similar prophage-like elements among *V. parahaemolyticus* clinical and environmental isolates. These phage-like elements also exhibited divergent similarity (BSR values ≥0.4, <0.8) to genes encoded by a previously sequenced plasmid, p0908, from *V. fluvialis* (Hazen et al., 2007) and the bacteriophage P1 (Lobocka et al., 2004), suggesting they belong to a phage family that has relatives in other enteric bacteria.

### Genomic similarity of the O3:K6 environmental isolate, AF91, to pre- and post-1995 O3:K6 isolates

**Figure 3:**
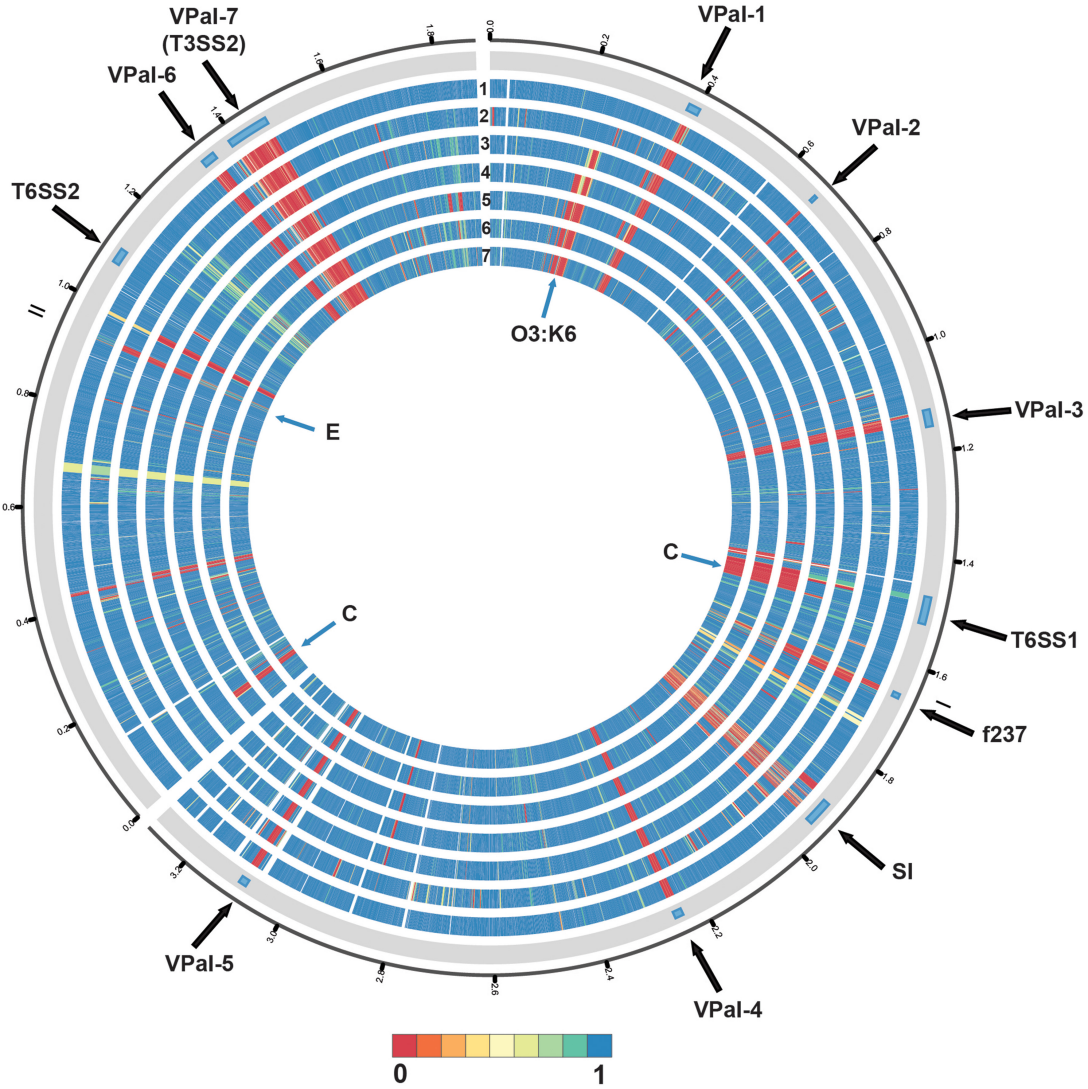
**Circular display of the BLASTN BSR values demonstrating the genetic similarity of each gene of RIMD2210633 in the genome of the pre-1995 O3:K6 isolate AQ4037 (Chen et al., 2011), and the genomes sequenced in this study**. The previously described putative pathogenicity islands (Hurley et al., 2006) and other regions of interest (Chen et al., 2011) in the genome of RIMD2210633 (Makino et al., 2003) are indicated in the outermost track. The genomes in tracks labeled 1–7 are as follows: (1) AF91, (2) AQ4037, (3) K1275, (4) K1461, (5) SG176, (6) J-C2-34, and (7) 22702. The location of the O3:K6 O-antigen lipopolysaccharide region is indicated by “O3:K6.” An “E” designates the location of RIMD2210633 genes that are present with greater similarity in the environmental isolate genomes than in the clinical isolate genomes. A “C” designates the location of RIMD2201633 genes that are present in the clinical isolate genomes analyzed and not in the environmental isolate genomes, with the exception of AF91. The circular display of the BSR values was generated using Circos (Krzywinski et al., 2009). Blue indicates genes were present with significant similarity, yellow indicates the genes were present but divergent, and red indicates genes were absent.

Phylogenomic analysis demonstrated that the 2006 O3:K6 environmental isolate, AF91, exhibited greater genomic similarity to the post-1995 O3:K6 isolate genomes (Makino et al., 2003; Chen et al., 2011) than to the two pre-1995 O3:K6 isolate genomes (Boyd et al., 2008; Chen et al., 2011) (Figure 2). A PCR-based assay and *in silico* analysis of the AF91 genome demonstrated that AF91 does not encode the hemolysins or T3SS2 that are typically found in *V. parahaemolyticus* clinical isolates (Table 2, Supplemental Table 1). The AF91 genome was also missing most of the genomic islands of the post-1995 O3:K6 isolate RIMD2210633 (Table 2, Figure 3). However, AF91 did contain genes with similarity to those encoded by the genomic island VPaI-3 (Table 2), which was previously identified in post-1995 O3:K6 isolates and related isolates (AN5034) that have undergone seroconversion (Boyd et al., 2008; Chen et al., 2011) (Table 2). AF91 also encoded genes with significant similarity to genes of T6SS1 (Table 2, Figure 3), which has primarily been identified in *V. parahaemolyticus* clinical isolates (Yu et al., 2012). Their findings demonstrated that the T6SS1 genes exhibited bacteriolytic activity against other bacteria when grown in conditions similar to the marine environment, suggesting T6SS1 provides a fitness advantage that allows the disease-associated isolates to be competitive and persist in marine environments (Salomon et al., 2013). T6SS1 may have contributed to the emergence and spread of the post-1995 O3:K6 isolates (Okuda et al., 1997; Matsumoto et al., 2000).

Comparative analysis of the O3:K6 genomes using LS-BSR demonstrated there were only 17 gene clusters identified in all post-1995 O3:K6 isolates, including the O3:K6 environmental isolate AF91 (Table 3) that were divergent (LS-BSR value <0.8, ≥0.4) or absent (LS-BSR value <0.4) from the other genomes. However, there were 78 gene clusters present with significant similarity in all of the post-1995 O3:K6 isolate genomes when AF91 was not included (Table 3). This finding demonstrates that although AF91 has the O3:K6 serotype and was isolated a decade after the emergence and spread of the O3:K6 pandemic clone (Okuda et al., 1997; Vuddhakul et al., 2000; Myers et al., 2003; Quilici et al., 2005; Ottaviani et al., 2008), the genome of AF91 exhibits genetic differences compared to other post-1995 O3:K6 isolate genomes. Not surprisingly, many of these genes were encoded within the genomic regions that were previously described as unique to the post-1995 O3:K6 genomes (Hurley et al., 2006; Chen et al., 2011) (Supplemental Table 4). In addition to the 78 genes that are divergent or missing (LS-BSR value <0.8) from the AF91 genome compared to other post-1995 O3:K6 genomes, there were 173 genes that were identified by LS-BSR as being unique to the AF91 genome compared to the other genomes analyzed (Table 3). The genes that were unique to AF91 included integrases and transposases, putative transcriptional regulators, a GGDEF domain-containing protein, and many genes encoding proteins with unknown functions (Supplemental Table 3).

This study describes an environmental O3:K6 isolate that exhibits significant genetic similarity to the post-1995 O3:K6 isolates, yet does not encode most of the known virulence-associated genes of these isolates. Due to the genomic similarity of this O3:K6 environmental isolate to the post-1995 O3:K6 isolates, this isolate may have originally carried the missing genomic islands, and after entering the environment may have begun to transition to an environmental niche by losing the virulence-associated genomic regions. For example, genomic islands of the uropathogenic *Escherichia coli* isolate 536, a strain isolated from a urinary tract infection, were demonstrated to be unstable (Middendorf et al., 2004; Soto et al., 2006). Interestingly, two of the *E. coli* 536 genomic islands, including one island that encoded a hemolysin, were lost in response to altered environmental conditions such as lower temperature and higher cell density (Middendorf et al., 2004). Further experiments would be necessary to determine whether AF91 would have a fitness advantage over other *V. parahaemolyticus* environmental isolates, and whether the absence of the other post-1995 O3:K6 genomic islands and virulence-associated regions gives it an additional advantage for surviving in the environment. Another possibility is that AF91 may represent an intermediate isolate that was involved in the emergence of the post-1995 O3:K6 isolates, and AF91 may have persisted without acquiring the post-1995 O3:K6 genomic islands due to a fitness advantage for surviving in the environment. Further experiments are needed to determine if AF91 would have a similar pathogenic potential as other post-1995 O3:K6 isolates, following the acquisition of the post-1995 O3:K6 genomic islands and other virulence-associated regions.

Previous research investigating the disease-causing potential of *V. parahaemolyticus* in the environment has typically examined the presence of prevalent clinical serotypes and known virulence-associated genes. However, we have demonstrated that investigating the genetic diversity of environmental isolates that do not carry the known virulence-associated genes can yield insight into the emergence of human disease-associated *V. parahaemolyticus*. Sequencing and characterization of *V. parahaemolyticus* AF91, an O3:K6 environmental isolate, demonstrated that environmental isolates that do not carry the known virulence-associated genes can have significant genetic similarity to disease-associated *V. parahaemolyticus* clinical isolates, including the pandemic post-1995 O3:K6 isolates. Additional genome sequencing of *V. parahaemolyticus* clinical and environmental isolates that have diverse serotypes and unique combinations of known virulence-associated genes and genomic regions would yield further insight into the ability of *V. parahaemolyticus* isolates to transition from an environmental niche and to emerge as pathogens.

### Conflict of interest statement

The authors declare that the research was conducted in the absence of any commercial or financial relationships that could be construed as a potential conflict of interest.
