# Supplementary material for: Insights into the environmental reservoir of pathogenic Vibrio parahaemolyticus using comparative genomics
Source: Front Microbiol. 2015 Mar 24;6:204. doi: 10.3389/fmicb.2015.00204 (PMC4371758; doi:10.3389/fmicb.2015.00204)
Supplement: Supplementary file 2 [file DataSheet2.PDF]

Supporting Information

A.

B.

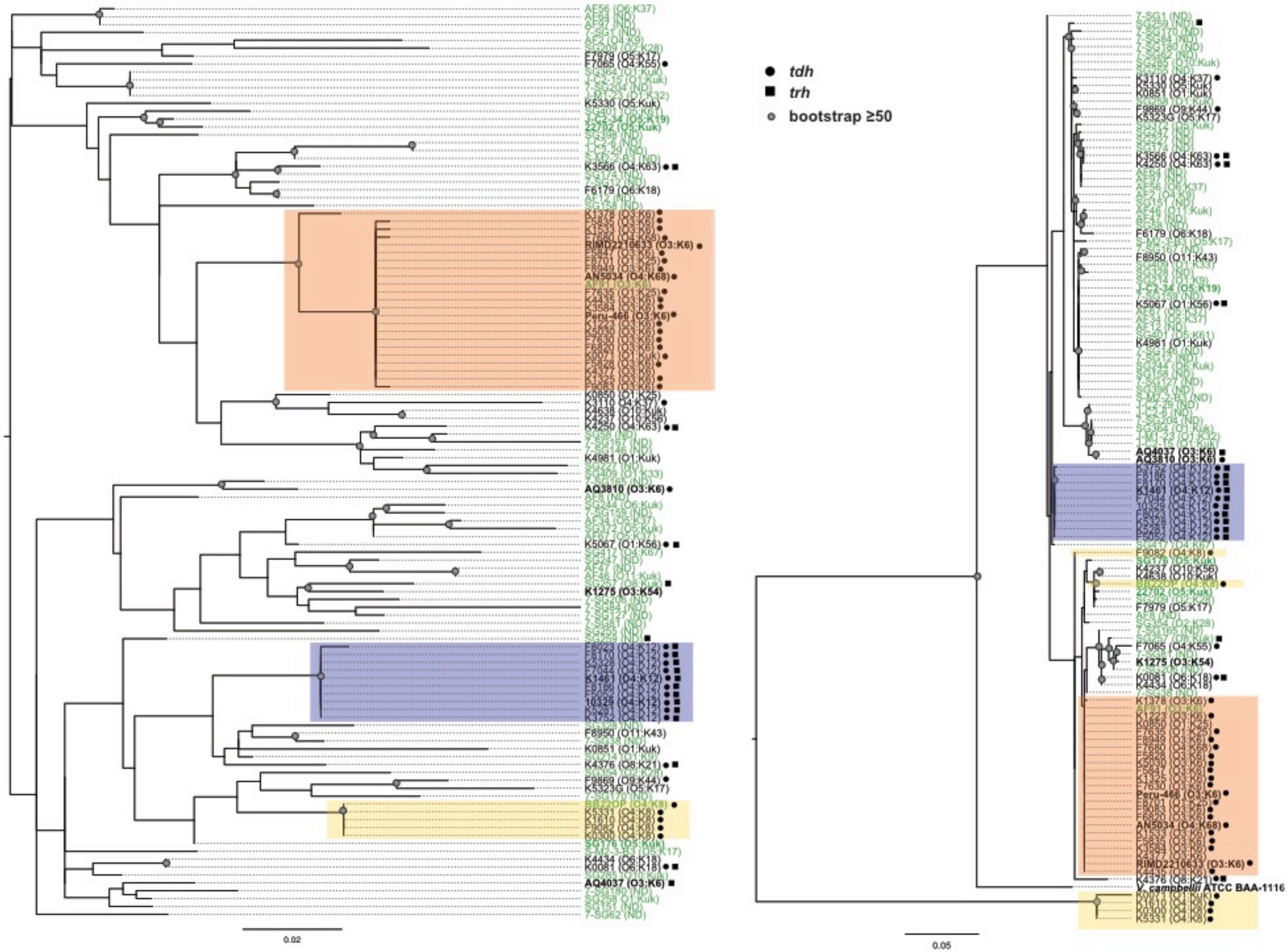

Supplemental Fig. 1

Supplemental Fig. 1. Maximum-likelihood phylogenies of **A)** three housekeeping genes that have been concatenated (*gyrB, pyrC,* and *dtdS*), compared to **B)** the phylogenetic analysis of *recA,* from select *V. parahaemolyticus* clinical and environmental isolates, and the housekeeping gene sequences from eight *V. parahaemolyticus* isolate genomes available in the public domain. The phylogeny was constructed using RAxML (Stamatakis, 2006) with 100 bootstrap replications, and visualized using FigTree v1.3.1 (http://tree.bio.ed.ac.uk/software/figtree/). Only bootstrap values ≥50 are shown. The scale bar represents 0.03 nucleotide substitutions per site. The genomes that were sequenced in this study or in previous studies that are available in the public domain are indicated in bold. The post-1995 *V. parahaemolyticus* O3:K6 isolates are indicated by an orange box, the O4:K12 isolates are indicated by a purple box, and the O4:K8 isolates are indicated in yellow. The *V. parahaemolyticus* isolates obtained from environmental sources are indicated in green, while the isolates from clinical sources are indicated in black. The presence of the virulence-associated thermostable direct hemolysins, *tdh* and *trh*, in each of the genomes is indicated by symbols.

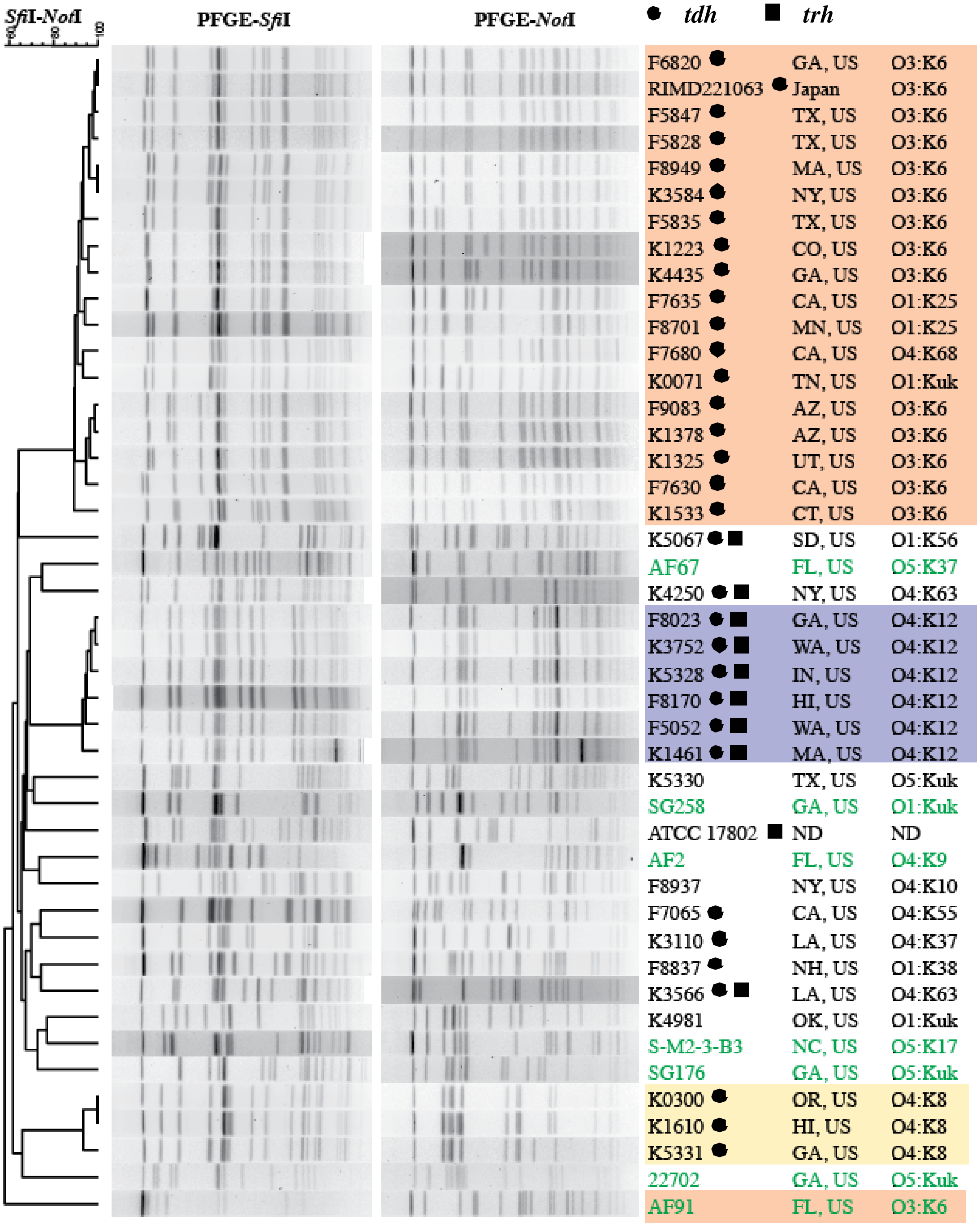

Supplemental Fig. 2

Supplemental Fig. 2. Dendrogram and PFGE patterns of *NotI* and *SfiI*-digested DNA from select *V. parahaemolyticus* clinical and environmental isolates examined in this study. The dendrogram was generated using BioNumerics v. 5.1 (Applied-Maths, Kortrijk, Belgium) with the Dice coefficient and unweighted pair group method with arithmetic averages (UPGMA) with a band position tolerance and optimization of 1.5% for cluster analysis. The genomes that were sequenced in this study or in previous studies that are available in the public domain are indicated in bold. The post-1995 *V. parahaemolyticus* O3:K6 isolates are indicated by an orange box, the O4:K12 isolates are indicated by a purple box, and the O4:K8 isolates are indicated in yellow. The *V. parahaemolyticus* isolates obtained from environmental sources are indicated in green, while the isolates from clinical sources are indicated in black. ND indicates that information was not determined. The presence of the virulence-associated thermostable direct hemolysins, *tdh* and *trh*, in each of the genomes is indicated by symbols.

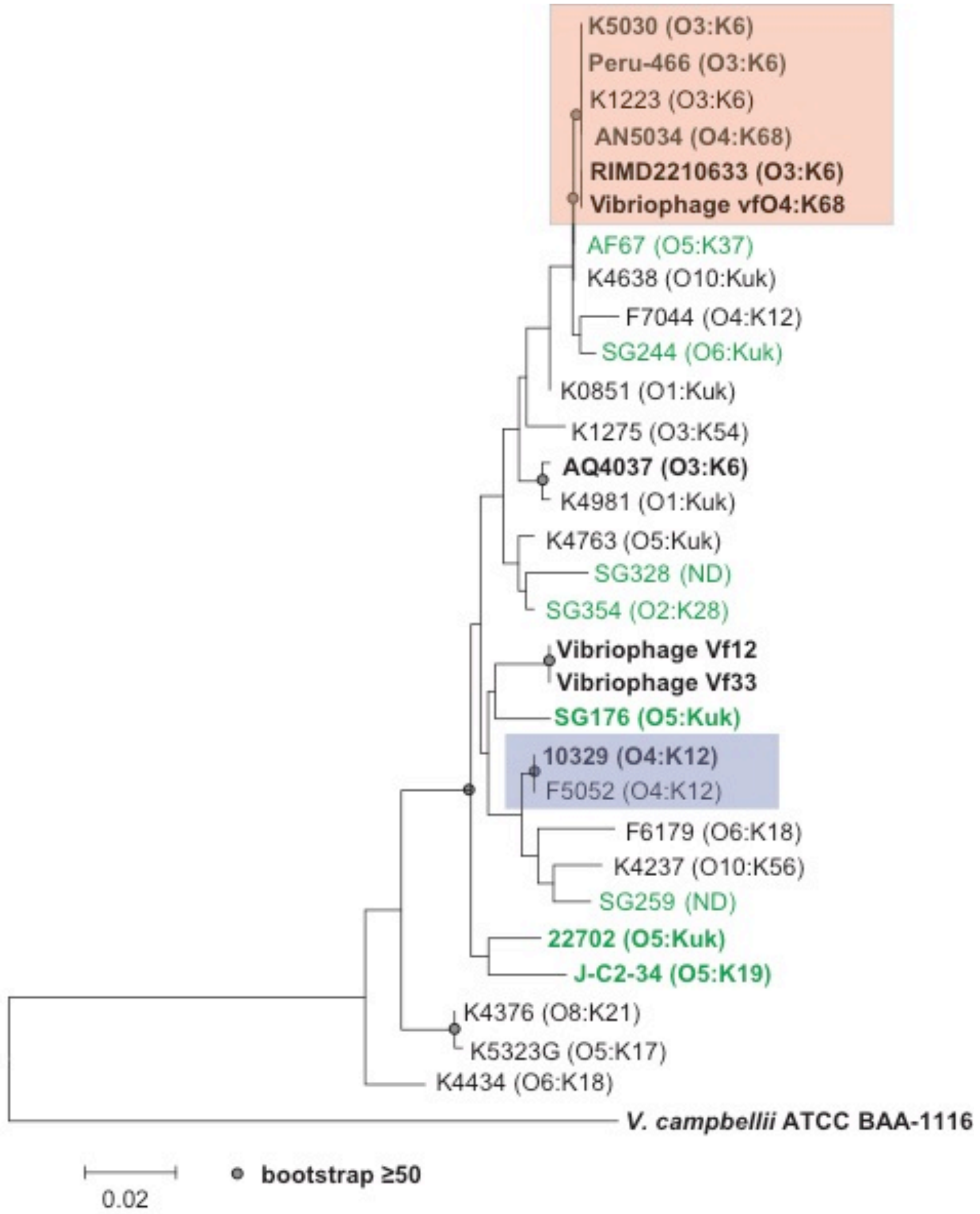

Supplemental Fig. 3

Supplemental Fig. 3. Phylogenetic analysis of partial nucleotide sequences that exhibit similarity to the replication protein encoding gene, *rstA,* of the filamentous vibriophage f237 (Nasu et al., 2000). A maximum-likelihood phylogeny was constructed with the Kimura 2-parameter model and 1,000 bootstrap replications using MEGA5 (Tamura et al., 2011). Bootstrap values ≥50 are shown. The scale bar indicates the approximate distance of 0.02 nucleotide substitutions per site. The post-1995 *V. parahaemolyticus* O3:K6 isolates are indicated by an orange box, and the O4:K12 isolates are indicated by a purple box. *V. parahaemolyticus* isolates obtained from environmental sources are indicated in green, while the isolates from clinical sources are indicated in black. Serotypes of each *V. parahaemolyticus* isolate are indicated in parentheses when known, otherwise ND indicates they were not determined. The *V. parahaemolyticus* isolates and vibriophages that have been characterized by genome sequencing either in the current study or previous studies are indicated in bold.

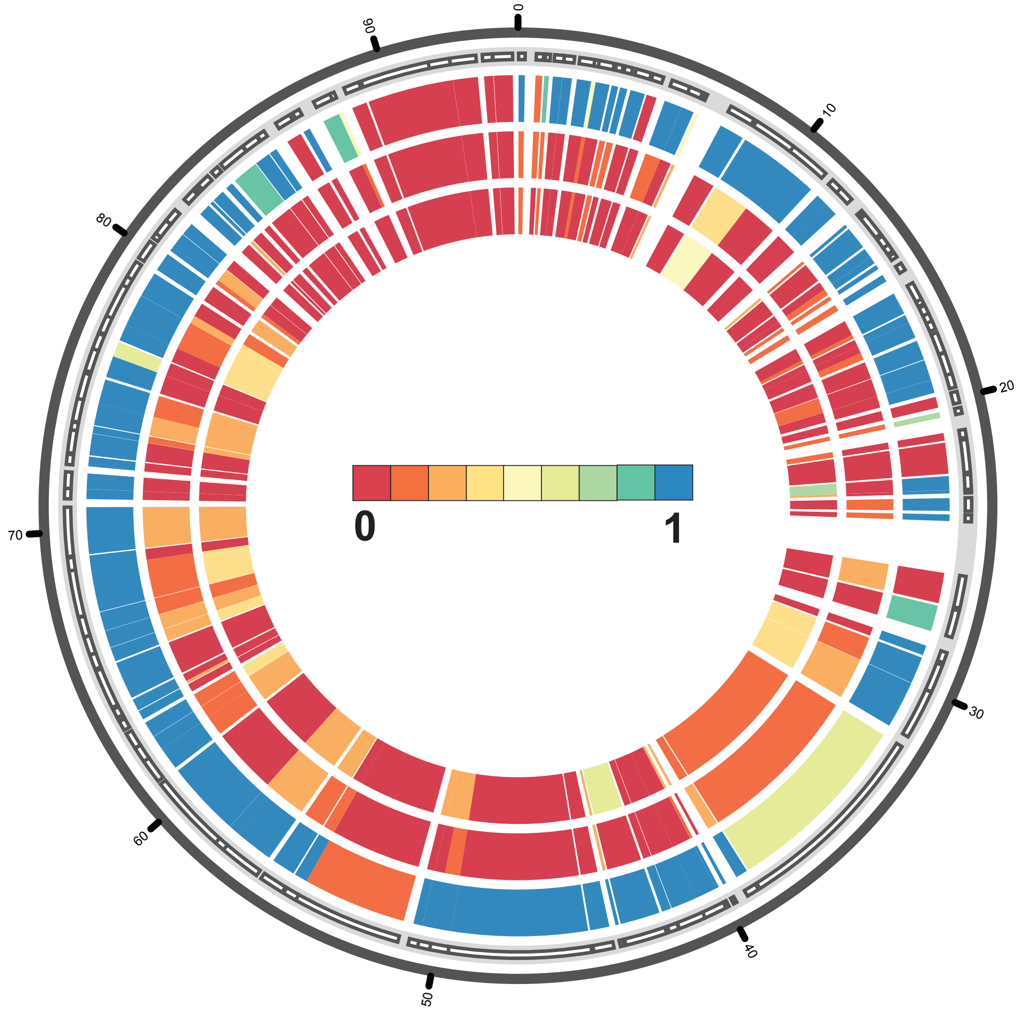

Supplemental Fig. 4

Supplemental Fig. 4. Circular display of the genetic similarity of protein-encoding genes (outermost track, track 1) from a prophage identified in the genome of the *V. parahaemolyticus* O4:K12 clinical isolate K1461, compared to the O5:K19 environmental isolate J-C2-34 (track 2), the phage-like plasmid p0908 from a *V. fluvialis* environmental isolate (track 3) (Hazen et al., 2007), and the bacteriophage P1 (track 4) (Lobocka et al., 2004). The amino acid sequences of genes encoded by the prophage of K1461 were compared to the prophage contig of J-C2-34, and the sequences of p0908 (NC_010113.1) and bacteriophage P1 (AF234172) as previously described (Rasko et al., 2005) using TBLASTN (Gertz et al., 2006). The presence of a gene with significant similarity is indicated by blue, and the absence of a gene with any similarity is indicated by red. The circular display of the BSR values was generated using Circos (Krzywinski et al., 2009).

Supplemental Table 1. *V. parahaemolyticus* isolates examined in this study

Isolate ID	Isolation Year	Location	Serotype	Sample[b]	*tl*	ORF8	*tdh*	*trh*	T3SS1	T3SS2α	T3SS2β
K1533	2004	Connecticut, USA	O3:K6	clinical (stool)	+	+	+	-	+	+	-
K1223	2004	Colorado, USA	O3:K6	clinical (stool)	+	+	+	-	+	+	-
K4435	2006	Georgia, USA	O3:K6	clinical (stool)	+	+	+	-	+	+	-
F9083	2002	Arizona, USA	O3:K6	clinical (stool)	+	+	+	-	+	+	-
F5847	1998	Texas, USA	O3:K6	clinical (NK)	+	+	+	-	+	+	-
K1325	2004	Utah, USA	O3:K6	clinical (stool)	+	+	+	-	+	+	-
F8949	2002	Massachusetts, USA	O3:K6	clinical (stool)	+	+	+	-	+	+	-
F5828	1998	Texas, USA	O3:K6	clinical (NK)	+	+	+	-	+	-	-
K3584	2006	New York, USA	O3:K6	clinical (stool)	+	+	+	-	+	+	-
F6820	1999	Georgia, USA	O3:K6	clinical (stool)	+	+	+	-	+	+	-
K4377	2006	Maryland, USA	O3:K6	clinical (stool)	+	+	-	-	+	+	-
F5835	1998	Texas, USA	O3:K6	clinical (NK)	+	+	+	-	+	+	-
F7630	2001	California, USA	O3:K6	clinical (NK)	+	+	+	-	+	+	-
K1378	2004	Arizona, USA	O3:K6	clinical (stool)	+	+	+	-	+	+	-
K5331	2007	Georgia, USA	O4:K8	clinical (stool)	+	-	+	-	+	+	-
K1610	2005	Hawaii, USA	O4:K8	clinical (stool)	+	-	+	-	+	+	-
F9082	2002	Arizona, USA	O4:K8	clinical (stool)	+	-	+	-	+	+	-
K0300	2004	Oregon, USA	O4:K8	clinical (stool)	+	-	+	-	+	+	-
F8186	2002	New York, USA	O4:K12	clinical (stool)	+	-	+	+	+	-	+
F8023	2001	Georgia, USA	O4:K12	clinical (stool)	+	-	+	+	+	-	+
K1461	2004	Massachusetts, USA	O4:K12	clinical (stool)	+	-	+	+	+	-	+
K5281	2007	Washington, USA	O4:K12	clinical (stool)	+	-	+	+	+	-	+
F5052	1997	Washington, USA	O4:K12	clinical (NK)	+	+	+	+	+	-	+
F7044	2000	Texas, USA	O4:K12	clinical (stool)	+	-	+	+	+	-	+
K5328	2007	Indiana, USA	O4:K12	clinical (stool)	+	-	+	+	+	-	+
F8170	2002	Hawaii, USA	O4:K12	clinical (stool)	+	-	+	+	+	-	+
K3752	2006	Washington, USA	O4:K12	clinical (stool)	+	-	+	+	+	-	+
K4358	2006	Oregon, USA	O4:K12	clinical (stool)	+	-	+	-	+	-	-
K1275	2004	Texas, USA	O3:K54	clinical (blood)	+	-	-	-	+	-	-
K3110	2006	Louisiana, USA	O4:K37	clinical (stool)	+	-	+	-	+	+	-
K4370	2006	Connecticut, USA	O4:K53	clinical (stool)	+	-	+	+	+	-	+
F7065	2000	California, USA	O4:K55	clinical (NK)	+	-	+	-	+	+	-
K4279	2006	Arizona, USA	O4:K63	clinical (stool)	+	-	+	-	+	-	-
K4250	2006	New York, USA	O4:K63	clinical (stool)	+	-	+	+	+	-	+
K3566	2006	Louisiana, USA	O4:K63	clinical (stool)	+	-	+	+	+	-	+
F7680	2001	California, USA	O4:K68	clinical (stool)	+	+	+	-	+	+	-
F7636	2001	California, USA	O4:K68	clinical (NK)	+	+	+	-	+	+	-
K0851	2004	Louisiana, USA	O1:Kuk	clinical (stool)	+	-	-	-	+	-	-
K0081	2003	Arizona, USA	O6:K18	clinical (stool)	+	-	+	+	+	-	+
K0850	2004	Louisiana, USA	O1:K25	clinical (NK)	+	-	-	-	+	-	-
F7635	2001	California, USA	O1:K25	clinical (NK)	+	+	+	-	+	+	-
F8701	2002	Minnesota, USA	O1:K25	clinical (stool)	+	+	+	-	+	+	-
F8837	2002	New Hampshire, USA	O1:K38	clinical (stool)	+	-	+	-	+	+	-
K1221	2004	Colorado, USA	O1:K56	clinical (stool)	+	-	+	+	+	-	+
K0909	2004	North Carolina, USA	O1:K56	clinical (stool)	+	-	+	+	+	-	+
K0923	2004	South Dakota, USA	O1:K56	clinical (stool)	+	-	+	+	+	-	+
K0456	2004	Massachusetts, USA	O1:Kuk	clinical (stool)	+	-	-	-	+	-	+
F9974	2003	New York, USA	O1:Kuk	clinical (stool)	+	-	-	-	+	-	-
K0071	2003	Tennessee, USA	O1:Kuk	clinical (stool)	+	+	+	-	+	-	-
K4763	2007	Virginia, USA	O5:Kuk	clinical (stool)	+	-	+	-	+	-	+

F6658	1999	Texas, USA	O5:Kuk	clinical (NK)	+	-	-	-	+	-	-
F8132	2002	Virginia, USA	O5:K17	clinical (wound)	+	-	-	-	+	-	-
F7979	2001	Minnesota, USA	O5:K17	clinical (stool)	+	+	-	-	+	-	-
K3528	2006	Louisiana, USA	O5:K56	clinical (stool)	+	-	+	-	+	-	+
F6179	1998	Connecticut, USA	O6:K18	clinical (NK)	+	-	-	-	+	-	-
F8937	2002	New York, USA	O4:K10	clinical (NK)	+	-	-	-	+	+	-
K4237	2006	New York, USA	O10:K56	clinical (stool)	+	-	-	-	+	-	+
K4376	2006	Maryland, USA	O8:K21	clinical (stool)	+	-	+	+	+	-	+
K4434	2006	Mississippi, USA	O6:K18	clinical (wound)	+	-	-	-	+	-	-
K0299	2004	Oregon, USA	O6:K18	clinical (stool)	+	-	+	+	+	-	+
F8190	2002	New York, USA	O6:K18	clinical (NK)	+	-	-	-	+	-	-
K3055	2006	Hawaii, USA	O6:K18	clinical (stool)	+	-	+	+	+	-	+
K4638	2007	New York, USA	O10:Kuk	clinical (stool)	+	-	-	-	+	-	+
K4981	2007	Georgia, USA	O1:Kuk	clinical (NK)	+	+	-	-	+	-	-
K5067	2007	South Dakota, USA	O1:K56	clinical (stool)	+	-	+	+	+	-	-
K5323G	2007	Virginia, USA	O5:K17	clinical (NK)	+	-	-	-	+	-	+
K5330	2007	Texas, USA	O5:Kuk	clinical (NK)	+	-	-	-	+	-	+
K4305	2006	Georgia, USA	O8:Kuk	clinical (stool)	+	-	+	-	+	-	+
K1000	2004	North Carolina, USA	O8:K20	clinical (NK)	+	-	-	-	+	-	-
K0845	2004	Hawaii, USA	O8:K21	clinical (stool)	+	+	+	-	+	+	-
F9869	2003	Nevada, USA	O9:K44	clinical (stool)	+	-	+	-	+	+	-
K4373	2006	Maryland, USA	O11:Kuk	clinical (stool)	+	-	+	+	+	-	+
K4381	2006	Maryland, USA	O11:Kuk	clinical (stool)	+	+	+	-	+	-	+
K5276	2007	New York, USA	O11:Kuk	clinical (stool)	+	-	+	+	+	-	+
K4374	2006	Maryland, USA	O11:K15	clinical (NK)	+	-	+	+	+	-	+
F8950	2002	Maryland, USA	O11:K43	clinical (stool)	+	-	-	-	+	+	-
22702	1998	Georgia, USA	O5:Kuk	environmental (sediment)	+	-	-	-	+	-	-
SG58	2006	Georgia, USA	ND	environmental (water)	+	-	-	-	+	-	-
SG151	2006	Georgia, USA	ND	environmental (water)	+	-	-	-	+	-	-
SG158	2006	Georgia, USA	ND	environmental (sediment)	+	-	-	-	+	-	-
SG174	2006	Georgia, USA	ND	environmental (water)	+	-	-	-	+	-	-
SG176	2006	Georgia, USA	O5:Kuk	environmental (water)	+	-	-	-	+	-	-
SG209	2006	Georgia, USA	O2:K28	environmental (sediment)	+	-	-	-	+	-	-
SG214	2006	Georgia, USA	O1:K9	environmental (water)	+	-	-	-	+	-	-
SG227	2006	Georgia, USA	ND	environmental (sediment)	+	-	-	-	+	-	-
SG244	2006	Georgia, USA	O6:Kuk	environmental (water)	+	-	-	-	+	-	-
SG247	2006	Georgia, USA	ND	environmental (water)	+	-	-	-	+	-	-
SG255	2006	Georgia, USA	ND	environmental (sediment)	+	-	-	-	+	-	-
SG257	2006	Georgia, USA	O8:Kuk	environmental (sediment)	+	-	-	+	+	-	+
SG258	2006	Georgia, USA	O1:Kuk	environmental (sediment)	+	-	-	-	+	-	-
SG259	2006	Georgia, USA	ND	environmental (sediment)	+	-	-	+	+	-	+
SG285	2006	Georgia, USA	O10:Kuk	environmental (sediment)	+	-	-	-	+	-	-
SG328	2006	Georgia, USA	ND	environmental (water)	+	-	-	-	+	-	-
SG354	2006	Georgia, USA	O2:K28	environmental (water)	+	-	-	-	+	-	-
SG364	2006	Georgia, USA	O1:Kuk	environmental (sediment)	+	-	-	-	+	-	-
SG372	2006	Georgia, USA	O5:Kuk	environmental (sediment)	+	-	-	-	+	-	-
SG398	2006	Georgia, USA	ND	environmental (sediment)	+	-	-	-	+	-	-
SG401	2006	Georgia, USA	O5:K61	environmental (sediment)	+	-	-	-	+	-	-
SG409	2006	Georgia, USA	O1:K33	environmental (sediment)	+	-	-	-	+	-	-
SG417	2006	Georgia, USA	O4:K67	environmental (sediment)	+	-	-	-	+	-	-
AF2	2006	Florida, USA	O4:K9	environmental (oyster)	+	-	-	-	+	-	-
AF8	2006	Florida, USA	ND	environmental (sediment)	+	-	-	-	+	-	-
AF12	2006	Florida, USA	ND	environmental (sediment)	+	-	-	-	+	-	-

AF34	2006	Florida, USA	O5:K37	environmental (sediment)	+	-	-	-	+	-	-
AF46	2006	Florida, USA	O11:Kuk	environmental (water)	+	-	-	-	+	-	-
AF47	2006	Florida, USA	ND	environmental (water)	+	-	-	-	+	-	-
AF56	2006	Florida, USA	O6:K37	environmental (sediment)	+	-	-	-	+	-	-
AF64	2006	Florida, USA	ND	environmental (sediment)	+	-	-	-	+	-	-
AF67	2006	Florida, USA	O5:K37	environmental (sediment)	+	-	-	-	+	-	-
AF91	2006	Florida, USA	O3:K6	environmental (sediment)	+	-	-	-	+	-	-
AF97	2006	Florida, USA	ND	environmental (water)	+	-	-	-	+	-	-
J-C2-6	1998	North Carolina, USA	ND	environmental (sediment)	+	-	-	-	+	-	-
J-C2-15	1998	North Carolina, USA	O1:Kuk	environmental (sediment)	+	-	-	-	+	-	-
J-C2-29	1998	North Carolina, USA	ND	environmental (sediment)	+	-	-	-	+	-	-
J-C2-34	1998	North Carolina, USA	O5:K19	environmental (sediment)	+	-	-	-	+	-	-
J-M1-23	1998	North Carolina, USA	O1:K32	environmental (sediment)	+	-	-	-	+	-	-
S-M2-2-B3	1998	North Carolina, USA	ND	environmental (sediment)	+	-	-	-	+	-	-
S-M2-3-B3	1998	North Carolina, USA	O5:K17	environmental (sediment)	+	-	-	-	+	-	-
7-SG1	2007	Georgia, USA	ND	environmental (sediment)	+	-	-	-	+	-	-
7-SG12	2007	Georgia, USA	ND	environmental (sediment)	+	-	-	-	+	-	-
7-SG38	2007	Georgia, USA	ND	environmental (sediment)	+	-	-	-	+	-	-
7-SG62	2007	Georgia, USA	ND	environmental (sediment)	+	-	-	-	+	-	-
7-SG81	2007	Georgia, USA	ND	environmental (sediment)	+	-	-	-	+	-	-
7-SG84	2007	Georgia, USA	ND	environmental (sediment)	+	-	-	-	+	-	-
7-SG127	2007	Georgia, USA	ND	environmental (sediment)	+	-	-	-	+	-	-
7-SG146	2007	Georgia, USA	ND	environmental (sediment)	+	-	-	-	+	-	-
7-SG159	2007	Georgia, USA	ND	environmental (sediment)	+	-	-	-	+	-	-
7-SG165	2007	Georgia, USA	ND	environmental (sediment)	+	-	-	-	+	-	-
7-SG167	2007	Georgia, USA	ND	environmental (sediment)	+	-	-	-	+	-	-
7-SG170	2007	Georgia, USA	ND	environmental (sediment)	+	-	-	-	+	-	-
7-SG180	2007	Georgia, USA	ND	environmental (sediment)	+	-	-	-	+	-	-
7-SG204	2007	Georgia, USA	ND	environmental (sediment)	+	-	-	-	+	-	-
7-SG206	2007	Georgia, USA	ND	environmental (sediment)	+	-	-	-	+	-	-

[a]ND is not determined

[b]NK indicates the sample type is not known; however, these isolates were associated with cases of human illness

Supplemental Table 2. LS-BSR clusters that are exclusive to the *V. parahaemolyticus* clinical or environmental genomes analyzed

		Clinical Isolate Genomes									Environmental Isolate Genomes				
**LS-BSR Cluster ID**	**Predicted Protein Function**	**K1275 (O3:K54)**	**K1461 (O4:K12)**	**10329 (O4:K12)**	**AN5034 (O4:K68)**	**AQ4037 (O3:K6)**	**K5030 (O3:K6)**	**AQ3810 (O3:K6)**	**RIMD2210633 (O3:K6)**	**Peru-466 (O3:K6)**	**SG176 (O5:Kuk)**	**AF91 (O3:K6)**	**22702 (O5:Kuk)**	**J-C2-34 (O5:K19)**	**BB22OP (O4:K8)**
**Gene Clusters Exclusive to Clinical Isolate Genomes**															
Cluster_10	hypothetical protein	-	-	-	+	-	-	-	-	-	-	-	-	-	-
Cluster_1000	hypothetical protein	-	-	-	+	-	+	-	+	-	-	-	-	-	-
Cluster_1008	hypothetical protein	-	-	-	-	-	-	+	-	-	-	-	-	-	-
Cluster_1012	hypothetical protein	-	-	-	+	-	-	-	+	+	-	-	-	-	-
Cluster_1017	Phage terminase large subunit	-	-	-	+	-	-	-	-	-	-	-	-	-	-
Cluster_1019	hypothetical protein	+	-	-	-	-	-	-	-	-	-	-	-	-	-
Cluster_1023	Putative phage protein	-	-	-	-	-	-	+	-	-	-	-	-	-	-
Cluster_1046	Rhamnulokinase (EC 2.7.1.5)	-	-	-	-	+	-	+	-	-	-	-	-	-	-
Cluster_1049	Phage terminase large subunit	-	-	+	-	-	-	-	-	-	-	-	-	-	-
Cluster_1075	FIG181673: hypothetical protein	-	+	+	-	+	-	-	-	-	-	-	-	-	-
Cluster_11	Cell division protein FtsK	-	-	-	-	-	+	-	-	-	-	-	-	-	-
Cluster_1109	putative ATP-binding protein	-	-	-	+	-	+	-	+	+	-	-	-	-	-
Cluster_114	conserved hypothetical protein	-	-	-	+	-	-	-	-	-	-	-	-	-	-
Cluster_1143	Zona occludens toxin	-	-	-	-	+	-	-	-	-	-	-	-	-	-
Cluster_1155	hypothetical protein	+	-	-	-	-	-	-	-	-	-	-	-	-	-
Cluster_1156	hypothetical protein	-	-	-	+	-	+	-	+	+	-	-	-	-	-
Cluster_123	Phage protein	-	-	-	-	+	-	-	-	-	-	-	-	-	-
Cluster_1256	DNA primase/helicase	-	-	-	+	-	-	-	-	-	-	-	-	-	-
Cluster_1266	FIG00640421: hypothetical protein	-	-	-	-	-	+	-	-	-	-	-	-	-	-
Cluster_1271	Signal transduction histidine kinase	+	-	-	-	-	-	-	-	-	-	-	-	-	-
Cluster_1272	hypothetical protein	+	-	-	-	-	-	-	-	-	-	-	-	-	-
Cluster_1273	hypothetical protein	-	-	-	-	-	-	+	-	-	-	-	-	-	-
Cluster_1283	hypothetical protein	-	-	-	+	-	+	-	+	+	-	-	-	-	-
Cluster_1359	PTS system, cellobiose-specific IIC component (EC 2.7.1.69)	-	-	-	-	+	-	+	-	-	-	-	-	-	-
Cluster_1370	hypothetical protein	-	-	-	-	+	-	-	-	-	-	-	-	-	-
Cluster_1378	putative chaperone	-	+	+	-	+	-	-	-	-	-	-	-	-	-
Cluster_139	RecD-like DNA helicase YrrC	+	-	-	-	-	-	-	-	-	-	-	-	-	-
Cluster_1391	hypothetical protein	-	-	-	+	-	+	-	+	+	-	-	-	-	-
Cluster_1401	COG0582: Integrase	+	-	-	-	-	-	-	-	-	-	-	-	-	-
Cluster_1416	hypothetical protein	-	+	+	-	-	-	-	-	-	-	-	-	-	-
Cluster_1419	hypothetical protein	-	-	+	-	-	-	-	-	-	-	-	-	-	-
Cluster_1427	hypothetical protein	+	-	-	-	-	-	-	-	-	-	-	-	-	-
Cluster_1432	DNA-cytosine methyltransferase (EC 2.1.1.37)	-	-	-	-	+	-	-	-	-	-	-	-	-	-
Cluster_1442	hypothetical protein	-	-	-	-	+	-	+	-	-	-	-	-	-	-
Cluster_1452	hypothetical protein	-	+	+	-	-	-	-	-	-	-	-	-	-	-
Cluster_1464	hypothetical protein	+	-	-	-	-	-	-	-	-	-	-	-	-	-
Cluster_147	hypothetical protein	-	-	-	+	-	+	-	+	+	-	-	-	-	-
Cluster_1486	L-rhamnose isomerase (EC 5.3.1.14)	-	-	-	-	+	-	+	-	-	-	-	-	-	-
Cluster_1488	hypothetical protein	-	-	-	+	-	-	-	-	-	-	-	-	-	-
Cluster_1499	Type I restriction-modification system, specificity subunit S (EC 3.1.21.3)	-	+	+	-	-	-	-	-	-	-	-	-	-	-
Cluster_15	FIG01203472: hypothetical protein	+	+	+	-	-	-	-	-	-	-	-	-	-	-
Cluster_151	Alfa-L-rhamnosidase (EC 3.2.1.40)	-	-	-	-	+	-	+	-	-	-	-	-	-	-
Cluster_1517	hypothetical protein	-	-	-	-	-	-	+	-	-	-	-	-	-	-
Cluster_1524	Mobile element protein	-	+	+	-	-	-	-	-	-	-	-	-	-	-
Cluster_1531	hypothetical protein	+	-	-	-	-	-	-	-	-	-	-	-	-	-
Cluster_1537	hypothetical protein	-	+	+	-	-	-	-	-	-	-	-	-	-	-
Cluster_1549	hypothetical protein	+	-	-	-	-	-	-	-	-	-	-	-	-	-
Cluster_156	hypothetical protein	-	-	-	+	-	+	-	+	+	-	-	-	-	-
Cluster_1598	hypothetical protein	-	-	-	+	-	+	-	+	+	-	-	-	-	-
Cluster_1622	Predicted membrane protein	+	-	-	-	-	-	-	-	-	-	-	-	-	-
Cluster_1639	Polymerase	-	+	+	-	-	-	-	-	-	-	-	-	-	-
Cluster_1650	hypothetical protein	+	-	-	-	-	-	-	-	-	-	-	-	-	-
Cluster_1652	hypothetical protein	-	+	+	-	-	-	-	-	-	-	-	-	-	-
Cluster_1660	Retron reverse transcriptase	-	-	+	-	-	-	-	-	-	-	-	-	-	-
Cluster_1670	FIG01205001: hypothetical protein	+	-	-	-	-	-	-	-	-	-	-	-	-	-
Cluster_1690	Mobile element protein	-	-	-	-	+	-	-	-	-	-	-	-	-	-
Cluster_1705	FIG01201591: hypothetical protein	-	-	-	+	-	-	-	-	-	-	-	-	-	-
Cluster_1716	hypothetical protein	-	-	+	-	-	-	-	-	-	-	-	-	-	-
Cluster_1723	Integrase	-	+	+	-	-	+	+	-	-	-	-	-	-	-
Cluster_1724	phage integrase family protein	+	-	-	-	-	-	-	-	-	-	-	-	-	-
Cluster_1751	Type I restriction-modification system, specificity subunit S (EC 3.1.21.3)	-	-	-	-	-	-	+	-	-	-	-	-	-	-
Cluster_1755	hypothetical protein	+	-	-	-	-	-	-	-	-	-	-	-	-	-
Cluster_1768	NADH-dependent butanol dehydrogenase A (EC 1.1.1.-)	-	-	-	-	+	-	+	-	-	-	-	-	-	-
Cluster_1780	hypothetical protein	-	+	+	-	-	-	-	-	-	-	-	-	-	-
Cluster_1782	hypothetical protein	+	-	-	-	-	-	-	-	-	-	-	-	-	-
Cluster_1786	hypothetical protein	-	-	-	+	-	-	-	-	-	-	-	-	-	-
Cluster_179	hypothetical protein	-	-	-	+	-	+	-	+	+	-	-	-	-	-
Cluster_1809	O-antigen polymerase	-	-	-	+	-	-	-	-	-	-	-	-	-	-
Cluster_1830	DNA polymerase I - 3'-5' exonuclease and polymerase domains-like	+	-	-	-	-	-	-	-	-	-	-	-	-	-
Cluster_1871	hypothetical protein	-	-	-	+	-	+	-	+	+	-	-	-	-	-
Cluster_1883	hypothetical protein	-	-	+	-	-	-	-	-	-	-	-	-	-	-
Cluster_1899	FIG221619: putative chaperone	-	+	+	-	+	-	-	-	-	-	-	-	-	-
Cluster_1925	hypothetical protein	-	-	-	-	-	-	+	-	-	-	-	-	-	-
Cluster_1926	capsular polysaccharide biosynthesis protein, putative	-	-	-	+	-	-	-	-	-	-	-	-	-	-
Cluster_193	FIG01199828: hypothetical protein	-	-	+	-	-	-	-	-	-	-	-	-	-	-
Cluster_1944	putative pore-forming cytotoxin integrase	-	-	-	+	-	+	-	+	+	-	-	-	-	-
Cluster_1956	hypothetical protein	-	-	-	-	+	-	-	-	-	-	-	-	-	-
Cluster_196	hypothetical protein	-	-	-	+	-	+	-	+	+	-	-	-	-	-
Cluster_1965	hypothetical protein	-	-	-	-	+	-	-	-	-	-	-	-	-	-
Cluster_197	Phage-related tail fiber protein	+	-	-	-	-	-	-	-	-	-	-	-	-	-
Cluster_1970	Tricorn protease N-terminal domain-containing protein	-	+	+	-	-	-	-	-	-	-	-	-	-	-
Cluster_1999	putative virulence protein	-	+	-	-	-	-	-	-	-	-	-	-	-	-
Cluster_20	Accessory colonization factor AcfD precursor	-	-	+	-	+	-	-	-	-	-	-	-	-	-
Cluster_200	hypothetical protein	-	-	+	-	-	-	-	-	-	-	-	-	-	-
Cluster_204	type I restriction enzyme R protein	-	-	-	+	-	+	-	+	+	-	-	-	-	-
Cluster_205	hypothetical protein	+	-	-	-	-	-	-	-	-	-	-	-	-	-
Cluster_2056	hypothetical protein	-	-	-	-	+	-	-	-	-	-	-	-	-	-
Cluster_2059	hypothetical protein	-	-	-	-	+	-	-	-	-	-	-	-	-	-

Cluster_2071	hypothetical protein	+	-	-	-	-	-	-	-	-	-	-	-	-	-
Cluster_2076	hypothetical protein	-	-	-	+	-	+	-	+	+	-	-	-	-	-
Cluster_2079	hypothetical protein	-	+	+	-	-	-	-	-	-	-	-	-	-	-
Cluster_2090	hypothetical protein	-	-	-	-	-	-	+	-	-	-	-	-	-	-
Cluster_2106	hypothetical protein	+	-	-	-	-	-	-	-	-	-	-	-	-	-
Cluster_2109	hypothetical protein	-	+	+	-	-	-	-	-	-	-	-	-	-	-
Cluster_2124	hypothetical protein	-	-	-	+	-	-	-	-	-	-	-	-	-	-
Cluster_2138	hypothetical protein	-	-	-	+	-	+	-	+	+	-	-	-	-	-
Cluster_2147	hypothetical protein	+	-	-	-	-	-	-	-	-	-	-	-	-	-
Cluster_2164	Phage portal protein	-	-	-	+	-	-	-	-	-	-	-	-	-	-
Cluster_2167	hypothetical protein	-	+	+	-	-	-	-	-	-	-	-	-	-	-
Cluster_2191	FIG01200737: hypothetical protein	-	+	+	-	+	-	-	-	-	-	-	-	-	-
Cluster_2197	Putative DNA-binding protein in cluster with Type I restriction-modification system	-	-	-	-	-	-	+	-	-	-	-	-	-	-
Cluster_2225	hypothetical protein	-	+	+	-	-	-	-	-	-	-	-	-	-	-
Cluster_2246	hypothetical protein	+	-	-	-	-	-	-	-	-	-	-	-	-	-
Cluster_2249	hypothetical protein	-	-	-	-	+	-	-	-	-	-	-	-	-	-
Cluster_2273	putative IS1328 transposase	-	-	-	+	-	+	-	+	+	-	-	-	-	-
Cluster_2274	hypothetical protein	-	+	+	-	-	-	-	-	-	-	-	-	-	-
Cluster_2280	Mrr restriction protein-related	+	-	-	-	-	-	-	-	-	-	-	-	-	-
Cluster_2282	Membrane fusion component of tripartite multidrug resistance system	-	+	+	-	-	-	-	-	-	-	-	-	-	-
Cluster_2285	FIG01203179: hypothetical protein	-	+	+	-	-	-	-	-	-	-	-	-	-	-
Cluster_2304	hypothetical protein	-	-	-	+	-	+	-	+	+	-	-	-	-	-
Cluster_2308	hypothetical protein	-	-	-	+	-	-	-	-	-	-	-	-	-	-
Cluster_2310	hypothetical protein	-	-	-	-	+	-	-	-	-	-	-	-	-	-
Cluster_2321	hypothetical protein	-	-	-	+	-	-	-	-	-	-	-	-	-	-
Cluster_2324	regulatory protein, LysR:LysR, substrate-binding	+	-	-	-	-	-	-	-	-	-	-	-	-	-
Cluster_234	conserved hypothetical protein	+	-	-	-	-	-	-	-	-	-	-	-	-	-
Cluster_2351	Glycosyl transferase, group 2 family protein	-	-	-	+	-	-	-	-	-	-	-	-	-	-
Cluster_2355	2-oxoglutarate oxidoreductase, beta subunit (EC 1.2.7.3)	-	+	+	-	-	-	-	-	-	-	-	-	-	-
Cluster_2360	Rossmann fold nucleotide-binding protein Smf possibly involved in DNA uptake	-	-	-	-	-	-	+	-	-	-	-	-	-	-
Cluster_2366	putative inner membrane protein	-	-	-	+	-	+	-	+	+	-	-	-	-	-
Cluster_2369	TniB NTP-binding protein	-	+	+	-	-	-	-	-	-	-	-	-	-	-
Cluster_2413	Mobile element protein	-	-	-	-	+	-	-	-	-	-	-	-	-	-
Cluster_2443	Mobile element protein	-	+	+	-	-	-	-	-	-	-	-	-	-	-
Cluster_245	hypothetical protein	-	-	-	+	-	+	-	+	+	-	-	-	-	-
Cluster_2476	FIG221619: putative chaperone	-	-	-	-	+	-	-	-	-	-	-	-	-	-
Cluster_2483	Transcriptional regulator of rhamnose utilization, AraC family	-	-	-	-	+	-	+	-	-	-	-	-	-	-
Cluster_2489	Dipeptide transport system permease protein DppB (TC 3.A.1.5.2)	-	+	+	-	+	-	-	-	-	-	-	-	-	-
Cluster_2528	hypothetical protein	-	-	-	-	-	+	-	+	+	-	-	-	-	-
Cluster_254	hypothetical protein	-	+	-	-	-	-	-	-	-	-	-	-	-	-
Cluster_2546	Primosomal protein I	-	+	+	-	-	-	-	-	-	-	-	-	-	-
Cluster_2565	hypothetical protein	-	-	-	-	+	-	-	-	-	-	-	-	-	-
Cluster_2572	hypothetical protein	-	-	+	-	-	-	-	-	-	-	-	-	-	-
Cluster_2573	hypothetical protein	+	-	-	-	-	-	-	-	-	-	-	-	-	-
Cluster_2595	FIG01201305: hypothetical protein	-	-	-	-	+	-	+	-	-	-	-	-	-	-
Cluster_2602	hypothetical protein	-	-	-	-	-	-	+	-	-	-	-	-	-	-
Cluster_2603	hypothetical protein	+	-	-	-	-	-	-	-	-	-	-	-	-	-
Cluster_2616	hypothetical protein	-	-	+	-	-	-	+	-	-	-	-	-	-	-
Cluster_2647	hypothetical protein	+	-	-	-	-	-	-	-	-	-	-	-	-	-
Cluster_2659	FIG01205951: hypothetical protein	-	-	-	-	-	-	+	-	-	-	-	-	-	-
Cluster_2660	hypothetical protein	-	+	+	-	-	-	-	-	-	-	-	-	-	-
Cluster_2668	hypothetical protein	-	+	+	-	-	-	-	-	-	-	-	-	-	-
Cluster_2677	hypothetical protein	-	-	-	-	-	+	-	-	-	-	-	-	-	-
Cluster_2680	hypothetical protein	-	-	-	-	+	-	-	-	-	-	-	-	-	-
Cluster_269	hypothetical protein	+	-	-	-	-	-	-	-	-	-	-	-	-	-
Cluster_272	hypothetical protein	-	-	-	+	-	+	-	+	+	-	-	-	-	-
Cluster_273	Type I restriction-modification system, restriction subunit R (EC 3.1.21.3)	-	-	-	-	-	-	+	-	-	-	-	-	-	-
Cluster_2747	hypothetical protein	+	-	-	-	-	-	-	-	-	-	-	-	-	-
Cluster_2763	hypothetical protein	-	+	+	-	+	-	-	-	-	-	-	-	-	-
Cluster_2764	hypothetical protein	-	-	-	-	-	-	+	-	-	-	-	-	-	-
Cluster_2775	Permease of the drug/metabolite transporter (DMT) superfamily	-	+	+	-	+	-	-	-	-	-	-	-	-	-
Cluster_279	hypothetical protein	-	-	-	+	-	-	-	-	-	-	-	-	-	-
Cluster_2801	hypothetical protein	-	+	+	-	-	-	-	-	-	-	-	-	-	-
Cluster_2802	UDP-glucose 4-epimerase (EC 5.1.3.2)	-	+	+	-	-	-	-	-	-	-	-	-	-	-
Cluster_2803	hypothetical protein	-	-	-	-	-	-	+	-	-	-	-	-	-	-
Cluster_2805	HNH endonuclease	-	-	-	-	+	-	-	-	-	-	-	-	-	-
Cluster_2819	hypothetical protein	-	+	+	-	-	-	-	-	-	-	-	-	-	-
Cluster_2825	hypothetical protein	-	-	-	+	-	-	-	-	-	-	-	-	-	-
Cluster_2838	hypothetical protein	+	-	-	-	+	-	+	-	-	-	-	-	-	-
Cluster_2850	DNA sulfur modification protein DndB	-	-	-	-	-	+	-	-	-	-	-	-	-	-
Cluster_2895	hypothetical protein	-	+	+	-	-	-	-	-	-	-	-	-	-	-
Cluster_2908	hypothetical protein	-	-	-	-	+	-	-	-	-	-	-	-	-	-
Cluster_2909	putative transcriptional regulator	+	-	-	-	-	-	-	-	-	-	-	-	-	-
Cluster_2925	sensory box protein	+	-	-	-	-	-	-	-	-	-	-	-	-	-
Cluster_2926	hypothetical protein	-	-	-	-	+	-	-	-	-	-	-	-	-	-
Cluster_2936	hypothetical protein	-	+	-	-	-	-	-	-	-	-	-	-	-	-
Cluster_2947	hypothetical protein	-	+	+	-	-	-	-	-	-	-	-	-	-	-
Cluster_2969	oxidoreductase, aldo/keto reductase family	-	-	-	-	+	-	+	-	-	-	-	-	-	-
Cluster_2992	Alpha-1,2-fucosyltransferase	-	+	+	-	-	-	-	-	-	-	-	-	-	-
Cluster_2994	DNA-damage-inducible protein D	-	+	+	-	-	-	-	-	-	-	-	-	-	-
Cluster_2996	hypothetical protein	-	+	+	-	-	-	-	-	-	-	-	-	-	-
Cluster_2997	transferase	+	-	-	-	-	-	-	-	-	-	-	-	-	-
Cluster_3021	hypothetical protein	+	-	-	-	-	-	-	-	-	-	-	-	-	-
Cluster_3023	FIG01201418: hypothetical protein	-	+	+	-	-	+	+	-	-	-	-	-	-	-
Cluster_3025	hypothetical protein	-	+	+	-	-	-	-	-	-	-	-	-	-	-
Cluster_3029	hypothetical protein	-	-	+	-	-	-	-	-	-	-	-	-	-	-
Cluster_3031	Protein involved in stability of MscS mechanosensitive channel	-	-	-	-	-	+	-	-	-	-	-	-	-	-
Cluster_3032	Mobile element protein	-	-	-	-	-	+	-	-	-	-	-	-	-	-
Cluster_304	hypothetical protein	-	+	+	-	-	-	-	-	-	-	-	-	-	-
Cluster_3059	hypothetical protein	+	-	-	-	-	-	-	-	-	-	-	-	-	-
Cluster_3060	transcriptional regulator, AraC family protein	-	+	+	-	+	-	-	-	-	-	-	-	-	-
Cluster_3061	hypothetical protein	-	-	-	+	-	-	-	-	-	-	-	-	-	-
Cluster_3066	COG3740: Phage head maturation protease	-	-	-	+	-	-	-	-	-	-	-	-	-	-

Cluster_3075	hypothetical protein	+	-	-	+	+	+	+	-	+	-	-	-	-	-
Cluster_3083	Urease accessory protein UreD	-	+	+	-	+	-	-	-	-	-	-	-	-	-
Cluster_3091	phage exonuclease, putative	-	-	-	+	-	-	-	-	-	-	-	-	-	-
Cluster_3094	Transcriptional regulator, LysR family	-	+	+	-	-	-	-	-	-	-	-	-	-	-
Cluster_3103	hypothetical protein	-	-	-	-	-	-	+	-	-	-	-	-	-	-
Cluster_3105	outer membrane protein	+	+	+	-	-	-	-	-	-	-	-	-	-	-
Cluster_3117	L-rhamnose operon regulatory protein RhaS	-	-	-	-	+	-	+	-	-	-	-	-	-	-
Cluster_3129	Chromosome (plasmid) partitioning protein ParA / Sporulation initiation inhibitor protein Soj	-	-	+	-	-	-	-	-	-	-	-	-	-	-
Cluster_3157	putative protein Ymh	-	-	-	+	-	+	-	+	+	-	-	-	-	-
Cluster_3159	hypothetical protein	+	-	-	-	-	-	-	-	-	-	-	-	-	-
Cluster_3166	Mobile element protein	-	-	-	-	+	-	+	-	-	-	-	-	-	-
Cluster_3169	hypothetical protein	+	-	-	-	-	-	-	-	-	-	-	-	-	-
Cluster_3178	hypothetical protein	+	-	-	-	-	-	-	-	-	-	-	-	-	-
Cluster_3190	hypothetical protein	-	+	+	-	-	-	-	-	-	-	-	-	-	-
Cluster_3202	hypothetical protein	+	-	-	-	-	-	-	-	-	-	-	-	-	-
Cluster_3204	hypothetical protein	+	-	-	-	-	-	-	-	-	-	-	-	-	-
Cluster_3205	hypothetical protein	+	-	-	-	-	-	-	-	-	-	-	-	-	-
Cluster_321	hypothetical protein	-	-	-	+	-	-	-	-	-	-	-	-	-	-
Cluster_3219	hypothetical protein	-	+	+	-	-	-	-	-	-	-	-	-	-	-
Cluster_3226	putative transcriptional regulator ToxR	-	+	+	-	-	-	-	-	-	-	-	-	-	-
Cluster_3227	Glycosyltransferase (EC 2.4.1.-)	-	-	-	+	-	-	-	-	-	-	-	-	-	-
Cluster_3244	hypothetical protein	-	-	+	-	-	-	-	-	-	-	-	-	-	-
Cluster_3251	hypothetical protein	-	+	+	-	-	-	-	-	-	-	-	-	-	-
Cluster_3253	hypothetical protein	+	-	-	-	-	-	-	-	-	-	-	-	-	-
Cluster_3254	hypothetical protein	-	-	-	-	+	-	-	-	-	-	-	-	-	-
Cluster_3259	FIG01200803: hypothetical protein	-	-	-	+	-	-	-	-	-	-	-	-	-	-
Cluster_3265	hypothetical protein	-	-	-	+	-	-	-	-	-	-	-	-	-	-
Cluster_3266	hypothetical protein	-	-	-	-	-	+	-	-	-	-	-	-	-	-
Cluster_327	putative phage-related protein	-	-	-	+	-	+	-	+	+	-	-	-	-	-
Cluster_3275	Dipeptide transport system permease protein DppC (TC 3.A.1.5.2)	-	+	+	-	+	-	-	-	-	-	-	-	-	-
Cluster_3278	no significant match	-	-	-	+	-	-	-	-	-	-	-	-	-	-
Cluster_3280	hypothetical protein	-	-	-	-	+	-	-	-	-	-	-	-	-	-
Cluster_3281	Rhamnulose-1-phosphate aldolase (EC 4.1.2.19)	-	-	-	-	+	-	+	-	-	-	-	-	-	-
Cluster_3290	hypothetical protein	-	-	-	-	-	-	+	-	-	-	-	-	-	-
Cluster_3296	hypothetical protein	-	-	-	-	-	-	+	-	-	-	-	-	-	-
Cluster_3305	hypothetical protein	-	+	+	-	-	-	-	-	-	-	-	-	-	-
Cluster_3306	Tricorn protease N-terminal domain-containing protein	+	+	+	-	-	-	-	-	-	-	-	-	-	-
Cluster_3307	FIG01206276: hypothetical protein	-	-	-	-	-	+	-	-	-	-	-	-	-	-
Cluster_331	Hypothetical SecA-related protein	-	-	-	-	+	-	-	-	-	-	-	-	-	-
Cluster_3324	putative cytoplasmic protein	+	-	-	-	-	-	-	-	-	-	-	-	-	-
Cluster_3330	hypothetical protein	-	+	+	-	-	-	-	-	-	-	-	-	-	-
Cluster_3331	FIG01200278: hypothetical protein	-	+	-	-	+	-	-	-	-	-	-	-	-	-
Cluster_3342	putative transcriptional regulator ToxR	-	+	+	-	+	-	-	-	-	-	-	-	-	-
Cluster_3360	hypothetical protein	-	-	-	+	-	+	+	+	+	-	-	-	-	-
Cluster_3372	FIG01204702: hypothetical protein	-	-	-	+	+	+	+	-	+	-	-	-	-	-
Cluster_3380	hypothetical protein	+	-	-	-	-	-	-	-	-	-	-	-	-	-
Cluster_3383	hypothetical protein	-	-	-	-	-	-	+	-	-	-	-	-	-	-
Cluster_3396	ORF25	-	-	+	-	-	-	-	-	-	-	-	-	-	-
Cluster_3408	hypothetical protein	+	-	-	-	-	-	-	-	-	-	-	-	-	-
Cluster_3449	hypothetical protein	-	-	-	-	-	-	+	-	-	-	-	-	-	-
Cluster_3450	hypothetical protein	-	-	-	+	-	-	-	-	-	-	-	-	-	-
Cluster_3468	hypothetical protein	+	-	-	-	-	-	-	-	-	-	-	-	-	-
Cluster_3486	hypothetical protein	+	-	-	-	-	-	-	-	-	-	-	-	-	-
Cluster_3517	hypothetical protein	-	-	-	+	-	-	-	-	-	-	-	-	-	-
Cluster_3518	hypothetical protein	+	-	-	-	-	-	-	-	-	-	-	-	-	-
Cluster_3524	FIG01202770: hypothetical protein	-	+	+	-	+	-	-	-	-	-	-	-	-	-
Cluster_3525	hypothetical protein	+	+	+	-	-	-	-	-	-	-	-	-	-	-
Cluster_3532	Osmosensitive K+ channel histidine kinase KdpD (EC 2.7.3.-)	-	-	-	-	-	-	+	-	-	-	-	-	-	-
Cluster_3537	IS, phage, Tn; Transposon-related functions	-	+	+	-	-	-	-	-	-	-	-	-	-	-
Cluster_3544	Putative predicted metal-dependent hydrolase	-	+	+	-	-	-	-	-	-	-	-	-	-	-
Cluster_3555	HTH-type transcriptional regulator gadW	+	-	-	+	+	+	+	-	+	-	-	-	-	-
Cluster_3565	hypothetical protein	-	-	-	+	-	-	-	-	-	-	-	-	-	-
Cluster_3579	Alkyl sulfatase (EC 3.1.6.-)	-	+	+	-	-	-	-	-	-	-	-	-	-	-
Cluster_358	hypothetical protein	+	-	-	-	-	-	-	-	-	-	-	-	-	-
Cluster_3597	Nucleoside-diphosphate-sugar pyrophosphorylase involved in lipopolysaccharide biosynthesis/translation initiation factor 2B	-	-	-	+	-	-	-	-	-	-	-	-	-	-
Cluster_3607	hypothetical protein	-	+	+	-	-	-	-	-	-	-	-	-	-	-
Cluster_3612	hypothetical protein	+	-	-	-	-	-	-	-	-	-	-	-	-	-
Cluster_3614	Nucleoside-diphosphate-sugar pyrophosphorylase involved in lipopolysaccharide biosynthesis/translation initiation factor 2B	+	-	-	-	-	-	-	-	-	-	-	-	-	-
Cluster_3632	hypothetical protein	+	-	-	-	-	-	-	-	-	-	-	-	-	-
Cluster_3644	hypothetical protein	-	-	-	+	-	+	-	+	+	-	-	-	-	-
Cluster_3680	FIG01205951: hypothetical protein	-	-	-	-	-	-	+	-	-	-	-	-	-	-
Cluster_3689	Alkylated DNA repair protein AlkB	-	-	+	-	-	-	-	-	-	-	-	-	-	-
Cluster_3697	Kappa-fimbriae chaperone protein	-	+	-	-	-	-	-	-	-	-	-	-	-	-
Cluster_3698	hypothetical protein	-	-	-	-	+	-	-	-	-	-	-	-	-	-
Cluster_3699	Nucleoside-diphosphate-sugar pyrophosphorylase involved in lipopolysaccharide biosynthesis/translation initiation factor 2B	-	-	+	-	-	-	-	-	-	-	-	-	-	-
Cluster_3702	hypothetical protein	-	+	+	-	-	-	-	-	-	-	-	-	-	-
Cluster_3714	FIG061186: hypothetical protein	-	+	+	-	+	-	-	-	-	-	-	-	-	-
Cluster_3727	hypothetical protein	-	-	+	-	-	-	-	-	-	-	-	-	-	-
Cluster_3736	replication P family protein	+	-	-	-	-	-	-	-	-	-	-	-	-	-
Cluster_3752	hypothetical protein	-	+	-	-	-	-	-	-	-	-	-	-	-	-
Cluster_3768	hypothetical protein	-	-	+	-	-	-	-	-	-	-	-	-	-	-
Cluster_3775	FIG01203179: hypothetical protein	+	+	+	-	-	-	-	-	-	-	-	-	-	-
Cluster_3790	hypothetical protein	+	-	-	-	-	-	-	-	-	-	-	-	-	-
Cluster_3792	hypothetical protein	-	+	+	-	-	-	+	-	-	-	-	-	-	-
Cluster_3806	Two-component system response regulator QseB	+	-	-	-	-	-	-	-	-	-	-	-	-	-
Cluster_3822	hypothetical protein	-	+	+	-	-	-	-	-	-	-	-	-	-	-
Cluster_3829	hypothetical protein	-	-	+	-	-	-	-	-	-	-	-	-	-	-
Cluster_3835	hypothetical protein	-	-	-	-	-	-	+	-	-	-	-	-	-	-
Cluster_3840	hypothetical protein	+	-	-	-	-	-	-	-	-	-	-	-	-	-
Cluster_3844	hypothetical protein	-	-	-	-	-	-	+	-	-	-	-	-	-	-
Cluster_3846	Oligopeptide transport ATP-binding protein OppD (TC 3.A.1.5.1)	-	+	+	-	+	-	-	-	-	-	-	-	-	-
Cluster_3854	hypothetical protein	-	+	+	-	-	-	+	-	-	-	-	-	-	-
Cluster_3867	hypothetical protein	+	-	-	-	-	-	-	-	-	-	-	-	-	-

Cluster_3869	Phage repressor protein C2	-	+	+	-	-	-	-	-	-	-	-	-	-	-
Cluster_3887	hypothetical protein	-	+	+	-	-	-	-	-	-	-	-	-	-	-
Cluster_3888	FIG01202005: hypothetical protein	+	-	-	-	-	-	-	-	-	-	-	-	-	-
Cluster_390	DNA polymerase	-	-	-	+	-	-	-	-	-	-	-	-	-	-
Cluster_3908	Phage protein	-	-	-	-	+	-	-	-	-	-	-	-	-	-
Cluster_3915	hypothetical protein	-	-	-	+	-	+	-	+	+	-	-	-	-	-
Cluster_3925	hypothetical protein	+	-	-	-	-	-	-	-	-	-	-	-	-	-
Cluster_3933	hypothetical protein	-	-	-	+	-	+	-	+	+	-	-	-	-	-
Cluster_3934	hypothetical protein	-	-	-	+	-	+	-	+	+	-	-	-	-	-
Cluster_3945	hypothetical protein	-	-	-	-	-	-	+	-	-	-	-	-	-	-
Cluster_3946	ThiJ/PfpI family protein	+	-	-	-	-	-	-	-	-	-	-	-	-	-
Cluster_3968	hypothetical protein	-	+	+	-	-	-	-	-	-	-	-	-	-	-
Cluster_3979	Urease accessory protein UreF	-	+	+	-	+	-	-	-	-	-	-	-	-	-
Cluster_3981	hypothetical protein	-	-	-	-	-	-	+	-	-	-	-	-	-	-
Cluster_3983	hypothetical protein	+	-	-	-	-	-	-	-	-	-	-	-	-	-
Cluster_3992	hypothetical protein	-	+	+	-	-	-	-	-	-	-	-	-	-	-
Cluster_3994	hypothetical protein	-	-	-	-	+	-	-	-	-	-	-	-	-	-
Cluster_3999	hypothetical protein	-	-	+	-	-	-	-	-	-	-	-	-	-	-
Cluster_4003	hypothetical protein	-	-	-	-	+	-	-	-	-	-	-	-	-	-
Cluster_4004	Transcriptional regulator VpsT	-	+	-	-	-	-	-	-	-	-	-	-	-	-
Cluster_401	FIG01200972: hypothetical protein	-	-	-	-	-	+	-	-	-	-	-	-	-	-
Cluster_4022	putative integral membrane protein	+	+	+	-	-	-	-	-	-	-	-	-	-	-
Cluster_4025	hypothetical protein	+	-	-	-	-	-	-	-	-	-	-	-	-	-
Cluster_4034	FIG01201940: hypothetical protein	-	-	-	+	-	-	-	-	-	-	-	-	-	-
Cluster_4041	putative integral membrane protein	+	+	+	-	-	-	-	-	-	-	-	-	-	-
Cluster_4042	hypothetical protein	+	-	-	-	-	-	-	-	-	-	-	-	-	-
Cluster_4048	hypothetical protein	-	-	-	-	-	-	+	-	-	-	-	-	-	-
Cluster_4058	hypothetical protein	-	-	-	-	+	-	-	-	-	-	-	-	-	-
Cluster_4076	Phage protein	-	+	+	-	-	-	-	-	-	-	-	-	-	-
Cluster_4095	hypothetical protein	-	-	-	+	-	+	-	+	+	-	-	-	-	-
Cluster_4096	hypothetical protein	-	-	-	+	-	-	-	-	-	-	-	-	-	-
Cluster_4098	FIG01202149: hypothetical protein	-	+	+	-	-	-	-	-	-	-	-	-	-	-
Cluster_4104	hypothetical protein	-	-	-	+	-	-	-	-	-	-	-	-	-	-
Cluster_4127	hypothetical protein	-	-	-	+	-	+	-	+	+	-	-	-	-	-
Cluster_4129	hypothetical protein	-	-	-	+	-	+	-	+	+	-	-	-	-	-
Cluster_4136	Putative exonuclease	-	-	+	-	-	-	-	-	-	-	-	-	-	-
Cluster_4137	Urease accessory protein UreG	-	+	+	-	+	-	-	-	-	-	-	-	-	-
Cluster_4145	hypothetical protein	-	-	-	+	-	+	-	+	+	-	-	-	-	-
Cluster_4149	putative threonine efflux protein	-	-	-	+	-	+	-	+	+	-	-	-	-	-
Cluster_415	hypothetical protein	-	-	-	-	-	-	+	-	-	-	-	-	-	-
Cluster_4161	hypothetical protein	+	-	-	-	-	-	-	-	-	-	-	-	-	-
Cluster_4162	hypothetical protein	-	-	-	-	-	-	+	-	-	-	-	-	-	-
Cluster_4173	hypothetical protein	-	-	-	-	-	+	-	-	-	-	-	-	-	-
Cluster_4178	hypothetical protein	-	-	-	+	-	-	-	-	-	-	-	-	-	-
Cluster_4198	hypothetical protein	-	-	-	+	-	+	-	+	+	-	-	-	-	-
Cluster_4214	Dipeptide transport ATP-binding protein DppF (TC 3.A.1.5.2)	-	+	+	-	+	-	-	-	-	-	-	-	-	-
Cluster_4221	hypothetical protein	-	+	-	-	-	-	-	-	-	-	-	-	-	-
Cluster_4230	hypothetical protein	-	+	+	-	-	-	-	-	-	-	-	-	-	-
Cluster_424	DNA sulfur modification protein DndD	-	-	-	-	-	+	-	-	-	-	-	-	-	-
Cluster_4243	hypothetical protein	-	-	-	+	-	-	-	-	-	-	-	-	-	-
Cluster_4260	Transcriptional regulator, TetR family	-	+	+	-	-	-	-	-	-	-	-	-	-	-
Cluster_4264	Alkyl sulfatase (EC 3.1.6.-)	-	+	+	-	-	-	-	-	-	-	-	-	-	-
Cluster_4279	FIG01201961: hypothetical protein	-	+	+	-	-	-	+	-	-	-	-	-	-	-
Cluster_4304	hypothetical protein	-	-	-	-	+	-	-	-	-	-	-	-	-	-
Cluster_4308	NAD(P)H oxidoreductase YRKL (EC 1.6.99.-)	-	-	-	-	+	-	-	-	-	-	-	-	-	-
Cluster_4318	Phosphohydrolase (MutT/nudix family protein)	-	-	+	-	-	-	-	-	-	-	-	-	-	-
Cluster_4321	hypothetical protein	-	-	+	-	-	-	-	-	-	-	-	-	-	-
Cluster_4323	hypothetical protein	-	+	+	-	-	-	-	-	-	-	-	-	-	-
Cluster_4380	ThiJ/PfpI family protein	+	-	-	-	-	-	-	-	-	-	-	-	-	-
Cluster_4383	partitioning protein, putative	-	-	-	-	+	-	-	-	-	-	-	-	-	-
Cluster_4391	hypothetical protein	-	+	+	-	-	-	-	-	-	-	-	-	-	-
Cluster_4395	ORF5	-	-	-	+	-	-	-	-	-	-	-	-	-	-
Cluster_44	hypothetical protein	-	+	-	-	-	-	-	-	-	-	-	-	-	-
Cluster_4405	hypothetical protein	+	-	-	-	-	-	-	-	-	-	-	-	-	-
Cluster_4412	FIG01203606: hypothetical protein	-	-	-	+	-	-	-	-	-	-	-	-	-	-
Cluster_4413	phospholipase/carboxylesterase	-	+	+	-	-	-	-	-	-	-	-	-	-	-
Cluster_442	hypothetical protein	-	+	+	-	-	-	-	-	-	-	-	-	-	-
Cluster_4420	ORF5	-	-	-	+	-	-	-	-	-	-	-	-	-	-
Cluster_4436	hypothetical protein	+	-	-	-	-	-	-	-	-	-	-	-	-	-
Cluster_4438	Outer membrane protein	+	-	-	-	-	-	-	-	-	-	-	-	-	-
Cluster_4450	hypothetical protein	-	+	-	-	-	-	-	-	-	-	-	-	-	-
Cluster_4453	hypothetical protein	-	+	+	-	-	-	-	-	-	-	-	-	-	-
Cluster_4455	hypothetical protein	-	-	-	+	-	-	-	-	-	-	-	-	-	-
Cluster_4468	hypothetical protein	-	+	+	-	-	-	-	-	-	-	-	-	-	-
Cluster_4493	hypothetical protein	-	-	-	-	-	-	+	-	-	-	-	-	-	-
Cluster_4495	FIG01201961: hypothetical protein	-	-	-	-	-	+	-	-	-	-	-	-	-	-
Cluster_4506	Acetyltransferase	-	+	+	-	-	-	-	-	-	-	-	-	-	-
Cluster_4508	hypothetical protein	-	-	+	-	-	-	-	-	-	-	-	-	-	-
Cluster_4509	hypothetical protein	-	+	+	-	-	-	-	-	-	-	-	-	-	-
Cluster_451	IncF plasmid conjugative transfer protein TraD	+	-	-	-	-	-	-	-	-	-	-	-	-	-
Cluster_4518	FIG079833: putative dimethyladenosine transferase	-	+	+	-	+	-	-	-	-	-	-	-	-	-
Cluster_4520	hypothetical protein	-	+	+	-	-	-	-	-	-	-	-	-	-	-
Cluster_4542	orf4	+	-	-	-	-	-	-	-	-	-	-	-	-	-
Cluster_4550	hypothetical protein	-	+	+	-	-	-	-	-	-	-	-	-	-	-
Cluster_4567	hypothetical protein	-	+	-	-	-	-	-	-	-	-	-	-	-	-
Cluster_4587	hypothetical protein	-	+	+	-	-	-	-	-	-	-	-	-	-	-
Cluster_4590	hypothetical protein	+	-	-	-	-	-	-	-	-	-	-	-	-	-
Cluster_4609	hypothetical protein	-	-	-	-	-	-	+	-	-	-	-	-	-	-
Cluster_4615	hypothetical protein	-	-	-	+	-	-	-	-	-	-	-	-	-	-
Cluster_4637	hypothetical protein	+	-	-	-	-	-	-	-	-	-	-	-	-	-
Cluster_4640	hypothetical protein	-	-	-	+	-	+	-	+	+	-	-	-	-	-
Cluster_4653	COG0030: Dimethyladenosine transferase (rRNA methylation)	+	-	-	-	-	-	-	-	-	-	-	-	-	-
Cluster_4673	hypothetical protein	-	+	+	-	-	-	-	-	-	-	-	-	-	-

Cluster_4674	hypothetical protein	-	-	-	+	-	-	-	-	-	-	-	-	-	-
Cluster_4678	hypothetical protein	-	-	+	-	-	-	-	-	-	-	-	-	-	-
Cluster_4681	putative DNA-binding protein	-	+	-	-	-	-	-	-	-	-	-	-	-	-
Cluster_4687	hypothetical protein	-	+	+	-	-	-	-	-	-	-	-	-	-	-
Cluster_4711	hypothetical protein	+	-	-	-	-	-	-	-	-	-	-	-	-	-
Cluster_4714	Isochorismatase (EC 3.3.2.1)	-	-	-	-	-	-	+	-	-	-	-	-	-	-
Cluster_4733	hypothetical protein	-	-	-	+	-	-	-	-	-	-	-	-	-	-
Cluster_4735	hypothetical protein	-	+	-	-	-	-	-	-	-	-	-	-	-	-
Cluster_4746	hypothetical protein	-	+	+	-	-	-	-	-	-	-	-	-	-	-
Cluster_4768	Acetyltransferase	-	-	-	-	-	-	+	-	-	-	-	-	-	-
Cluster_4772	hypothetical protein	-	-	-	-	+	-	-	-	-	-	-	-	-	-
Cluster_4776	Butyryltransferase	-	-	-	+	-	-	-	-	-	-	-	-	-	-
Cluster_4778	hypothetical protein	+	-	-	-	-	-	-	-	-	-	-	-	-	-
Cluster_4781	hypothetical protein	-	-	-	-	+	-	-	-	-	-	-	-	-	-
Cluster_4802	hypothetical protein	-	-	-	+	-	-	-	-	-	-	-	-	-	-
Cluster_4823	hypothetical protein	+	-	-	-	-	-	-	-	-	-	-	-	-	-
Cluster_4833	hypothetical protein	-	-	-	-	+	-	-	-	-	-	-	-	-	-
Cluster_4846	hypothetical protein	-	+	+	-	-	-	-	-	-	-	-	-	-	-
Cluster_4850	FIG01206151: hypothetical protein	+	-	-	-	-	-	-	-	-	-	-	-	-	-
Cluster_4853	FIG01202829: hypothetical protein	-	-	-	-	-	-	+	-	-	-	-	-	-	-
Cluster_4880	FIG01202829: hypothetical protein	-	-	-	-	-	+	-	-	-	-	-	-	-	-
Cluster_4882	hypothetical protein	+	-	-	-	-	-	-	-	-	-	-	-	-	-
Cluster_4896	FIG01203269: hypothetical protein	-	-	-	+	-	-	-	-	-	-	-	-	-	-
Cluster_4927	hypothetical protein	-	-	-	+	-	-	-	-	-	-	-	-	-	-
Cluster_4929	hypothetical protein	-	+	+	-	-	-	-	-	-	-	-	-	-	-
Cluster_4948	hypothetical protein	-	+	+	-	-	-	-	-	-	-	-	-	-	-
Cluster_4970	hypothetical protein	-	+	+	-	-	-	-	-	-	-	-	-	-	-
Cluster_4971	hypothetical protein	-	+	+	-	-	-	-	-	-	-	-	-	-	-
Cluster_4985	hypothetical protein	+	-	-	-	-	-	-	-	-	-	-	-	-	-
Cluster_5004	hypothetical protein	+	-	-	+	+	+	+	-	+	-	-	-	-	-
Cluster_5028	CI	+	-	-	-	-	-	-	-	-	-	-	-	-	-
Cluster_5046	hypothetical protein	+	-	-	-	-	-	-	-	-	-	-	-	-	-
Cluster_5052	hypothetical protein	-	-	-	-	-	-	+	-	-	-	-	-	-	-
Cluster_5067	hypothetical protein	+	-	-	-	-	-	-	-	-	-	-	-	-	-
Cluster_507	2-oxoglutarate oxidoreductase, alpha subunit (EC 1.2.7.3)	-	+	+	-	-	-	-	-	-	-	-	-	-	-
Cluster_5073	hypothetical protein	-	-	-	+	-	-	-	-	-	-	-	-	-	-
Cluster_5078	Acetyltransferase (EC 2.3.1.-)	-	-	-	-	-	-	+	-	-	-	-	-	-	-
Cluster_5079	hypothetical protein	-	+	-	-	-	-	-	-	-	-	-	-	-	-
Cluster_5105	hypothetical protein	-	+	+	-	-	-	-	-	-	-	-	-	-	-
Cluster_5113	hypothetical protein	+	-	-	-	-	-	-	-	-	-	-	-	-	-
Cluster_5117	MutT/nudix family protein	-	-	-	-	-	-	+	-	-	-	-	-	-	-
Cluster_5120	Acetyltransferase, GNAT family	-	-	+	-	-	-	-	-	-	-	-	-	-	-
Cluster_5139	hypothetical protein	-	+	+	-	-	-	-	-	-	-	-	-	-	-
Cluster_5142	hypothetical protein	+	-	-	-	-	-	-	-	-	-	-	-	-	-
Cluster_515	hypothetical protein	+	-	-	-	-	-	-	-	-	-	-	-	-	-
Cluster_5153	Putative translation initiation inhibitor, yjgF family	+	-	-	-	-	-	-	-	-	-	-	-	-	-
Cluster_5159	hypothetical protein	-	-	-	-	-	-	+	-	-	-	-	-	-	-
Cluster_5160	hypothetical protein	+	-	-	-	-	-	-	-	-	-	-	-	-	-
Cluster_5162	FIG01200969: hypothetical protein	-	-	-	+	-	-	-	-	-	-	-	-	-	-
Cluster_5169	FIG078759: hypothetical protein	-	+	+	-	+	-	-	-	-	-	-	-	-	-
Cluster_5176	hypothetical protein	-	-	-	+	-	-	-	-	-	-	-	-	-	-
Cluster_5185	hypothetical protein	-	-	-	-	+	-	-	-	-	-	-	-	-	-
Cluster_5190	hypothetical protein	+	-	-	-	-	-	-	-	-	-	-	-	-	-
Cluster_5207	FIG01202693: hypothetical protein	-	-	-	+	-	-	-	-	-	-	-	-	-	-
Cluster_5209	hypothetical protein	-	-	-	-	+	-	-	-	-	-	-	-	-	-
Cluster_5218	Type IV pilin PilA	-	+	+	-	-	-	-	-	-	-	-	-	-	-
Cluster_5223	hypothetical protein	-	+	+	-	-	-	-	-	-	-	-	-	-	-
Cluster_5228	hypothetical protein	-	+	+	-	-	-	-	-	-	-	-	-	-	-
Cluster_5281	hypothetical protein	-	+	+	-	-	-	-	-	-	-	-	-	-	-
Cluster_5289	hypothetical protein	-	+	+	-	-	-	-	-	-	-	-	-	-	-
Cluster_5290	no significant match	+	-	-	-	-	-	-	-	-	-	-	-	-	-
Cluster_5300	transcriptional regulator, Cro/CI family	-	-	-	-	+	-	-	-	-	-	-	-	-	-
Cluster_5305	hypothetical protein	+	-	-	-	-	-	-	-	-	-	-	-	-	-
Cluster_5317	hypothetical protein	-	+	+	-	-	-	-	-	-	-	-	-	-	-
Cluster_5318	RTX toxin transporter, determinant D	-	+	+	-	-	-	-	-	-	-	-	-	-	-
Cluster_5337	hypothetical protein	+	-	-	-	-	-	-	-	-	-	-	-	-	-
Cluster_5338	hypothetical protein	+	-	-	-	-	-	-	-	-	-	-	-	-	-
Cluster_5350	3-keto-5-aminohexanoate cleavage enzyme	-	+	+	-	-	-	-	-	-	-	-	-	-	-
Cluster_5355	hypothetical protein	+	-	-	-	-	-	-	-	-	-	-	-	-	-
Cluster_5358	hypothetical protein	+	-	-	-	-	-	-	-	-	-	-	-	-	-
Cluster_5361	Phage protein	-	-	-	+	-	-	-	-	-	-	-	-	-	-
Cluster_5407	hypothetical protein	-	-	-	-	-	-	+	-	-	-	-	-	-	-
Cluster_5411	hypothetical protein	-	+	+	-	-	-	-	-	-	-	-	-	-	-
Cluster_5430	Histone acetyltransferase HPA2 and related acetyltransferases	-	-	-	-	-	-	+	-	-	-	-	-	-	-
Cluster_5432	hypothetical protein	+	-	-	-	-	-	-	-	-	-	-	-	-	-
Cluster_5444	hypothetical protein	+	-	-	-	-	-	-	-	-	-	-	-	-	-
Cluster_545	hypothetical protein	-	-	-	+	-	+	-	+	+	-	-	-	-	-
Cluster_5455	hypothetical protein	+	-	-	-	-	-	-	-	-	-	-	-	-	-
Cluster_5480	hypothetical protein	-	+	-	-	-	-	-	-	-	-	-	-	-	-
Cluster_5482	hypothetical protein	-	+	+	-	-	-	-	-	-	-	-	-	-	-
Cluster_5487	hypothetical protein	+	-	-	-	-	-	-	-	-	-	-	-	-	-
Cluster_5497	blasticidin S deaminase	-	+	+	-	-	+	-	-	-	-	-	-	-	-
Cluster_5501	hypothetical protein	-	+	+	-	-	-	-	-	-	-	-	-	-	-
Cluster_5504	hypothetical protein	-	+	+	-	-	-	-	-	-	-	-	-	-	-
Cluster_5505	hypothetical protein	-	+	+	-	-	-	-	-	-	-	-	-	-	-
Cluster_5519	hypothetical protein	-	-	-	+	-	+	-	+	+	-	-	-	-	-
Cluster_5523	hypothetical protein	+	-	-	-	-	-	-	-	-	-	-	-	-	-
Cluster_5524	NTP pyrophosphohydrolase	+	-	-	-	-	-	-	-	-	-	-	-	-	-
Cluster_5525	hypothetical protein	+	-	-	-	-	-	-	-	-	-	-	-	-	-
Cluster_5534	hypothetical protein	+	-	-	-	-	-	-	-	-	-	-	-	-	-
Cluster_5549	hypothetical protein	-	+	+	-	-	-	-	-	-	-	-	-	-	-
Cluster_5552	NinG recombination protein	-	+	+	-	-	-	-	-	-	-	-	-	-	-
Cluster_5553	hypothetical protein	+	-	-	-	-	-	-	-	-	-	-	-	-	-

Cluster_5559	Metallo-beta-lactamase precursor	-	-	-	+	-	-	-	-	-	-	-	-	-	-
Cluster_5562	hypothetical protein	-	-	-	+	-	+	-	+	+	-	-	-	-	-
Cluster_5563	hypothetical protein	-	-	-	+	-	+	-	+	+	-	-	-	-	-
Cluster_5570	hypothetical protein	+	-	-	-	-	-	-	-	-	-	-	-	-	-
Cluster_5572	hypothetical protein	+	-	-	-	-	-	-	-	-	-	-	-	-	-
Cluster_5576	FIG01201305: hypothetical protein	-	-	-	-	-	-	+	-	-	-	-	-	-	-
Cluster_5592	RTX toxin transporter, determinant D	-	+	+	-	-	-	-	-	-	-	-	-	-	-
Cluster_560	Ig-like repeat domain protein 1	-	+	+	-	-	-	-	-	-	-	-	-	-	-
Cluster_5613	hypothetical protein	-	-	-	-	+	-	+	-	-	-	-	-	-	-
Cluster_5629	hypothetical protein	-	-	-	+	+	+	-	+	+	-	-	-	-	-
Cluster_5640	MutT/nudix family protein	-	+	+	-	-	-	-	-	-	-	-	-	-	-
Cluster_5649	transthyretin	-	+	-	-	-	-	-	-	-	-	-	-	-	-
Cluster_565	TniA putative transposase	-	+	+	-	-	-	-	-	-	-	-	-	-	-
Cluster_5651	phage transcriptional regulator, AlpA	-	+	-	-	-	-	-	-	-	-	-	-	-	-
Cluster_5658	hypothetical protein	-	+	+	-	-	+	+	-	-	-	-	-	-	-
Cluster_5663	hypothetical protein	+	-	-	-	-	-	-	-	-	-	-	-	-	-
Cluster_5682	hypothetical protein	-	+	+	-	-	-	-	-	-	-	-	-	-	-
Cluster_5684	hypothetical protein	+	-	-	-	-	-	-	-	-	-	-	-	-	-
Cluster_5700	hypothetical protein	+	-	-	-	-	-	-	-	-	-	-	-	-	-
Cluster_5702	hypothetical protein	+	-	-	-	-	-	-	-	-	-	-	-	-	-
Cluster_5710	MaoC-like dehydratase	+	-	-	-	-	-	-	-	-	-	-	-	-	-
Cluster_5711	no significant match	-	-	-	-	+	-	-	-	-	-	-	-	-	-
Cluster_5716	hypothetical protein	-	-	-	+	-	-	-	-	-	-	-	-	-	-
Cluster_5717	hypothetical protein	-	-	-	+	-	-	-	-	-	-	-	-	-	-
Cluster_5739	Lactoylglutathione lyase	-	+	+	-	-	-	-	-	-	-	-	-	-	-
Cluster_5754	hypothetical protein	-	-	-	+	-	+	-	+	+	-	-	-	-	-
Cluster_5758	hypothetical protein	-	-	-	+	-	-	-	-	-	-	-	-	-	-
Cluster_5769	hypothetical protein	-	+	+	-	-	-	-	-	-	-	-	-	-	-
Cluster_5773	hypothetical protein	-	-	-	-	+	-	-	-	-	-	-	-	-	-
Cluster_5774	hypothetical protein	+	-	-	-	-	-	-	-	-	-	-	-	-	-
Cluster_5780	hypothetical protein	-	+	+	-	-	-	-	-	-	-	-	-	-	-
Cluster_5791	Beta-lactamase (EC 3.5.2.6)	-	-	-	-	-	-	+	-	-	-	-	-	-	-
Cluster_5792	Urease accessory protein UreE	-	+	+	-	+	-	-	-	-	-	-	-	-	-
Cluster_5798	hypothetical protein	-	-	-	-	+	-	+	-	-	-	-	-	-	-
Cluster_5807	hypothetical protein	-	+	+	-	-	-	-	-	-	-	-	-	-	-
Cluster_5821	hypothetical protein	-	+	+	-	-	-	-	-	-	-	-	-	-	-
Cluster_5833	Endoribonuclease L-PSP	+	-	-	-	+	-	+	-	-	-	-	-	-	-
Cluster_5835	COG4925: Uncharacterized conserved protein	-	-	-	-	+	-	+	-	-	-	-	-	-	-
Cluster_5839	hypothetical protein	-	-	-	+	-	-	-	-	-	-	-	-	-	-
Cluster_5840	hypothetical protein	-	-	+	-	-	-	-	-	-	-	-	-	-	-
Cluster_5846	hypothetical protein	+	-	-	-	-	-	-	-	-	-	-	-	-	-
Cluster_5859	DNA sulfur modification protein DndE	-	-	-	-	-	+	-	-	-	-	-	-	-	-
Cluster_5864	Accessory cholera enterotoxin	-	-	-	-	+	-	-	-	-	-	-	-	-	-
Cluster_5889	Protein secretion chaperonin CsaA	-	-	-	+	-	+	-	+	+	-	-	-	-	-
Cluster_5902	FIG01204896: hypothetical protein	+	-	-	-	-	-	-	-	-	-	-	-	-	-
Cluster_5904	phage transcriptional regulator, AlpA	-	+	+	-	-	+	+	-	-	-	-	-	-	-
Cluster_5905	FIG01206989: hypothetical protein	-	-	-	+	-	-	-	-	-	-	-	-	-	-
Cluster_5918	hypothetical protein	-	-	-	+	-	-	-	-	-	-	-	-	-	-
Cluster_5919	hypothetical protein	-	-	-	-	+	-	-	-	-	-	-	-	-	-
Cluster_5931	hypothetical protein	-	-	+	-	-	-	-	-	-	-	-	-	-	-
Cluster_5932	hypothetical protein	-	+	-	-	-	-	-	-	-	-	-	-	-	-
Cluster_5934	hypothetical protein	-	-	-	-	-	-	+	-	-	-	-	-	-	-
Cluster_5936	transcriptional regulator, XRE family	+	-	-	-	-	-	-	-	-	-	-	-	-	-
Cluster_594	putative helicase	-	-	-	+	-	+	-	+	+	-	-	-	-	-
Cluster_5951	hypothetical protein	-	-	-	+	-	-	-	-	-	-	-	-	-	-
Cluster_5953	hypothetical protein	-	+	-	-	-	-	-	-	-	-	-	-	-	-
Cluster_5954	Urease beta subunit (EC 3.5.1.5)	-	+	+	-	+	-	-	-	-	-	-	-	-	-
Cluster_5958	hypothetical protein	+	-	-	-	-	-	-	-	-	-	-	-	-	-
Cluster_5966	hypothetical protein	-	+	+	-	-	-	-	-	-	-	-	-	-	-
Cluster_5969	hypothetical protein	-	+	+	-	-	-	-	-	-	-	-	-	-	-
Cluster_5970	hypothetical protein	+	-	-	-	-	-	-	-	-	-	-	-	-	-
Cluster_5972	no significant match	-	-	-	-	-	-	+	-	-	-	-	-	-	-
Cluster_5985	hypothetical protein	-	+	+	-	+	-	-	-	-	-	-	-	-	-
Cluster_5988	hypothetical protein	+	-	-	-	-	-	-	-	-	-	-	-	-	-
Cluster_5995	Acetyl-CoA acetyltransferase (EC 2.3.1.9) @ Beta-ketoadipyl CoA thiolase (EC 2.3.1.-)	-	+	+	-	-	-	-	-	-	-	-	-	-	-
Cluster_5996	Phage portal protein	-	-	-	+	-	-	-	-	-	-	-	-	-	-
Cluster_60	hypothetical protein	-	-	-	+	-	+	-	+	+	-	-	-	-	-
Cluster_6001	hypothetical protein	-	+	+	-	-	-	-	-	-	-	-	-	-	-
Cluster_6004	conserved hypothetical protein	-	-	-	+	-	+	-	+	+	-	-	-	-	-
Cluster_6037	hypothetical protein	-	-	-	-	+	-	-	-	-	-	-	-	-	-
Cluster_6051	hypothetical protein	-	-	-	-	-	-	+	-	-	-	-	-	-	-
Cluster_6052	hypothetical protein	-	+	+	-	-	-	-	-	-	-	-	-	-	-
Cluster_6055	hypothetical protein	-	-	+	-	-	-	-	-	-	-	-	-	-	-
Cluster_6069	hypothetical protein	-	+	+	-	-	-	-	-	-	-	-	-	-	-
Cluster_6071	Urease gamma subunit (EC 3.5.1.5)	-	+	+	-	+	-	-	-	-	-	-	-	-	-
Cluster_6093	DNA-damage-inducible protein D	-	+	+	-	-	-	-	-	-	-	-	-	-	-
Cluster_6097	hypothetical protein	+	-	-	-	-	-	-	-	-	-	-	-	-	-
Cluster_610	putative HsdS polypeptide, part of CfrA family	-	-	-	+	-	+	-	+	+	-	-	-	-	-
Cluster_6102	hypothetical protein	-	-	-	+	-	-	-	-	-	-	-	-	-	-
Cluster_612	FIG01202366: hypothetical protein	-	-	-	-	-	-	+	-	-	-	-	-	-	-
Cluster_6124	Mobile element protein	-	-	-	-	+	-	-	-	-	-	-	-	-	-
Cluster_6134	hypothetical protein	-	-	-	+	-	-	-	-	-	-	-	-	-	-
Cluster_6137	hypothetical protein	-	-	-	+	-	-	-	-	-	-	-	-	-	-
Cluster_6138	hypothetical protein	-	-	-	+	-	-	-	-	-	-	-	-	-	-
Cluster_6148	bacteriocin immunity protein	-	-	-	+	-	+	-	+	+	-	-	-	-	-
Cluster_6186	Mobile element protein	-	-	-	-	+	-	+	-	-	-	-	-	-	-
Cluster_6188	hypothetical protein	-	-	+	-	-	-	-	-	-	-	-	-	-	-
Cluster_6192	DNA-binding protein, putative	-	-	-	-	+	-	-	-	-	-	-	-	-	-
Cluster_6215	RelE/StbE replicon stabilization toxin	+	-	-	-	-	-	-	-	-	-	-	-	-	-
Cluster_6216	hypothetical protein	-	+	+	-	-	-	-	-	-	-	-	-	-	-
Cluster_6218	hypothetical protein	-	-	-	-	+	-	+	-	-	-	-	-	-	-
Cluster_6229	hypothetical protein	-	+	-	-	-	-	-	-	-	-	-	-	-	-
Cluster_6231	hypothetical protein	-	-	-	-	+	-	-	-	-	-	-	-	-	-

Cluster_6232	FIG01200478: hypothetical protein	-	+	+	-	+	-	-	-	-	-	-	-	-	-
Cluster_6233	hypothetical protein	-	-	-	+	-	-	-	-	-	-	-	-	-	-
Cluster_624	possible DNA helicase	-	+	+	-	-	-	-	-	-	-	-	-	-	-
Cluster_6246	FIG01204969: hypothetical protein	-	-	-	+	-	+	-	+	+	-	-	-	-	-
Cluster_6251	hypothetical protein	-	-	+	-	+	-	+	-	-	-	-	-	-	-
Cluster_6252	hypothetical protein	+	-	-	-	-	-	-	-	-	-	-	-	-	-
Cluster_6254	hypothetical protein	-	-	-	+	-	-	-	-	-	-	-	-	-	-
Cluster_6268	hypothetical protein	-	-	-	+	-	-	-	-	-	-	-	-	-	-
Cluster_6271	hypothetical protein	-	-	-	-	+	-	-	-	-	-	-	-	-	-
Cluster_6290	hypothetical protein	+	-	-	-	-	-	-	-	-	-	-	-	-	-
Cluster_6326	hypothetical protein	-	+	+	-	-	-	-	-	-	-	-	-	-	-
Cluster_6346	hypothetical protein	-	-	-	+	-	-	-	-	-	-	-	-	-	-
Cluster_6350	hypothetical protein	+	-	-	-	-	-	-	-	-	-	-	-	-	-
Cluster_6351	hypothetical protein	+	-	-	-	-	-	-	-	-	-	-	-	-	-
Cluster_6355	hypothetical protein	-	+	+	-	-	-	-	-	-	-	-	-	-	-
Cluster_6356	hypothetical protein	-	-	-	-	+	-	-	-	-	-	-	-	-	-
Cluster_6362	hypothetical protein	+	-	-	-	-	-	-	-	-	-	-	-	-	-
Cluster_6363	hypothetical protein	-	+	+	-	-	-	-	-	-	-	-	-	-	-
Cluster_6393	hypothetical protein	-	+	-	-	-	-	-	-	-	-	-	-	-	-
Cluster_6399	hypothetical protein	-	-	-	-	-	-	+	-	-	-	-	-	-	-
Cluster_6401	hypothetical protein	+	-	-	-	-	-	-	-	-	-	-	-	-	-
Cluster_6412	hypothetical protein	+	-	-	-	-	-	-	-	-	-	-	-	-	-
Cluster_6416	Mobile element protein	-	-	-	-	+	-	-	-	-	-	-	-	-	-
Cluster_6421	hypothetical protein	-	+	+	-	-	-	-	-	-	-	-	-	-	-
Cluster_6427	hypothetical protein	+	-	-	-	-	-	-	-	-	-	-	-	-	-
Cluster_6443	hypothetical protein	-	-	-	-	+	-	-	-	-	-	-	-	-	-
Cluster_6448	hypothetical protein	+	-	-	-	-	-	-	-	-	-	-	-	-	-
Cluster_6477	hypothetical protein	-	-	-	+	-	-	-	-	-	-	-	-	-	-
Cluster_6495	hypothetical protein	-	-	-	+	-	-	-	-	-	-	-	-	-	-
Cluster_6499	conserved domain protein	+	-	-	+	+	+	+	-	+	-	-	-	-	-
Cluster_65	Type I restriction-modification system, restriction subunit R (EC 3.1.21.3)	-	+	+	-	-	-	-	-	-	-	-	-	-	-
Cluster_6500	CopG-like DNA-binding protein	+	-	-	-	-	-	-	-	-	-	-	-	-	-
Cluster_6518	hypothetical protein	-	+	+	-	-	-	-	-	-	-	-	-	-	-
Cluster_6519	putative transcription regulator protein	-	-	-	+	-	+	+	+	+	-	-	-	-	-
Cluster_6521	hypothetical protein	+	-	-	-	-	-	-	-	-	-	-	-	-	-
Cluster_6526	hypothetical protein	-	-	-	-	+	-	-	-	-	-	-	-	-	-
Cluster_6537	hypothetical protein	-	+	+	-	-	-	-	-	-	-	-	-	-	-
Cluster_6538	transcriptional regulator	-	-	-	-	-	-	+	-	-	-	-	-	-	-
Cluster_654	putative site-specific recombinase	-	-	-	+	-	+	-	+	+	-	-	-	-	-
Cluster_6548	FIG01204362: hypothetical protein	-	-	-	-	-	-	+	-	-	-	-	-	-	-
Cluster_6562	hypothetical protein	-	+	+	-	-	-	-	-	-	-	-	-	-	-
Cluster_6565	hypothetical protein	-	-	-	-	-	-	+	-	-	-	-	-	-	-
Cluster_6567	hypothetical protein	-	+	+	-	-	-	-	-	-	-	-	-	-	-
Cluster_6568	hypothetical protein	-	-	-	-	+	-	-	-	-	-	-	-	-	-
Cluster_6570	hypothetical protein	-	-	-	+	-	-	-	-	-	-	-	-	-	-
Cluster_6571	hypothetical protein	-	-	-	+	-	-	-	-	-	-	-	-	-	-
Cluster_6575	hypothetical protein	-	-	-	-	+	-	-	-	-	-	-	-	-	-
Cluster_6590	hypothetical protein	-	+	+	-	+	-	-	-	-	-	-	-	-	-
Cluster_6592	no significant match	-	-	-	-	+	-	-	-	-	-	-	-	-	-
Cluster_6597	hypothetical protein	-	-	-	-	+	-	-	-	-	-	-	-	-	-
Cluster_6614	hypothetical protein	+	-	-	-	-	-	-	-	-	-	-	-	-	-
Cluster_6615	hypothetical protein	-	+	+	-	-	-	-	-	-	-	-	-	-	-
Cluster_6626	hypothetical protein	-	-	-	-	-	-	+	-	-	-	-	-	-	-
Cluster_6650	putative ATP-binding protein	-	-	-	+	-	+	-	+	+	-	-	-	-	-
Cluster_6668	hypothetical protein	-	-	-	+	-	-	-	-	-	-	-	-	-	-
Cluster_6674	hypothetical protein	-	+	+	-	-	-	-	-	-	-	-	-	-	-
Cluster_6675	hypothetical protein	-	-	-	+	-	+	-	+	+	-	-	-	-	-
Cluster_6689	hypothetical protein	-	-	-	+	-	+	-	+	+	-	-	-	-	-
Cluster_6698	hypothetical protein	-	-	-	-	-	-	+	-	-	-	-	-	-	-
Cluster_6700	hypothetical protein	-	-	-	+	-	-	-	-	-	-	-	-	-	-
Cluster_6703	ABC-type maltose transport system, permease component	+	-	-	-	-	-	-	-	-	-	-	-	-	-
Cluster_6704	putative transcription regulator protein	-	-	-	+	-	+	+	+	+	-	-	-	-	-
Cluster_6705	predicted transcriptional regulator	-	+	+	-	-	-	-	-	-	-	-	-	-	-
Cluster_6713	no significant match	-	-	-	-	-	-	+	-	-	-	-	-	-	-
Cluster_6720	hypothetical protein	-	+	+	-	-	+	+	-	-	-	-	-	-	-
Cluster_6724	FIG01204372: hypothetical protein	-	-	+	-	-	-	+	-	-	-	-	-	-	-
Cluster_6725	hypothetical protein	-	+	+	-	-	+	+	-	-	-	-	-	-	-
Cluster_6726	FIG01207287: hypothetical protein	-	-	-	+	-	-	-	-	-	-	-	-	-	-
Cluster_6740	DNA mismatch repair protein MutL	-	-	-	-	-	-	+	-	-	-	-	-	-	-
Cluster_6745	hypothetical protein	-	+	+	-	-	-	-	-	-	-	-	-	-	-
Cluster_6751	hypothetical protein	+	-	-	-	-	-	-	-	-	-	-	-	-	-
Cluster_6770	hypothetical protein	-	+	+	-	-	-	-	-	-	-	-	-	-	-
Cluster_6780	hypothetical protein	-	+	+	-	-	-	-	-	-	-	-	-	-	-
Cluster_6782	hypothetical protein	-	-	-	+	-	-	-	-	-	-	-	-	-	-
Cluster_6786	hypothetical protein	+	-	-	-	-	-	-	-	-	-	-	-	-	-
Cluster_6790	hypothetical protein	+	-	-	-	-	-	-	-	-	-	-	-	-	-
Cluster_6793	hypothetical protein	-	-	-	+	-	+	-	+	+	-	-	-	-	-
Cluster_6799	hypothetical protein	-	-	-	+	-	+	-	+	+	-	-	-	-	-
Cluster_68	hypothetical protein	-	+	-	-	-	-	-	-	-	-	-	-	-	-
Cluster_6800	hypothetical protein	+	-	-	-	-	-	-	-	-	-	-	-	-	-
Cluster_6802	FIG01199671: hypothetical protein	-	-	-	-	+	-	-	-	-	-	-	-	-	-
Cluster_6819	hypothetical protein	-	-	-	-	+	-	-	-	-	-	-	-	-	-
Cluster_6825	Alkyl sulfatase (EC 3.1.6.-)	-	+	+	-	-	-	-	-	-	-	-	-	-	-
Cluster_6839	hypothetical protein	-	-	-	-	-	-	+	-	-	-	-	-	-	-
Cluster_6841	hypothetical protein	+	-	-	-	-	-	-	-	-	-	-	-	-	-
Cluster_6842	ISTde1, transposase	+	-	-	-	-	-	-	-	-	-	-	-	-	-
Cluster_685	Urease alpha subunit (EC 3.5.1.5)	-	+	+	-	+	-	-	-	-	-	-	-	-	-
Cluster_6860	hypothetical protein	-	+	+	-	-	-	-	-	-	-	-	-	-	-
Cluster_6862	hypothetical protein	-	-	+	-	-	-	-	-	-	-	-	-	-	-
Cluster_6871	hypothetical protein	-	-	-	-	+	-	-	-	-	-	-	-	-	-
Cluster_6876	hypothetical protein	+	-	-	-	-	-	-	-	-	-	-	-	-	-
Cluster_6878	hypothetical protein	+	-	-	-	-	-	-	-	-	-	-	-	-	-
Cluster_6879	hypothetical protein	-	-	-	+	-	+	-	+	+	-	-	-	-	-

Cluster_6887	hypothetical protein	-	-	-	-	-	-	+	-	-	-	-	-	-	-
Cluster_6946	hypothetical protein	-	-	-	-	+	-	-	-	-	-	-	-	-	-
Cluster_6985	FIG01201994: hypothetical protein	-	-	-	-	+	-	-	-	-	-	-	-	-	-
Cluster_6990	hypothetical protein	-	+	+	-	-	-	-	-	-	-	-	-	-	-
Cluster_6991	hypothetical protein	-	+	+	-	-	+	+	-	-	-	-	-	-	-
Cluster_7000	hypothetical protein	-	-	-	-	+	-	-	-	-	-	-	-	-	-
Cluster_7033	hypothetical protein	-	-	-	-	-	-	+	-	-	-	-	-	-	-
Cluster_7037	hypothetical protein	+	-	-	-	-	-	-	-	-	-	-	-	-	-
Cluster_7047	hypothetical protein	-	-	-	+	-	+	-	+	+	-	-	-	-	-
Cluster_7050	hypothetical protein	-	+	+	-	-	-	-	-	-	-	-	-	-	-
Cluster_7053	hypothetical protein	-	-	-	+	-	+	-	+	+	-	-	-	-	-
Cluster_7084	Mobile element protein	-	-	-	-	+	-	+	-	-	-	-	-	-	-
Cluster_7089	no significant match	-	-	-	-	+	-	-	-	-	-	-	-	-	-
Cluster_7093	hypothetical protein	-	-	-	-	+	-	-	-	-	-	-	-	-	-
Cluster_7098	hypothetical protein	-	+	+	-	-	-	-	-	-	-	-	-	-	-
Cluster_7104	hypothetical protein	-	-	-	-	-	-	+	-	-	-	-	-	-	-
Cluster_7140	Phage terminase large subunit	-	-	-	+	-	-	-	-	-	-	-	-	-	-
Cluster_7141	hypothetical protein	-	-	-	-	+	-	-	-	-	-	-	-	-	-
Cluster_7143	no significant match	-	-	-	-	-	-	+	-	-	-	-	-	-	-
Cluster_7158	hypothetical protein	-	-	-	+	-	+	-	+	+	-	-	-	-	-
Cluster_7159	hypothetical protein	-	-	-	-	+	-	+	-	-	-	-	-	-	-
Cluster_716	superfamily II helicase and inactivated derivatives-like protein	-	+	+	-	-	-	+	-	-	-	-	-	-	-
Cluster_7161	conserved hypothetical protein	-	-	-	+	-	+	-	+	+	-	-	-	-	-
Cluster_7166	hypothetical protein	-	-	-	-	-	-	+	-	-	-	-	-	-	-
Cluster_7171	hypothetical protein	+	-	-	-	-	-	-	-	-	-	-	-	-	-
Cluster_7173	no significant match	-	-	-	-	-	+	-	-	-	-	-	-	-	-
Cluster_7203	hypothetical protein	-	+	+	-	-	-	-	-	-	-	-	-	-	-
Cluster_7207	hypothetical protein	-	-	-	-	+	-	+	-	-	-	-	-	-	-
Cluster_7217	hypothetical protein	-	+	+	-	-	-	-	-	-	-	-	-	-	-
Cluster_7218	FIG01073254: hypothetical protein	-	+	+	-	-	-	-	-	-	-	-	-	-	-
Cluster_7222	hypothetical protein	+	-	-	-	-	-	-	-	-	-	-	-	-	-
Cluster_7223	hypothetical protein	+	-	-	-	-	-	-	-	-	-	-	-	-	-
Cluster_7244	hypothetical protein	-	-	-	-	-	-	+	-	-	-	-	-	-	-
Cluster_725	hypothetical protein	+	-	-	-	-	-	-	-	-	-	-	-	-	-
Cluster_7275	SAM-dependent methyltransferase (EC 2.1.1.-)	-	-	-	-	-	-	+	-	-	-	-	-	-	-
Cluster_7278	no significant match	-	-	-	-	-	-	+	-	-	-	-	-	-	-
Cluster_7282	hypothetical protein	-	+	+	-	+	-	-	-	-	-	-	-	-	-
Cluster_7298	hypothetical protein	-	+	+	-	+	-	-	-	-	-	-	-	-	-
Cluster_7330	hypothetical protein	-	+	+	-	+	-	-	-	-	-	-	-	-	-
Cluster_7355	hypothetical protein	-	-	-	+	-	-	-	-	-	-	-	-	-	-
Cluster_736	superfamily II helicase and inactivated derivatives-like protein	-	-	-	-	-	+	-	-	-	-	-	-	-	-
Cluster_7365	no significant match	-	+	-	-	-	-	-	-	-	-	-	-	-	-
Cluster_7395	Outer membrane protein assembly factor YaeT precursor	-	-	-	-	-	-	+	-	-	-	-	-	-	-
Cluster_7397	Mobile element protein	-	+	+	-	+	-	-	-	-	-	-	-	-	-
Cluster_7417	TldE protein, part of TldE/TldD proteolytic complex	-	-	-	-	-	-	+	-	-	-	-	-	-	-
Cluster_7432	hypothetical protein	-	-	-	-	+	-	-	-	-	-	-	-	-	-
Cluster_7440	hypothetical protein	-	+	+	-	-	-	-	-	-	-	-	-	-	-
Cluster_7448	hypothetical protein	-	-	-	+	-	+	-	+	+	-	-	-	-	-
Cluster_7491	no significant match	-	-	-	-	-	-	+	-	-	-	-	-	-	-
Cluster_7507	hypothetical protein	-	+	+	-	-	-	-	-	-	-	-	-	-	-
Cluster_7514	Beta-lactamase (EC 3.5.2.6)	-	-	-	-	-	-	+	-	-	-	-	-	-	-
Cluster_7533	hypothetical protein	-	-	-	+	-	-	-	-	-	-	-	-	-	-
Cluster_7536	hypothetical protein	-	-	-	+	-	-	-	-	-	-	-	-	-	-
Cluster_7556	hypothetical protein	-	-	-	-	-	-	-	-	+	-	-	-	-	-
Cluster_7559	hypothetical protein	-	+	+	-	-	-	-	-	-	-	-	-	-	-
Cluster_7562	hypothetical protein	-	+	+	-	+	-	-	-	-	-	-	-	-	-
Cluster_7580	hypothetical protein	-	+	+	-	+	-	+	-	-	-	-	-	-	-
Cluster_7584	no significant match	-	-	-	-	-	-	+	-	-	-	-	-	-	-
Cluster_7596	hypothetical protein	+	-	-	-	-	-	-	-	-	-	-	-	-	-
Cluster_7597	hypothetical protein	+	-	-	-	-	-	-	-	-	-	-	-	-	-
Cluster_7603	hypothetical protein	-	+	+	-	+	-	-	-	-	-	-	-	-	-
Cluster_7605	hypothetical protein	-	-	-	-	+	-	-	-	-	-	-	-	-	-
Cluster_7636	hypothetical protein	-	+	+	-	+	-	-	-	-	-	-	-	-	-
Cluster_7639	hypothetical protein	-	+	+	-	+	-	-	-	-	-	-	-	-	-
Cluster_7640	hypothetical protein	-	+	+	-	-	-	-	-	-	-	-	-	-	-
Cluster_7643	hypothetical protein	-	-	-	-	+	-	-	-	-	-	-	-	-	-
Cluster_7644	Mobile element protein	-	+	+	-	+	-	-	-	-	-	-	-	-	-
Cluster_7656	hypothetical protein	+	-	-	-	-	-	-	-	-	-	-	-	-	-
Cluster_7660	hypothetical protein	-	+	+	-	-	-	-	-	-	-	-	-	-	-
Cluster_7661	Ribonuclease HI (EC 3.1.26.4)	-	-	+	-	-	-	+	-	-	-	-	-	-	-
Cluster_7668	no significant match	-	+	+	-	-	-	-	-	-	-	-	-	-	-
Cluster_7676	hypothetical protein	-	-	-	-	-	-	+	-	-	-	-	-	-	-
Cluster_7679	hypothetical protein	-	-	-	-	-	-	+	-	-	-	-	-	-	-
Cluster_7680	hypothetical protein	-	-	-	+	-	-	-	-	-	-	-	-	-	-
Cluster_769	3'-phosphoadenosine 5'-phosphosulfate sulfurtransferase DndC	-	-	-	-	-	+	-	-	-	-	-	-	-	-
Cluster_773	Lipoprotein VsaC	-	-	+	-	-	-	-	-	-	-	-	-	-	-
Cluster_7752	hypothetical protein	-	-	-	-	+	-	-	-	-	-	-	-	-	-
Cluster_7757	Thiol:disulfide interchange protein DsbC	-	-	-	-	-	-	+	-	-	-	-	-	-	-
Cluster_7770	no significant match	+	-	-	-	-	-	-	-	-	-	-	-	-	-
Cluster_78	hypothetical protein	+	-	-	-	-	-	-	-	-	-	-	-	-	-
Cluster_783	hypothetical protein	-	+	+	-	-	-	-	-	-	-	-	-	-	-
Cluster_788	ABC-type transporter ATP-binding protein	+	-	-	-	-	-	-	-	-	-	-	-	-	-
Cluster_798	hypothetical protein	-	-	-	+	-	+	-	+	+	-	-	-	-	-
Cluster_799	hypothetical protein	-	-	-	+	-	+	-	+	+	-	-	-	-	-
Cluster_803	COG1132	-	-	-	-	-	+	-	-	-	-	-	-	-	-
Cluster_807	FIG01199758: hypothetical protein	-	-	-	-	+	-	-	-	-	-	-	-	-	-
Cluster_815	hypothetical protein	-	-	-	+	-	+	-	+	+	-	-	-	-	-
Cluster_817	capsular polysaccharide biosynthesis protein	-	+	+	-	-	-	-	-	-	-	-	-	-	-
Cluster_818	L-lactate permease	+	-	-	-	-	-	-	-	-	-	-	-	-	-
Cluster_824	capsular polysaccharide biosynthesis protein	-	-	-	+	-	-	-	-	-	-	-	-	-	-
Cluster_829	capsular polysaccharide biosynthesis protein	+	-	-	-	-	-	-	-	-	-	-	-	-	-
Cluster_846	hypothetical protein	-	-	-	-	+	-	-	-	-	-	-	-	-	-
Cluster_861	FIG01203377: hypothetical protein	-	-	-	-	+	-	-	-	-	-	-	-	-	-

Cluster_864	FIG01205630: hypothetical protein	-	+	+	-	-	-	-	-	-	-	-	-	-	-
Cluster_870	hypothetical protein	-	-	-	-	+	-	+	-	-	-	-	-	-	-
Cluster_890	Nickel ABC transporter, periplasmic nickel-binding protein nikA2 (TC 3.A.1.5.3)	-	+	+	-	+	-	-	-	-	-	-	-	-	-
Cluster_897	hypothetical protein	+	-	-	-	-	-	-	-	-	-	-	-	-	-
Cluster_9	Lipoprotein VsaC	-	-	-	-	+	-	-	-	-	-	-	-	-	-
Cluster_901	hypothetical protein	-	+	+	-	-	-	-	-	-	-	-	-	-	-
Cluster_921	hypothetical protein	-	+	+	-	-	-	-	-	-	-	-	-	-	-
Cluster_932	hypothetical protein	-	-	-	+	-	+	-	+	+	-	-	-	-	-
Cluster_938	hypothetical protein	+	-	-	-	-	-	-	-	-	-	-	-	-	-
Cluster_946	Putative phage protein	-	-	-	-	-	-	+	-	-	-	-	-	-	-
Cluster_957	hypothetical protein	-	-	-	+	-	+	-	+	+	-	-	-	-	-
Cluster_960	type I restriction enzyme M protein	-	-	-	+	-	+	-	+	+	-	-	-	-	-
Cluster_968	Type I restriction-modification system, DNA-methyltransferase subunit M (EC 2.1.1.72)	-	-	-	-	-	-	+	-	-	-	-	-	-	-
Cluster_976	bacteriophage f237 ORF8	-	-	-	+	-	+	-	+	+	-	-	-	-	-
Cluster_978	Protein containing cell adhesion domain	-	+	+	-	-	-	-	-	-	-	-	-	-	-
Cluster_992	hypothetical protein	+	-	-	-	-	-	-	-	-	-	-	-	-	-

**Gene Clusters Exclusive to Environmental Isolate Genomes**		**K1275 (O3:K54)**	**K1461 (O4:K12)**	**10329 (O4:K12)**	**AN5034 (O4:K68)**	**AQ4037 (O3:K6)**	**K5030 (O3:K6)**	**AQ3810 (O3:K6)**	**RIMD2210633 (O3:K6)**	**Peru-466 (O3:K6)**	**SG176 (O5:Kuk)**	**AF91 (O3:K6)**	**22702 (O5:Kuk)**	**J-C2-34 (O5:K19)**	**BB22OP (O4:K8)**
Cluster_1016	hypothetical protein	-	-	-	-	-	-	-	-	-	-	-	-	+	-
Cluster_1032	Cardiolipin synthetase (EC 2.7.8.-)	-	-	-	-	-	-	-	-	-	-	-	+	+	-
Cluster_1034	hypothetical protein	-	-	-	-	-	-	-	-	-	+	-	-	-	-
Cluster_1035	Membrane protein involved in the export of O-antigen, teichoic acid lipoteichoic acids	-	-	-	-	-	-	-	-	-	-	-	-	-	+
Cluster_106	Putative insecticidal toxin complex	-	-	-	-	-	-	-	-	-	-	+	-	-	-
Cluster_109	Alpha-1,2-mannosidase	-	-	-	-	-	-	-	-	-	-	-	+	+	-
Cluster_1093	hypothetical protein	-	-	-	-	-	-	-	-	-	-	+	-	-	-
Cluster_1100	Putative membrane protein	-	-	-	-	-	-	-	-	-	+	-	-	-	-
Cluster_1106	hypothetical protein	-	-	-	-	-	-	-	-	-	-	-	+	-	-
Cluster_1108	Modification methylase Sau96I (EC 2.1.1.37)	-	-	-	-	-	-	-	-	-	-	+	-	-	-
Cluster_1119	hypothetical protein	-	-	-	-	-	-	-	-	-	-	-	-	+	-
Cluster_1127	hypothetical protein	-	-	-	-	-	-	-	-	-	+	-	-	-	-
Cluster_1130	hypothetical protein	-	-	-	-	-	-	-	-	-	-	-	-	+	-
Cluster_1144	hypothetical protein	-	-	-	-	-	-	-	-	-	-	+	-	-	-
Cluster_1168	coenzyme F420-reducing hydrogenase, beta subunit homolog	-	-	-	-	-	-	-	-	-	-	-	-	+	-
Cluster_1169	hypothetical protein	-	-	-	-	-	-	-	-	-	-	-	+	+	-
Cluster_1179	FIG116849: hypothetical protein	-	-	-	-	-	-	-	-	-	-	-	+	+	-
Cluster_118	hypothetical protein	-	-	-	-	-	-	-	-	-	-	-	-	-	+
Cluster_120	Probable tail fiber protein	-	-	-	-	-	-	-	-	-	-	-	-	-	+
Cluster_122	Endonuclease	-	-	-	-	-	-	-	-	-	-	-	+	-	-
Cluster_1231	hypothetical protein	-	-	-	-	-	-	-	-	-	-	-	-	+	-
Cluster_1233	Putative phage protein	-	-	-	-	-	-	-	-	-	-	-	-	+	-
Cluster_1234	54K polar flagellar sheath protein A	-	-	-	-	-	-	-	-	-	-	-	+	+	-
Cluster_1235	FIG01200656: hypothetical protein	-	-	-	-	-	-	-	-	-	+	-	-	-	-
Cluster_1240	2-C-methyl-D-erythritol 4-phosphate cytidylyltransferase (EC 2.7.7.60)	-	-	-	-	-	-	-	-	-	+	-	-	-	-
Cluster_1257	FIG00642059: hypothetical protein	-	-	-	-	-	-	-	-	-	+	-	-	-	-
Cluster_1258	hypothetical protein	-	-	-	-	-	-	-	-	-	+	-	-	-	-
Cluster_128	Aconitate hydratase (EC 4.2.1.3)	-	-	-	-	-	-	-	-	-	-	-	+	+	-
Cluster_1320	hypothetical protein	-	-	-	-	-	-	-	-	-	-	+	-	-	-
Cluster_1327	Conserved domain protein	-	-	-	-	-	-	-	-	-	-	-	+	+	-
Cluster_1328	hypothetical protein	-	-	-	-	-	-	-	-	-	-	-	-	+	-
Cluster_1336	HipA protein	-	-	-	-	-	-	-	-	-	-	+	-	-	-
Cluster_136	Alpha-1,2-mannosidase	-	-	-	-	-	-	-	-	-	-	-	+	+	-
Cluster_1362	hypothetical protein	-	-	-	-	-	-	-	-	-	-	-	+	-	-
Cluster_1377	hypothetical protein	-	-	-	-	-	-	-	-	-	-	-	+	-	-
Cluster_1384	hypothetical protein	-	-	-	-	-	-	-	-	-	-	+	-	-	-
Cluster_1389	hypothetical protein	-	-	-	-	-	-	-	-	-	-	-	-	+	-
Cluster_1396	FIG00919628: hypothetical protein	-	-	-	-	-	-	-	-	-	-	-	+	+	-
Cluster_1439	hypothetical protein	-	-	-	-	-	-	-	-	-	+	-	-	-	-
Cluster_1463	hypothetical protein	-	-	-	-	-	-	-	-	-	-	+	-	-	-
Cluster_1465	Type I restriction-modification system, specificity subunit S (EC 3.1.21.3)	-	-	-	-	-	-	-	-	-	-	-	+	-	-
Cluster_1473	UDP-N-acetyl-D-mannosaminuronate dehydrogenase	-	-	-	-	-	-	-	-	-	-	-	-	-	+
Cluster_148	ATPase involved in DNA repair	-	-	-	-	-	-	-	-	-	-	+	-	-	-
Cluster_1498	hypothetical protein	-	-	-	-	-	-	-	-	-	-	-	+	-	-
Cluster_1500	Error-prone, lesion bypass DNA polymerase V (UmuC)	-	-	-	-	-	-	-	-	-	-	-	+	-	-
Cluster_1512	virulence-associated E	-	-	-	-	-	-	-	-	-	-	-	+	+	-
Cluster_1518	virulence-associated E	-	-	-	-	-	-	-	-	-	-	-	-	+	-
Cluster_1522	Membrane protein involved in the export of O-antigen, teichoic acid lipoteichoic acids	-	-	-	-	-	-	-	-	-	-	-	-	+	-
Cluster_1559	Type I restriction-modification system, specificity subunit S (EC 3.1.21.3)	-	-	-	-	-	-	-	-	-	-	-	-	-	+
Cluster_1580	Type II restriction endonuclease	-	-	-	-	-	-	-	-	-	-	-	+	-	-
Cluster_1581	hypothetical protein	-	-	-	-	-	-	-	-	-	-	-	+	-	-
Cluster_1591	polysaccharide biosynthesis protein	-	-	-	-	-	-	-	-	-	+	-	-	-	-
Cluster_16	FIG01203472: hypothetical protein	-	-	-	-	-	-	-	-	-	-	-	-	-	+
Cluster_1601	hypothetical protein	-	-	-	-	-	-	-	-	-	-	-	-	-	+
Cluster_1609	hypothetical protein	-	-	-	-	-	-	-	-	-	-	+	-	-	-
Cluster_1623	Phage integrase	-	-	-	-	-	-	-	-	-	-	-	-	+	-
Cluster_163	hypothetical protein	-	-	-	-	-	-	-	-	-	-	-	-	+	-
Cluster_1648	hypothetical protein	-	-	-	-	-	-	-	-	-	-	+	-	-	-
Cluster_1656	FIG01200031: hypothetical protein	-	-	-	-	-	-	-	-	-	-	-	+	+	-
Cluster_1661	Probable phiRv1 integrase	-	-	-	-	-	-	-	-	-	-	-	-	+	-
Cluster_1666	hypothetical protein	-	-	-	-	-	-	-	-	-	-	+	-	-	-
Cluster_1671	Chromosome (plasmid) partitioning protein ParA	-	-	-	-	-	-	-	-	-	-	-	+	-	-
Cluster_169	TonB-dependent receptor	-	-	-	-	-	-	-	-	-	-	-	+	+	-
Cluster_1691	Mobile element protein	-	-	-	-	-	-	-	-	-	-	-	+	-	-
Cluster_1704	hypothetical protein	-	-	-	-	-	-	-	-	-	-	-	-	-	+
Cluster_1714	Integrase	-	-	-	-	-	-	-	-	-	+	-	-	-	-
Cluster_1715	Glycosyl transferase, group 1	-	-	-	-	-	-	-	-	-	-	-	-	-	+
Cluster_1722	Integrase	-	-	-	-	-	-	-	-	-	-	-	-	+	-
Cluster_1731	hypothetical protein	-	-	-	-	-	-	-	-	-	-	-	+	+	-
Cluster_1757	Integrase	-	-	-	-	-	-	-	-	-	-	-	+	+	-
Cluster_1761	O-antigen flippase Wzx	-	-	-	-	-	-	-	-	-	-	-	+	-	-
Cluster_1762	FIG01201377: hypothetical protein	-	-	-	-	-	-	-	-	-	-	-	+	+	-
Cluster_1766	hypothetical protein	-	-	-	-	-	-	-	-	-	-	-	-	+	-
Cluster_1769	putative sugar transport protein	-	-	-	-	-	-	-	-	-	-	+	-	-	-

Cluster_1777	Outer membrane protein/protective antigen OMA87	-	-	-	-	-	-	-	-	-	-	-	+	+	-
Cluster_1787	hypothetical protein	-	-	-	-	-	-	-	-	-	-	+	-	-	-
Cluster_18	MSHA biogenesis protein MshQ	-	-	-	-	-	-	-	-	-	-	-	-	-	+
Cluster_1814	Lipid carrier : UDP-N-acetylgalactosaminyltransferase (EC 2.4.1.-)	-	-	-	-	-	-	-	-	-	-	-	+	-	-
Cluster_1817	hypothetical protein	-	-	-	-	-	-	-	-	-	-	-	-	-	+
Cluster_1820	METAL-ACTIVATED PYRIDOXAL ENZYME	-	-	-	-	-	-	-	-	-	-	-	+	+	-
Cluster_1843	conserved hypothetical protein	-	-	-	-	-	-	-	-	-	-	-	+	-	-
Cluster_1845	4-keto-6-deoxy-N-Acetyl-D-hexosaminyl-(Lipid carrier) aminotransferase	-	-	-	-	-	-	-	-	-	-	-	+	+	-
Cluster_1853	hypothetical protein	-	-	-	-	-	-	-	-	-	+	-	-	-	-
Cluster_1882	UDP-2,3-diacetamido-2,3-dideoxy-D-mannuronic acid transferase	-	-	-	-	-	-	-	-	-	-	-	-	+	-
Cluster_1892	Alpha-1,4-N-acetylgalactosamine transferase PglJ (EC 2.4.1.-)	-	-	-	-	-	-	-	-	-	-	-	+	-	-
Cluster_1897	hypothetical protein	-	-	-	-	-	-	-	-	-	+	-	-	-	-
Cluster_1898	hypothetical protein	-	-	-	-	-	-	-	-	-	-	-	+	+	-
Cluster_1906	UDP-N-acetylglucosamine 2-epimerase (EC 5.1.3.14)	-	-	-	-	-	-	-	-	-	-	-	-	-	+
Cluster_1909	hypothetical protein	-	-	-	-	-	-	-	-	-	-	-	+	-	-
Cluster_1916	Outer membrane protein/protective antigen OMA87	-	-	-	-	-	-	-	-	-	-	-	+	+	-
Cluster_1931	Phage protein	-	-	-	-	-	-	-	-	-	-	-	-	+	-
Cluster_1936	FIG01205996: hypothetical protein	-	-	-	-	-	-	-	-	-	-	-	+	+	-
Cluster_1937	Alanine dehydrogenase (EC 1.4.1.1)	-	-	-	-	-	-	-	-	-	+	-	-	-	-
Cluster_1947	putative cytoplasmic protein	-	-	-	-	-	-	-	-	-	+	-	-	-	-
Cluster_1954	hypothetical protein	-	-	-	-	-	-	-	-	-	+	-	-	-	-
Cluster_1955	hypothetical protein	-	-	-	-	-	-	-	-	-	-	-	-	-	+
Cluster_1960	putative DNA helicase	-	-	-	-	-	-	-	-	-	-	+	-	-	-
Cluster_1963	Endoglucanase precursor (EC 3.2.1.4)	-	-	-	-	-	-	-	-	-	-	-	+	+	-
Cluster_1972	Membrane fusion component of tripartite multidrug resistance system	-	-	-	-	-	-	-	-	-	+	-	-	-	-
Cluster_1978	putative glycosyltransferase	-	-	-	-	-	-	-	-	-	-	-	-	-	+
Cluster_1988	hypothetical protein	-	-	-	-	-	-	-	-	-	-	-	-	+	-
Cluster_1989	Glycosyl transferase, group 1	-	-	-	-	-	-	-	-	-	+	-	-	-	-
Cluster_199	hypothetical protein	-	-	-	-	-	-	-	-	-	-	-	-	-	+
Cluster_2007	hypothetical protein	-	-	-	-	-	-	-	-	-	-	-	-	-	+
Cluster_2008	Probable Co/Zn/Cd efflux system membrane fusion protein	-	-	-	-	-	-	-	-	-	-	-	+	+	-
Cluster_2021	glycosyl transferase group 1	-	-	-	-	-	-	-	-	-	-	-	-	-	+
Cluster_2022	capsule biosynthesis protein, putative	-	-	-	-	-	-	-	-	-	+	-	-	-	-
Cluster_2023	capsular polysaccharide biosynthesis protein	-	-	-	-	-	-	-	-	-	-	-	-	+	-
Cluster_203	Alpha-1,2-mannosidase	-	-	-	-	-	-	-	-	-	-	-	+	+	-
Cluster_2034	Tricorn protease N-terminal domain-containing protein	-	-	-	-	-	-	-	-	-	-	-	-	-	+
Cluster_2035	Undecaprenyl-phosphate N-acetylglucosaminyl 1-phosphate transferase (EC 2.7.8.-)	-	-	-	-	-	-	-	-	-	+	-	+	+	-
Cluster_2036	FIG01201329: hypothetical protein	-	-	-	-	-	-	-	-	-	-	-	-	-	+
Cluster_2053	FIG01206446: hypothetical protein	-	-	-	-	-	-	-	-	-	-	-	+	+	-
Cluster_2054	FIG01201088: hypothetical protein	-	-	-	-	-	-	-	-	-	+	-	-	-	-
Cluster_2055	Site-specific recombinase, phage integrase family	-	-	-	-	-	-	-	-	-	-	-	-	-	+
Cluster_2057	hypothetical protein	-	-	-	-	-	-	-	-	-	-	+	-	-	-
Cluster_2058	AfaG	-	-	-	-	-	-	-	-	-	-	+	-	-	-
Cluster_2070	Chromosome (plasmid) partitioning protein ParB	-	-	-	-	-	-	-	-	-	-	-	+	-	-
Cluster_2081	Phage major capsid protein	-	-	-	-	-	-	-	-	-	-	-	+	-	-
Cluster_2087	corresponds to STY3950 from Accession AL513382: Salmonella typhi CT18	-	-	-	-	-	-	-	-	-	-	-	+	+	-
Cluster_209	Alpha-1,2-mannosidase	-	-	-	-	-	-	-	-	-	-	-	+	+	-
Cluster_2091	hypothetical protein	-	-	-	-	-	-	-	-	-	-	-	-	-	+
Cluster_2107	General secretion pathway protein D	-	-	-	-	-	-	-	-	-	-	-	+	+	-
Cluster_2108	hypothetical protein	-	-	-	-	-	-	-	-	-	-	-	-	+	-
Cluster_2125	Glycosyl transferase, group 1	-	-	-	-	-	-	-	-	-	+	-	-	-	-
Cluster_2133	transposase	-	-	-	-	-	-	-	-	-	-	+	-	-	-
Cluster_2148	Asp-tRNAAsn/Glu-tRNAGln amidotransferase A subunit and related amidases	-	-	-	-	-	-	-	-	-	-	-	-	-	+
Cluster_2165	Mobile element protein	-	-	-	-	-	-	-	-	-	-	-	+	+	-
Cluster_2169	hypothetical protein	-	-	-	-	-	-	-	-	-	-	-	-	+	-
Cluster_2170	Glycosyltransferase (EC 2.4.1.-)	-	-	-	-	-	-	-	-	-	+	-	-	-	-
Cluster_2174	Conjugative transfer protein TrbL	-	-	-	-	-	-	-	-	-	+	-	-	-	-
Cluster_2182	Na+/H+ antiporter NhaA type	-	-	-	-	-	-	-	-	-	-	-	+	+	-
Cluster_2187	Glycosyltransferase (EC 2.4.1.-)	-	-	-	-	-	-	-	-	-	-	-	-	+	-
Cluster_220	hypothetical protein	-	-	-	-	-	-	-	-	-	-	+	-	-	-
Cluster_2211	hypothetical protein	-	-	-	-	-	-	-	-	-	-	-	-	-	+
Cluster_2221	putative restriction endonuclease	-	-	-	-	-	-	-	-	-	+	-	-	-	-
Cluster_223	Alpha-1,2-mannosidase	-	-	-	-	-	-	-	-	-	-	-	+	+	-
Cluster_2231	regulatory protein CII	-	-	-	-	-	-	-	-	-	-	-	+	-	-
Cluster_2241	Mobile element protein	-	-	-	-	-	-	-	-	-	-	+	-	-	-
Cluster_2261	metallo-beta-lactamase family protein	-	-	-	-	-	-	-	-	-	-	-	-	-	+
Cluster_2284	plasmid encoded RepA protein	-	-	-	-	-	-	-	-	-	+	-	-	-	-
Cluster_232	hypothetical protein	-	-	-	-	-	-	-	-	-	+	-	-	-	-
Cluster_2334	Phage protein D	-	-	-	-	-	-	-	-	-	-	-	-	+	-
Cluster_2354	rolling circle replication protein, Rep63 protein	-	-	-	-	-	-	-	-	-	-	+	-	-	-
Cluster_2356	hypothetical protein	-	-	-	-	-	-	-	-	-	-	-	-	+	-
Cluster_2367	hypothetical protein	-	-	-	-	-	-	-	-	-	-	-	+	-	-
Cluster_2380	hypothetical protein	-	-	-	-	-	-	-	-	-	-	-	-	+	-
Cluster_2381	Permease of the drug/metabolite transporter (DMT) superfamily	-	-	-	-	-	-	-	-	-	+	-	-	-	-
Cluster_2392	hypothetical protein	-	-	-	-	-	-	-	-	-	+	-	-	-	-
Cluster_2403	hypothetical protein	-	-	-	-	-	-	-	-	-	-	-	+	-	-
Cluster_2404	Phage minor capsid protein - DNA pilot protein	-	-	-	-	-	-	-	-	-	-	-	+	-	-
Cluster_242	Probable tail fiber protein	-	-	-	-	-	-	-	-	-	-	-	-	+	-
Cluster_2433	hypothetical protein	-	-	-	-	-	-	-	-	-	-	-	+	-	-
Cluster_2442	hypothetical protein	-	-	-	-	-	-	-	-	-	-	-	-	-	+
Cluster_2444	Type II restriction enzyme BsuBI (EC 3.1.21.4)	-	-	-	-	-	-	-	-	-	-	-	-	+	-
Cluster_2446	hypothetical protein	-	-	-	-	-	-	-	-	-	-	+	-	-	-
Cluster_2460	Glycosyl transferase, group 2 family protein	-	-	-	-	-	-	-	-	-	-	-	-	+	-
Cluster_2477	hypothetical protein	-	-	-	-	-	-	-	-	-	-	+	-	-	-
Cluster_2492	hypothetical protein	-	-	-	-	-	-	-	-	-	+	-	-	-	-
Cluster_2493	hypothetical protein	-	-	-	-	-	-	-	-	-	-	-	-	+	-
Cluster_2494	hypothetical protein	-	-	-	-	-	-	-	-	-	-	-	+	-	-
Cluster_2496	hypothetical protein	-	-	-	-	-	-	-	-	-	-	-	+	-	-
Cluster_2497	Phenazine biosynthesis protein PhzF like	-	-	-	-	-	-	-	-	-	-	+	-	-	-
Cluster_2498	hypothetical protein	-	-	-	-	-	-	-	-	-	-	-	+	-	-
Cluster_2522	OmpA/MotB domain protein	-	-	-	-	-	-	-	-	-	-	+	-	-	-
Cluster_2529	Mobile element protein	-	-	-	-	-	-	-	-	-	-	+	-	-	-
Cluster_2530	hypothetical protein	-	-	-	-	-	-	-	-	-	+	-	-	-	-

Cluster_2535	Rep protein	-	-	-	-	-	-	-	-	-	-	+	-	-	-
Cluster_2536	hypothetical protein	-	-	-	-	-	-	-	-	-	-	-	-	-	+
Cluster_2551	FIG01202029: hypothetical protein	-	-	-	-	-	-	-	-	-	+	-	+	-	-
Cluster_2554	mobilization protein MobA	-	-	-	-	-	-	-	-	-	-	+	-	-	-
Cluster_2567	Glycosyltransferase	-	-	-	-	-	-	-	-	-	+	-	-	-	-
Cluster_2570	Predicted transcriptional regulator	-	-	-	-	-	-	-	-	-	+	-	-	-	-
Cluster_2571	hypothetical protein	-	-	-	-	-	-	-	-	-	-	-	+	-	-
Cluster_258	Putative insecticidal toxin complex	-	-	-	-	-	-	-	-	-	-	+	-	-	-
Cluster_2584	FIG01202029: hypothetical protein	-	-	-	-	-	-	-	-	-	-	-	-	+	-
Cluster_2597	UDP-glucose 4-epimerase (EC 5.1.3.2)	-	-	-	-	-	-	-	-	-	-	-	-	-	+
Cluster_2598	hypothetical protein	-	-	-	-	-	-	-	-	-	+	-	-	-	-
Cluster_2600	D-3-phosphoglycerate dehydrogenase (EC 1.1.1.95)	-	-	-	-	-	-	-	-	-	+	-	-	-	+
Cluster_2605	hypothetical protein	-	-	-	-	-	-	-	-	-	-	-	+	-	-
Cluster_2619	5'-nucleotidase (EC 3.1.3.5)	-	-	-	-	-	-	-	-	-	-	+	-	-	-
Cluster_262	Outer membrane usher protein faeD precursor	-	-	-	-	-	-	-	-	-	-	+	-	-	-
Cluster_2620	hypothetical protein	-	-	-	-	-	-	-	-	-	-	-	-	+	-
Cluster_2622	hypothetical protein	-	-	-	-	-	-	-	-	-	-	+	-	-	-
Cluster_2643	mobilization protein	-	-	-	-	-	-	-	-	-	-	-	+	-	-
Cluster_2644	Possible neuromedin U precursor	-	-	-	-	-	-	-	-	-	-	-	+	+	-
Cluster_2645	FIG01205825: hypothetical protein	-	-	-	-	-	-	-	-	-	-	-	+	+	-
Cluster_2656	hypothetical protein	-	-	-	-	-	-	-	-	-	-	-	+	-	-
Cluster_2661	hypothetical protein	-	-	-	-	-	-	-	-	-	+	-	-	-	-
Cluster_2682	Ornithine cyclodeaminase (EC 4.3.1.12)	-	-	-	-	-	-	-	-	-	-	-	+	+	-
Cluster_2684	Glycosyltransferase	-	-	-	-	-	-	-	-	-	-	-	-	+	-
Cluster_2687	Transcriptional regulator	-	-	-	-	-	-	-	-	-	-	+	-	-	-
Cluster_2689	hypothetical protein	-	-	-	-	-	-	-	-	-	+	-	-	-	-
Cluster_2695	FkbM family methyltransferase	-	-	-	-	-	-	-	-	-	+	-	-	-	-
Cluster_2708	COGs COG2378	-	-	-	-	-	-	-	-	-	-	+	-	-	-
Cluster_2724	Phage capsid protein #Fam0066	-	-	-	-	-	-	-	-	-	-	+	-	-	-
Cluster_2736	Transcriptional regulator, LysR family	-	-	-	-	-	-	-	-	-	-	-	-	-	+
Cluster_274	FIG131328: Predicted ATP-dependent endonuclease of the OLD family	-	-	-	-	-	-	-	-	-	-	-	-	+	-
Cluster_2743	Transcriptional regulator	-	-	-	-	-	-	-	-	-	-	-	+	+	-
Cluster_2744	FIG01202522: hypothetical protein	-	-	-	-	-	-	-	-	-	-	-	+	+	-
Cluster_275	Cellulose synthase catalytic subunit [UDP-forming] (EC 2.4.1.12)	-	-	-	-	-	-	-	-	-	-	-	+	+	-
Cluster_2762	hypothetical protein	-	-	-	-	-	-	-	-	-	-	-	+	+	-
Cluster_2780	Glycosyltransferase (EC 2.4.1.-)	-	-	-	-	-	-	-	-	-	-	-	+	-	-
Cluster_2786	hypothetical protein	-	-	-	-	-	-	-	-	-	-	-	+	+	-
Cluster_2799	hypothetical protein	-	-	-	-	-	-	-	-	-	-	+	-	-	-
Cluster_282	Cyclic di-GMP binding protein precursor	-	-	-	-	-	-	-	-	-	-	-	+	+	-
Cluster_2821	hypothetical protein	-	-	-	-	-	-	-	-	-	+	+	-	-	-
Cluster_2823	Glycosyltransferase	-	-	-	-	-	-	-	-	-	-	-	-	+	-
Cluster_2827	hypothetical protein	-	-	-	-	-	-	-	-	-	-	-	-	+	-
Cluster_2828	Transcriptional regulators, LysR family	-	-	-	-	-	-	-	-	-	-	-	+	+	-
Cluster_2829	Type II restriction enzyme BsuBI (EC 3.1.21.4)	-	-	-	-	-	-	-	-	-	-	-	-	+	-
Cluster_2839	hypothetical protein	-	-	-	-	-	-	-	-	-	-	-	-	-	+
Cluster_2842	Baseplate assembly protein J	-	-	-	-	-	-	-	-	-	-	-	-	+	-
Cluster_2849	Methionyl-tRNA formyltransferase (EC 2.1.2.9)	-	-	-	-	-	-	-	-	-	-	-	+	+	-
Cluster_2866	hypothetical protein	-	-	-	-	-	-	-	-	-	-	+	-	-	-
Cluster_2867	putative integrase	-	-	-	-	-	-	-	-	-	-	+	-	-	-
Cluster_2868	Glycosyltransferase (EC 2.4.1.-)	-	-	-	-	-	-	-	-	-	+	-	-	-	-
Cluster_2874	hypothetical protein	-	-	-	-	-	-	-	-	-	+	-	-	-	-
Cluster_2890	FIG01202833: hypothetical protein	-	-	-	-	-	-	-	-	-	-	-	-	-	+
Cluster_2896	hypothetical protein	-	-	-	-	-	-	-	-	-	-	+	-	-	-
Cluster_29	Long-chain-fatty-acid--CoA ligase (EC 6.2.1.3)	-	-	-	-	-	-	-	-	-	-	+	-	-	-
Cluster_2901	Glycosyltransferase(EC:2.4.1.-)	-	-	-	-	-	-	-	-	-	+	-	-	-	-
Cluster_291	hypothetical protein	-	-	-	-	-	-	-	-	-	-	-	-	-	+
Cluster_2911	hypothetical protein	-	-	-	-	-	-	-	-	-	-	+	-	-	-
Cluster_2923	hypothetical protein	-	-	-	-	-	-	-	-	-	-	-	+	+	-
Cluster_293	hypothetical protein	-	-	-	-	-	-	-	-	-	-	+	-	-	-
Cluster_2932	Permease of the drug/metabolite transporter (DMT) superfamily	-	-	-	-	-	-	-	-	-	+	-	-	-	-
Cluster_2935	hypothetical protein	-	-	-	-	-	-	-	-	-	-	-	-	-	+
Cluster_2938	hypothetical protein	-	-	-	-	-	-	-	-	-	-	-	-	-	+
Cluster_2944	hypothetical protein	-	-	-	-	-	-	-	-	-	-	-	-	+	-
Cluster_2951	hypothetical protein	-	-	-	-	-	-	-	-	-	-	+	-	-	-
Cluster_2963	hypothetical protein	-	-	-	-	-	-	-	-	-	-	-	-	+	-
Cluster_2968	hypothetical protein	-	-	-	-	-	-	-	-	-	-	-	+	-	+
Cluster_297	Putative insecticidal toxin complex	-	-	-	-	-	-	-	-	-	-	+	-	-	-
Cluster_3016	D-3-phosphoglycerate dehydrogenase (EC 1.1.1.95)	-	-	-	-	-	-	-	-	-	+	-	-	-	+
Cluster_3020	putative epoxide hydrolase	-	-	-	-	-	-	-	-	-	-	-	+	+	-
Cluster_3044	probable membrane protein YPO2863	-	-	-	-	-	-	-	-	-	-	-	-	+	+
Cluster_305	Translation-disabling ACNase RloC	-	-	-	-	-	-	-	-	-	-	-	-	-	+
Cluster_3088	Glycosyltransferase (EC 2.4.1.-)	-	-	-	-	-	-	-	-	-	-	-	-	+	-
Cluster_3092	hypothetical protein	-	-	-	-	-	-	-	-	-	-	-	+	-	-
Cluster_3119	Glycosyltransferase (EC 2.4.1.-)	-	-	-	-	-	-	-	-	-	-	-	+	-	-
Cluster_3132	FIG01202005: hypothetical protein	-	-	-	-	-	-	-	-	-	+	-	-	-	-
Cluster_3139	Maltose operon periplasmic protein MalM	-	-	-	-	-	-	-	-	-	-	-	-	-	+
Cluster_3140	hypothetical protein	-	-	-	-	-	-	-	-	-	-	-	-	+	-
Cluster_3153	Prophage MuSo2, transcriptional regulator, Cro/CI family	-	-	-	-	-	-	-	-	-	-	-	-	-	+
Cluster_3168	glycosyltransferase	-	-	-	-	-	-	-	-	-	+	-	-	-	-
Cluster_3181	hypothetical protein	-	-	-	-	-	-	-	-	-	-	-	+	-	-
Cluster_3182	conserved hypothetical protein	-	-	-	-	-	-	-	-	-	+	-	-	-	-
Cluster_3184	hypothetical protein	-	-	-	-	-	-	-	-	-	-	-	-	-	+
Cluster_3188	Homoserine kinase (EC 2.7.1.39)	-	-	-	-	-	-	-	-	-	-	-	-	-	+
Cluster_3201	Mobile element protein	-	-	-	-	-	-	-	-	-	-	+	-	-	-
Cluster_3203	hypothetical protein	-	-	-	-	-	-	-	-	-	+	-	-	-	-
Cluster_3206	hypothetical protein	-	-	-	-	-	-	-	-	-	-	-	-	+	-
Cluster_3217	hypothetical protein	-	-	-	-	-	-	-	-	-	-	-	+	+	-
Cluster_3218	hypothetical protein	-	-	-	-	-	-	-	-	-	-	+	-	-	-
Cluster_3231	hypothetical protein	-	-	-	-	-	-	-	-	-	-	+	-	-	-
Cluster_3239	Nitroreductase family protein	-	-	-	-	-	-	-	-	-	-	-	+	-	-
Cluster_3252	Mobile element protein	-	-	-	-	-	-	-	-	-	-	-	-	+	-
Cluster_3267	hypothetical protein	-	-	-	-	-	-	-	-	-	-	-	-	+	-
Cluster_3268	hypothetical protein	-	-	-	-	-	-	-	-	-	-	-	-	+	-

Cluster_3279	hypothetical protein	-	-	-	-	-	-	-	-	-	-	-	-	-	+
Cluster_3282	hypothetical protein	-	-	-	-	-	-	-	-	-	+	-	-	-	-
Cluster_3283	Glycosyl transferase, group 2 family protein	-	-	-	-	-	-	-	-	-	-	-	+	-	-
Cluster_3295	hypothetical protein	-	-	-	-	-	-	-	-	-	-	+	-	-	-
Cluster_3308	Type I restriction-modification system, specificity subunit S (EC 3.1.21.3)	-	-	-	-	-	-	-	-	-	+	-	-	-	-
Cluster_3310	hypothetical protein	-	-	-	-	-	-	-	-	-	-	-	-	+	-
Cluster_3328	hypothetical protein	-	-	-	-	-	-	-	-	-	-	+	-	-	-
Cluster_3335	Prophage MuSo2, transcriptional regulator, Cro/CI family	-	-	-	-	-	-	-	-	-	-	+	-	-	-
Cluster_3346	hypothetical protein	-	-	-	-	-	-	-	-	-	-	-	-	+	-
Cluster_3356	hypothetical protein	-	-	-	-	-	-	-	-	-	-	+	-	-	-
Cluster_3357	polysaccharide biosynthesis protein	-	-	-	-	-	-	-	-	-	+	-	-	-	-
Cluster_3359	DNA-invertase	-	-	-	-	-	-	-	-	-	-	-	-	-	+
Cluster_336	hypothetical protein	-	-	-	-	-	-	-	-	-	-	-	-	-	+
Cluster_3389	Cellulose synthase, putative	-	-	-	-	-	-	-	-	-	-	-	+	+	-
Cluster_3391	hypothetical protein	-	-	-	-	-	-	-	-	-	+	-	-	-	-
Cluster_3402	Putative predicted metal-dependent hydrolase	-	-	-	-	-	-	-	-	-	-	-	-	-	+
Cluster_3406	Conjugative transfer protein TrbJ	-	-	-	-	-	-	-	-	-	+	-	-	-	-
Cluster_3407	Transcriptional regulator, AraC family	-	-	-	-	-	-	-	-	-	+	-	-	-	-
Cluster_3420	FIG01206255: hypothetical protein	-	-	-	-	-	-	-	-	-	-	-	+	-	-
Cluster_3438	hypothetical protein	-	-	-	-	-	-	-	-	-	-	-	-	-	+
Cluster_3451	Putative N-acetylgalactosaminyl-diphosphoundecaprenol glucuronosyltransferase	-	-	-	-	-	-	-	-	-	-	-	+	+	-
Cluster_3452	Cellobiose phosphotransferase system YdjC-like protein	-	-	-	-	-	-	-	-	-	-	+	-	-	-
Cluster_3454	hypothetical protein	-	-	-	-	-	-	-	-	-	-	-	+	-	-
Cluster_346	ATP-dependent DNA helicase pcrA (EC 3.6.1.-)	-	-	-	-	-	-	-	-	-	-	-	-	+	-
Cluster_3467	hypothetical protein	-	-	-	-	-	-	-	-	-	+	-	-	-	-
Cluster_3469	Putative N-acetylgalactosaminyl-diphosphoundecaprenol glucuronosyltransferase	-	-	-	-	-	-	-	-	-	-	-	+	+	-
Cluster_3470	hypothetical protein	-	-	-	-	-	-	-	-	-	-	+	-	-	+
Cluster_3471	hypothetical protein	-	-	-	-	-	-	-	-	-	-	+	-	-	+
Cluster_3499	hypothetical protein	-	-	-	-	-	-	-	-	-	-	-	-	-	+
Cluster_3531	3-oxoacyl-[acyl-carrier protein] reductase (EC 1.1.1.100)	-	-	-	-	-	-	-	-	-	-	+	-	-	-
Cluster_3535	Mobile element protein	-	-	-	-	-	-	-	-	-	-	+	-	-	-
Cluster_3567	hypothetical protein	-	-	-	-	-	-	-	-	-	+	-	-	-	-
Cluster_3570	hypothetical protein	-	-	-	-	-	-	-	-	-	+	-	-	-	-
Cluster_3584	Mobile element protein	-	-	-	-	-	-	-	-	-	-	+	-	-	-
Cluster_3585	hypothetical protein	-	-	-	-	-	-	-	-	-	+	-	-	-	-
Cluster_3595	hypothetical protein	-	-	-	-	-	-	-	-	-	-	-	+	-	-
Cluster_3598	hypothetical protein	-	-	-	-	-	-	-	-	-	-	-	-	+	-
Cluster_360	FIG01200582: hypothetical protein	-	-	-	-	-	-	-	-	-	-	-	+	+	-
Cluster_3610	hypothetical protein	-	-	-	-	-	-	-	-	-	-	-	-	+	-
Cluster_3613	hypothetical protein	-	-	-	-	-	-	-	-	-	-	+	-	-	-
Cluster_3631	DNA replication protein DnaC	-	-	-	-	-	-	-	-	-	-	-	-	+	-
Cluster_3647	hypothetical protein	-	-	-	-	-	-	-	-	-	+	-	-	-	-
Cluster_3649	hypothetical protein	-	-	-	-	-	-	-	-	-	-	-	+	-	-
Cluster_366	hypothetical protein	-	-	-	-	-	-	-	-	-	-	-	-	-	+
Cluster_3674	hypothetical protein	-	-	-	-	-	-	-	-	-	-	-	+	-	-
Cluster_3676	hypothetical protein	-	-	-	-	-	-	-	-	-	-	+	-	-	-
Cluster_3693	hypothetical protein	-	-	-	-	-	-	-	-	-	+	-	-	-	-
Cluster_3700	hypothetical protein	-	-	-	-	-	-	-	-	-	-	+	-	-	-
Cluster_3713	hypothetical protein	-	-	-	-	-	-	-	-	-	-	-	-	-	+
Cluster_3720	Putative predicted metal-dependent hydrolase	-	-	-	-	-	-	-	-	-	-	-	+	-	-
Cluster_3721	hypothetical protein	-	-	-	-	-	-	-	-	-	+	-	-	-	-
Cluster_3723	Riboflavin synthase eubacterial/eukaryotic (EC 2.5.1.9)	-	-	-	-	-	-	-	-	-	+	-	-	-	-
Cluster_3732	N-methylhydantoinase A/acetone carboxylase, beta subunit	-	-	-	-	-	-	-	-	-	-	-	-	+	-
Cluster_3735	Transcriptional regulator, AraC family	-	-	-	-	-	-	-	-	-	+	-	-	-	-
Cluster_375	FIG01200582: hypothetical protein	-	-	-	-	-	-	-	-	-	+	-	+	+	-
Cluster_3767	hypothetical protein	-	-	-	-	-	-	-	-	-	+	-	-	-	-
Cluster_3774	DNA polymerase III epsilon subunit (EC 2.7.7.7)	-	-	-	-	-	-	-	-	-	+	-	-	-	+
Cluster_3778	type II restriction enzyme (Eco47II, Sau96I)	-	-	-	-	-	-	-	-	-	-	+	-	-	-
Cluster_378	hypothetical protein	-	-	-	-	-	-	-	-	-	-	+	-	-	-
Cluster_3783	protein of unknown function	-	-	-	-	-	-	-	-	-	+	-	-	-	-
Cluster_3803	Phage protein	-	-	-	-	-	-	-	-	-	-	-	-	+	-
Cluster_3808	COG3306: Glycosyltransferase involved in LPS biosynthesis	-	-	-	-	-	-	-	-	-	-	-	+	+	-
Cluster_3809	hypothetical protein	-	-	-	-	-	-	-	-	-	+	-	-	-	-
Cluster_3811	N-Acetylneuraminate cytidylyltransferase (EC 2.7.7.43)	-	-	-	-	-	-	-	-	-	-	-	+	+	-
Cluster_382	FIG131328: Predicted ATP-dependent endonuclease of the OLD family	-	-	-	-	-	-	-	-	-	+	-	-	-	-
Cluster_3824	hypothetical protein	-	-	-	-	-	-	-	-	-	+	-	-	-	-
Cluster_383	hypothetical protein	-	-	-	-	-	-	-	-	-	+	-	-	-	-
Cluster_3858	Putative fimbrial chaperone protein	-	-	-	-	-	-	-	-	-	-	+	-	-	-
Cluster_3872	2-haloalkanoic acid dehalogenase (EC 3.8.1.2)	-	-	-	-	-	-	-	-	-	-	-	-	-	+
Cluster_3882	hypothetical protein	-	-	-	-	-	-	-	-	-	-	+	-	-	-
Cluster_3891	hypothetical protein	-	-	-	-	-	-	-	-	-	-	+	-	-	-
Cluster_3892	hypothetical protein	-	-	-	-	-	-	-	-	-	+	-	-	-	-
Cluster_3901	hypothetical protein	-	-	-	-	-	-	-	-	-	-	-	-	-	+
Cluster_3902	FIG01202149: hypothetical protein	-	-	-	-	-	-	-	-	-	-	-	-	-	+
Cluster_3907	hypothetical protein	-	-	-	-	-	-	-	-	-	-	-	-	+	-
Cluster_3911	hypothetical protein	-	-	-	-	-	-	-	-	-	-	+	-	-	-
Cluster_3924	Mobile element protein	-	-	-	-	-	-	-	-	-	-	+	-	-	-
Cluster_3935	uncharacterized phage-encoded protein	-	-	-	-	-	-	-	-	-	-	-	-	+	-
Cluster_396	para-nitrobenzyl esterase (intracellular esterase B)	-	-	-	-	-	-	-	-	-	-	-	+	+	-
Cluster_3964	Positive regulator of competence TfoX	-	-	-	-	-	-	-	-	-	-	-	+	+	-
Cluster_3982	Phage repressor protein C2	-	-	-	-	-	-	-	-	-	-	-	-	+	-
Cluster_3995	Predicted HD superfamily hydrolase	-	-	-	-	-	-	-	-	-	-	+	-	-	-
Cluster_3997	FIG01205831: hypothetical protein	-	-	-	-	-	-	-	-	-	-	-	+	-	-
Cluster_4000	(adenine-N6-)-methyltransferase homolog	-	-	-	-	-	-	-	-	-	-	-	-	-	+
Cluster_4011	hypothetical protein	-	-	-	-	-	-	-	-	-	-	-	-	+	-
Cluster_4021	hypothetical protein	-	-	-	-	-	-	-	-	-	-	+	-	-	-
Cluster_4027	Putative threonine efflux protein	-	-	-	-	-	-	-	-	-	+	-	-	-	+
Cluster_4044	hypothetical protein	-	-	-	-	-	-	-	-	-	-	-	-	+	-
Cluster_406	Phage replication protein	-	-	-	-	-	-	-	-	-	-	-	+	+	-
Cluster_4061	hypothetical protein	-	-	-	-	-	-	-	-	-	-	-	-	+	-
Cluster_4070	hypothetical protein	-	-	-	-	-	-	-	-	-	-	+	-	-	-
Cluster_4072	hypothetical protein	-	-	-	-	-	-	-	-	-	+	-	-	-	-
Cluster_4075	hypothetical protein	-	-	-	-	-	-	-	-	-	+	-	-	-	-

Cluster_4078	hypothetical protein	-	-	-	-	-	-	-	-	-	-	+	-	-	-
Cluster_41	Cellulose synthase operon protein C	-	-	-	-	-	-	-	-	-	-	-	+	+	-
Cluster_4102	hypothetical protein	-	-	-	-	-	-	-	-	-	+	-	-	-	-
Cluster_411	hypothetical protein	-	-	-	-	-	-	-	-	-	-	-	+	-	-
Cluster_4113	Baseplate assembly protein V	-	-	-	-	-	-	-	-	-	-	-	-	+	-
Cluster_4115	hypothetical protein	-	-	-	-	-	-	-	-	-	-	-	-	-	+
Cluster_4134	hypothetical protein	-	-	-	-	-	-	-	-	-	-	-	-	-	+
Cluster_4138	FIG01204826: hypothetical protein	-	-	-	-	-	-	-	-	-	-	-	+	-	-
Cluster_4144	hypothetical protein	-	-	-	-	-	-	-	-	-	-	-	-	+	-
Cluster_4163	hypothetical protein	-	-	-	-	-	-	-	-	-	-	-	-	-	+
Cluster_4174	Tail protein I	-	-	-	-	-	-	-	-	-	-	-	-	+	-
Cluster_4177	hypothetical protein	-	-	-	-	-	-	-	-	-	-	-	+	-	-
Cluster_4182	hypothetical protein	-	-	-	-	-	-	-	-	-	+	-	-	-	-
Cluster_4195	hypothetical protein	-	-	-	-	-	-	-	-	-	-	+	-	-	-
Cluster_4201	hypothetical protein	-	-	-	-	-	-	-	-	-	+	-	-	-	-
Cluster_4202	hypothetical protein	-	-	-	-	-	-	-	-	-	-	-	-	-	+
Cluster_4203	hypothetical protein	-	-	-	-	-	-	-	-	-	-	-	-	-	+
Cluster_4213	hypothetical protein	-	-	-	-	-	-	-	-	-	-	+	-	-	-
Cluster_4219	Putative threonine efflux protein	-	-	-	-	-	-	-	-	-	+	-	-	-	+
Cluster_4220	COG3306: Glycosyltransferase involved in LPS biosynthesis	-	-	-	-	-	-	-	-	-	-	-	-	+	-
Cluster_4222	Repressor protein	-	-	-	-	-	-	-	-	-	-	-	-	+	-
Cluster_4231	Mobile element protein	-	-	-	-	-	-	-	-	-	-	-	+	-	-
Cluster_4232	Protein cII	-	-	-	-	-	-	-	-	-	-	+	-	-	-
Cluster_4233	hypothetical protein	-	-	-	-	-	-	-	-	-	-	-	-	-	+
Cluster_4242	hypothetical protein	-	-	-	-	-	-	-	-	-	-	-	-	+	-
Cluster_425	Phage tail fiber protein	-	-	-	-	-	-	-	-	-	-	-	-	+	-
Cluster_4263	hypothetical protein	-	-	-	-	-	-	-	-	-	-	-	-	+	-
Cluster_4265	hypothetical protein	-	-	-	-	-	-	-	-	-	-	-	-	-	+
Cluster_4266	hypothetical protein	-	-	-	-	-	-	-	-	-	-	+	-	-	-
Cluster_4284	hypothetical protein	-	-	-	-	-	-	-	-	-	+	-	-	-	-
Cluster_4306	hypothetical protein	-	-	-	-	-	-	-	-	-	-	+	-	-	-
Cluster_4307	hypothetical protein	-	-	-	-	-	-	-	-	-	-	+	-	-	-
Cluster_4322	hypothetical protein	-	-	-	-	-	-	-	-	-	-	-	+	-	-
Cluster_4328	hypothetical protein	-	-	-	-	-	-	-	-	-	-	-	+	-	-
Cluster_4357	hypothetical protein	-	-	-	-	-	-	-	-	-	-	-	-	+	-
Cluster_4360	hypothetical protein	-	-	-	-	-	-	-	-	-	-	-	-	+	-
Cluster_4369	Unknown	-	-	-	-	-	-	-	-	-	-	-	-	-	+
Cluster_437	hypothetical protein	-	-	-	-	-	-	-	-	-	-	+	-	-	-
Cluster_4377	Transcriptional regulator, TetR family	-	-	-	-	-	-	-	-	-	-	-	+	+	-
Cluster_4381	putative pancortin-3	-	-	-	-	-	-	-	-	-	+	-	-	-	-
Cluster_4388	Exodeoxyribonuclease VIII (EC 3.1.11.-)	-	-	-	-	-	-	-	-	-	-	-	-	-	+
Cluster_4394	hypothetical protein	-	-	-	-	-	-	-	-	-	-	-	-	-	+
Cluster_4414	hypothetical protein	-	-	-	-	-	-	-	-	-	-	-	-	+	-
Cluster_4435	hypothetical protein	-	-	-	-	-	-	-	-	-	-	-	-	-	+
Cluster_4439	FIG01204320: hypothetical protein	-	-	-	-	-	-	-	-	-	-	-	-	+	-
Cluster_4447	hypothetical protein	-	-	-	-	-	-	-	-	-	-	-	-	+	-
Cluster_4451	hypothetical protein	-	-	-	-	-	-	-	-	-	-	-	-	+	-
Cluster_4459	hypothetical protein	-	-	-	-	-	-	-	-	-	-	-	-	+	-
Cluster_446	hypothetical protein	-	-	-	-	-	-	-	-	-	-	+	-	-	-
Cluster_4467	Opacity protein-related protein	-	-	-	-	-	-	-	-	-	-	+	-	-	-
Cluster_4470	Tellurite resistance protein-related protein	-	-	-	-	-	-	-	-	-	-	-	-	+	-
Cluster_4480	putative hydrolase	-	-	-	-	-	-	-	-	-	-	+	-	-	-
Cluster_4502	hypothetical protein	-	-	-	-	-	-	-	-	-	-	-	+	-	-
Cluster_4510	hypothetical protein	-	-	-	-	-	-	-	-	-	-	-	-	+	-
Cluster_4523	hypothetical protein	-	-	-	-	-	-	-	-	-	+	-	-	-	-
Cluster_4524	FIG01199607: hypothetical protein	-	-	-	-	-	-	-	-	-	-	-	-	-	+
Cluster_453	Phage protein	-	-	-	-	-	-	-	-	-	-	-	-	+	-
Cluster_4532	hypothetical protein	-	-	-	-	-	-	-	-	-	-	-	-	-	+
Cluster_4539	hypothetical protein	-	-	-	-	-	-	-	-	-	-	-	-	+	-
Cluster_4545	Uncharacterized protein COG3236	-	-	-	-	-	-	-	-	-	+	-	-	-	-
Cluster_456	Phage Integrase	-	-	-	-	-	-	-	-	-	-	-	+	-	-
Cluster_4561	hypothetical protein	-	-	-	-	-	-	-	-	-	-	-	+	-	-
Cluster_4562	hypothetical protein	-	-	-	-	-	-	-	-	-	-	+	-	-	-
Cluster_4563	hypothetical protein	-	-	-	-	-	-	-	-	-	-	-	-	+	-
Cluster_4564	Resolvase	-	-	-	-	-	-	-	-	-	-	-	+	-	-
Cluster_4566	hypothetical protein	-	-	-	-	-	-	-	-	-	+	-	-	-	-
Cluster_4588	resolvase, putative	-	-	-	-	-	-	-	-	-	-	+	-	-	-
Cluster_46	hypothetical protein	-	-	-	-	-	-	-	-	-	-	+	-	-	-
Cluster_4607	hypothetical protein	-	-	-	-	-	-	-	-	-	-	-	-	+	-
Cluster_4608	hypothetical protein	-	-	-	-	-	-	-	-	-	-	-	-	-	+
Cluster_4613	hypothetical protein	-	-	-	-	-	-	-	-	-	-	+	-	-	-
Cluster_4614	hypothetical protein	-	-	-	-	-	-	-	-	-	-	-	-	-	+
Cluster_4616	Phage DNA invertase	-	-	-	-	-	-	-	-	-	-	-	+	-	-
Cluster_4632	Response regulator	-	-	-	-	-	-	-	-	-	-	+	-	-	-
Cluster_4635	GCN5-related N-acetyltransferase	-	-	-	-	-	-	-	-	-	+	-	-	-	+
Cluster_4654	Zn peptidase	-	-	-	-	-	-	-	-	-	-	+	-	-	-
Cluster_4686	Cold shock protein CspA	-	-	-	-	-	-	-	-	-	+	-	-	-	-
Cluster_4692	hypothetical protein	-	-	-	-	-	-	-	-	-	-	-	-	+	-
Cluster_4709	syc0482_c	-	-	-	-	-	-	-	-	-	-	-	+	-	-
Cluster_4734	hypothetical protein	-	-	-	-	-	-	-	-	-	-	-	-	+	-
Cluster_4736	hypothetical protein	-	-	-	-	-	-	-	-	-	+	-	-	-	-
Cluster_4741	FIG01217126: hypothetical protein	-	-	-	-	-	-	-	-	-	-	-	-	+	-
Cluster_4763	hypothetical protein	-	-	-	-	-	-	-	-	-	-	-	-	+	-
Cluster_4766	hypothetical protein	-	-	-	-	-	-	-	-	-	-	+	-	-	-
Cluster_4777	protein of unknown function UPF0157	-	-	-	-	-	-	-	-	-	+	-	-	-	-
Cluster_478	putative peptidase	-	-	-	-	-	-	-	-	-	-	-	+	+	-
Cluster_4799	hypothetical protein	-	-	-	-	-	-	-	-	-	-	-	+	-	-
Cluster_48	Putative superfamily I DNA helicases	-	-	-	-	-	-	-	-	-	-	-	-	-	+
Cluster_4820	Phage major tail tube protein	-	-	-	-	-	-	-	-	-	-	-	-	+	-
Cluster_4837	Mobile element protein	-	-	-	-	-	-	-	-	-	-	+	-	-	-
Cluster_4845	hypothetical protein	-	-	-	-	-	-	-	-	-	-	-	+	+	-
Cluster_4861	similar to ankyrin 2,3/unc44	-	-	-	-	-	-	-	-	-	-	-	+	+	-
Cluster_4862	hypothetical protein	-	-	-	-	-	-	-	-	-	-	-	+	-	-

Cluster_4887	hypothetical protein	-	-	-	-	-	-	-	-	-	-	+	-	-	-
Cluster_4892	hypothetical protein	-	-	-	-	-	-	-	-	-	-	-	-	+	-
Cluster_4895	hypothetical protein	-	-	-	-	-	-	-	-	-	+	-	-	-	-
Cluster_4897	hypothetical protein	-	-	-	-	-	-	-	-	-	-	-	-	-	+
Cluster_4907	hypothetical protein	-	-	-	-	-	-	-	-	-	-	-	-	+	-
Cluster_4910	no significant match	-	-	-	-	-	-	-	-	-	-	+	-	-	-
Cluster_4913	Predicted acetyltransferase	-	-	-	-	-	-	-	-	-	+	-	+	-	-
Cluster_4915	hypothetical protein	-	-	-	-	-	-	-	-	-	-	-	+	-	-
Cluster_492	ATP-dependent DNA helicase pcrA (EC 3.6.1.-)	-	-	-	-	-	-	-	-	-	+	-	-	-	-
Cluster_4931	FIG01206295: hypothetical protein	-	-	-	-	-	-	-	-	-	+	-	-	-	-
Cluster_4959	hypothetical protein	-	-	-	-	-	-	-	-	-	+	-	-	-	+
Cluster_496	hypothetical protein	-	-	-	-	-	-	-	-	-	-	-	+	-	-
Cluster_4968	Purine nucleoside phosphorylase (EC 2.4.2.1)	-	-	-	-	-	-	-	-	-	-	-	-	+	-
Cluster_4969	hypothetical protein	-	-	-	-	-	-	-	-	-	-	-	-	-	+
Cluster_497	Alkyl sulfatase or beta-lactamase (EC 3.1.6.-)	-	-	-	-	-	-	-	-	-	-	-	+	+	-
Cluster_4980	Histone acetyltransferase HPA2 and related acetyltransferases	-	-	-	-	-	-	-	-	-	-	-	-	+	-
Cluster_4993	putative cytoplasmic protein	-	-	-	-	-	-	-	-	-	+	-	-	-	-
Cluster_5012	MazG protein domain	-	-	-	-	-	-	-	-	-	+	-	-	-	+
Cluster_5013	hypothetical protein	-	-	-	-	-	-	-	-	-	-	+	-	-	-
Cluster_5014	Mobile element protein	-	-	-	-	-	-	-	-	-	-	+	-	-	-
Cluster_5029	thioredoxin-dependent thiol peroxidase	-	-	-	-	-	-	-	-	-	-	+	-	-	-
Cluster_5031	hypothetical protein	-	-	-	-	-	-	-	-	-	+	-	-	+	-
Cluster_5032	hypothetical protein	-	-	-	-	-	-	-	-	-	+	-	-	-	-
Cluster_5044	putative acetyltransferase	-	-	-	-	-	-	-	-	-	+	-	-	-	-
Cluster_5051	hypothetical protein	-	-	-	-	-	-	-	-	-	-	-	+	-	-
Cluster_5066	hypothetical protein	-	-	-	-	-	-	-	-	-	-	-	-	+	-
Cluster_5070	spermine/spermidine acetyltransferase	-	-	-	-	-	-	-	-	-	-	-	+	+	-
Cluster_5075	no significant match	-	-	-	-	-	-	-	-	-	-	-	-	+	-
Cluster_5076	UBA/THIF-type NAD/FAD binding protein	-	-	-	-	-	-	-	-	-	+	-	-	-	-
Cluster_5097	Predicted transcriptional regulator	-	-	-	-	-	-	-	-	-	+	-	-	-	-
Cluster_5098	hypothetical protein	-	-	-	-	-	-	-	-	-	-	+	-	-	-
Cluster_5100	hypothetical protein	-	-	-	-	-	-	-	-	-	-	-	-	+	-
Cluster_5104	hypothetical protein	-	-	-	-	-	-	-	-	-	+	-	-	-	-
Cluster_5112	hypothetical protein	-	-	-	-	-	-	-	-	-	-	-	-	-	+
Cluster_5115	hypothetical protein	-	-	-	-	-	-	-	-	-	-	-	-	+	-
Cluster_5119	hypothetical protein	-	-	-	-	-	-	-	-	-	-	-	-	+	-
Cluster_514	Phage replication protein	-	-	-	-	-	-	-	-	-	-	-	-	+	-
Cluster_5161	hypothetical protein	-	-	-	-	-	-	-	-	-	-	-	-	+	-
Cluster_5163	hypothetical protein	-	-	-	-	-	-	-	-	-	+	-	-	-	-
Cluster_5165	hypothetical protein	-	-	-	-	-	-	-	-	-	-	+	-	-	-
Cluster_5166	hypothetical protein	-	-	-	-	-	-	-	-	-	-	-	+	-	-
Cluster_5188	hypothetical protein	-	-	-	-	-	-	-	-	-	-	-	-	+	-
Cluster_5189	Phage external scaffolding protein #Protein D	-	-	-	-	-	-	-	-	-	-	-	+	-	-
Cluster_5204	hypothetical protein	-	-	-	-	-	-	-	-	-	+	-	-	-	-
Cluster_5205	hypothetical protein	-	-	-	-	-	-	-	-	-	-	-	-	+	+
Cluster_5212	Excinuclease ATPase subunit	-	-	-	-	-	-	-	-	-	+	-	-	-	-
Cluster_5225	conserved hypothetical protein; Bacteriophage P2 associated	-	-	-	-	-	-	-	-	-	-	-	-	-	+
Cluster_5226	putative DNA hydrolase	-	-	-	-	-	-	-	-	-	+	-	-	-	-
Cluster_5245	Probable transcription regulator	-	-	-	-	-	-	-	-	-	+	-	-	-	-
Cluster_5249	FIG01200735: hypothetical protein	-	-	-	-	-	-	-	-	-	-	-	-	+	-
Cluster_5250	GCN5-related N-acetyltransferase	-	-	-	-	-	-	-	-	-	-	-	-	+	-
Cluster_5256	hypothetical protein	-	-	-	-	-	-	-	-	-	+	+	-	-	-
Cluster_5264	hypothetical protein	-	-	-	-	-	-	-	-	-	+	+	-	-	-
Cluster_5268	Type IV pilin PilA	-	-	-	-	-	-	-	-	-	-	-	+	-	-
Cluster_5269	Histone acetyltransferase HPA2	-	-	-	-	-	-	-	-	-	-	-	-	-	+
Cluster_5280	hypothetical protein	-	-	-	-	-	-	-	-	-	-	+	-	-	-
Cluster_5316	DATP pyrophosphohydrolase (EC 3.6.1.-)	-	-	-	-	-	-	-	-	-	-	-	+	+	-
Cluster_5320	hypothetical protein	-	-	-	-	-	-	-	-	-	-	-	-	-	+
Cluster_5321	hypothetical protein	-	-	-	-	-	-	-	-	-	-	-	-	-	+
Cluster_5343	hypothetical protein	-	-	-	-	-	-	-	-	-	-	-	-	+	-
Cluster_5347	hypothetical protein	-	-	-	-	-	-	-	-	-	-	-	+	+	-
Cluster_5356	hypothetical protein	-	-	-	-	-	-	-	-	-	+	-	-	-	-
Cluster_5363	ATP-dependent DNA helicase UvrD/PcrA	-	-	-	-	-	-	-	-	-	-	+	-	-	-
Cluster_5367	FIG01201318: hypothetical protein	-	-	-	-	-	-	-	-	-	-	-	+	+	-
Cluster_5385	hypothetical protein	-	-	-	-	-	-	-	-	-	+	-	-	-	-
Cluster_5386	hypothetical protein	-	-	-	-	-	-	-	-	-	-	-	-	-	+
Cluster_5391	hypothetical protein	-	-	-	-	-	-	-	-	-	-	-	-	-	+
Cluster_5394	hypothetical protein	-	-	-	-	-	-	-	-	-	-	-	-	-	+
Cluster_5402	hypothetical protein	-	-	-	-	-	-	-	-	-	-	-	+	-	-
Cluster_5404	Probable acetyltransferase	-	-	-	-	-	-	-	-	-	+	-	+	-	+
Cluster_5410	hypothetical protein	-	-	-	-	-	-	-	-	-	+	-	-	-	-
Cluster_5412	hypothetical protein	-	-	-	-	-	-	-	-	-	-	+	-	-	-
Cluster_5413	hypothetical protein	-	-	-	-	-	-	-	-	-	-	+	-	-	-
Cluster_5414	hypothetical protein	-	-	-	-	-	-	-	-	-	-	-	+	-	-
Cluster_5419	hypothetical protein	-	-	-	-	-	-	-	-	-	+	-	-	-	-
Cluster_5422	hypothetical protein	-	-	-	-	-	-	-	-	-	-	+	-	-	-
Cluster_5425	hypothetical protein	-	-	-	-	-	-	-	-	-	+	-	-	-	-
Cluster_5428	hypothetical protein	-	-	-	-	-	-	-	-	-	-	-	-	+	-
Cluster_5429	glyoxalase family protein	-	-	-	-	-	-	-	-	-	+	-	-	-	-
Cluster_543	hypothetical protein	-	-	-	-	-	-	-	-	-	-	+	-	-	-
Cluster_5431	GCN5-related N-acetyltransferase	-	-	-	-	-	-	-	-	-	+	-	-	-	-
Cluster_5433	Glycosyltransferase (EC 2.4.1.-)	-	-	-	-	-	-	-	-	-	+	-	-	-	-
Cluster_5435	hypothetical protein	-	-	-	-	-	-	-	-	-	-	-	-	+	-
Cluster_5437	hypothetical protein	-	-	-	-	-	-	-	-	-	+	-	-	-	-
Cluster_5453	hypothetical protein	-	-	-	-	-	-	-	-	-	-	+	-	-	-
Cluster_5477	hypothetical protein	-	-	-	-	-	-	-	-	-	+	-	-	-	-
Cluster_5478	TolA protein	-	-	-	-	-	-	-	-	-	-	-	-	+	-
Cluster_5485	hypothetical protein	-	-	-	-	-	-	-	-	-	-	-	-	-	+
Cluster_5508	hypothetical protein	-	-	-	-	-	-	-	-	-	-	-	-	-	+
Cluster_5522	Error-prone repair protein UmuD	-	-	-	-	-	-	-	-	-	-	-	+	-	-
Cluster_5532	hypothetical protein	-	-	-	-	-	-	-	-	-	+	-	-	-	-
Cluster_5533	Phage protein	-	-	-	-	-	-	-	-	-	-	-	-	+	-
Cluster_5535	Phage tail protein	-	-	-	-	-	-	-	-	-	-	-	-	+	-

Cluster_5538	hypothetical protein	-	-	-	-	-	-	-	-	-	+	-	-	-	-
Cluster_5545	FIG01202219: hypothetical protein	-	-	-	-	-	-	-	-	-	-	-	+	+	-
Cluster_555	Outer membrane vitamin B12 receptor BtuB	-	-	-	-	-	-	-	-	-	+	-	-	-	+
Cluster_5551	FIG01202219: hypothetical protein	-	-	-	-	-	-	-	-	-	-	-	+	+	-
Cluster_5567	Probable acetyltransferase	-	-	-	-	-	-	-	-	-	+	-	+	-	+
Cluster_5568	hypothetical protein	-	-	-	-	-	-	-	-	-	+	-	-	-	-
Cluster_5573	hypothetical protein	-	-	-	-	-	-	-	-	-	-	-	-	+	-
Cluster_5577	Probable acetyltransferase	-	-	-	-	-	-	-	-	-	+	-	+	-	+
Cluster_5579	FIG01202219: hypothetical protein	-	-	-	-	-	-	-	-	-	-	-	+	+	-
Cluster_5598	hypothetical protein	-	-	-	-	-	-	-	-	-	-	-	-	-	+
Cluster_5603	hypothetical protein	-	-	-	-	-	-	-	-	-	-	-	+	-	-
Cluster_5609	hypothetical protein	-	-	-	-	-	-	-	-	-	-	-	-	+	-
Cluster_5612	Mobile element protein	-	-	-	-	-	-	-	-	-	-	-	-	+	-
Cluster_5648	hypothetical protein	-	-	-	-	-	-	-	-	-	+	-	-	-	-
Cluster_5695	hypothetical protein	-	-	-	-	-	-	-	-	-	-	-	-	+	-
Cluster_5715	hypothetical protein	-	-	-	-	-	-	-	-	-	-	+	-	-	-
Cluster_5718	hypothetical protein	-	-	-	-	-	-	-	-	-	-	+	-	-	-
Cluster_5729	hypothetical protein	-	-	-	-	-	-	-	-	-	-	-	-	-	+
Cluster_573	Phage terminase large subunit GpA	-	-	-	-	-	-	-	-	-	-	-	-	+	-
Cluster_574	hypothetical protein	-	-	-	-	-	-	-	-	-	-	+	-	-	-
Cluster_5745	hypothetical protein	-	-	-	-	-	-	-	-	-	-	-	-	+	-
Cluster_5749	hypothetical protein	-	-	-	-	-	-	-	-	-	+	-	-	-	-
Cluster_5753	hypothetical protein	-	-	-	-	-	-	-	-	-	+	-	-	-	-
Cluster_5757	hypothetical protein	-	-	-	-	-	-	-	-	-	-	-	+	-	-
Cluster_5762	hypothetical protein	-	-	-	-	-	-	-	-	-	+	-	+	-	-
Cluster_5765	hypothetical protein	-	-	-	-	-	-	-	-	-	-	-	-	-	+
Cluster_5770	hypothetical protein	-	-	-	-	-	-	-	-	-	-	-	-	-	+
Cluster_5788	FIG01201296: hypothetical protein	-	-	-	-	-	-	-	-	-	-	-	+	+	-
Cluster_5793	hypothetical protein	-	-	-	-	-	-	-	-	-	-	-	-	+	-
Cluster_5794	hypothetical protein	-	-	-	-	-	-	-	-	-	-	-	-	+	-
Cluster_5795	hypothetical protein	-	-	-	-	-	-	-	-	-	-	-	-	-	+
Cluster_58	Type I restriction-modification system, restriction subunit R (EC 3.1.21.3)	-	-	-	-	-	-	-	-	-	+	-	-	-	-
Cluster_5812	hypothetical protein	-	-	-	-	-	-	-	-	-	-	+	-	-	-
Cluster_5826	hypothetical protein	-	-	-	-	-	-	-	-	-	-	+	-	-	-
Cluster_5843	hypothetical protein	-	-	-	-	-	-	-	-	-	+	-	-	-	-
Cluster_5844	hypothetical protein	-	-	-	-	-	-	-	-	-	-	+	-	-	-
Cluster_5845	hypothetical protein	-	-	-	-	-	-	-	-	-	-	-	-	+	-
Cluster_5857	Phage protein	-	-	-	-	-	-	-	-	-	-	-	+	+	-
Cluster_5871	hypothetical protein	-	-	-	-	-	-	-	-	-	-	-	-	+	-
Cluster_5874	hypothetical protein	-	-	-	-	-	-	-	-	-	+	-	+	+	-
Cluster_5876	hypothetical protein	-	-	-	-	-	-	-	-	-	-	-	-	+	-
Cluster_5886	Transcriptional regulator, HxlR family	-	-	-	-	-	-	-	-	-	-	-	-	-	+
Cluster_5890	Phage baseplate assembly protein	-	-	-	-	-	-	-	-	-	-	-	-	+	-
Cluster_5893	hypothetical protein	-	-	-	-	-	-	-	-	-	-	+	-	-	-
Cluster_5894	hypothetical protein	-	-	-	-	-	-	-	-	-	-	-	-	+	-
Cluster_5914	hypothetical protein	-	-	-	-	-	-	-	-	-	+	-	-	-	-
Cluster_5925	hypothetical protein	-	-	-	-	-	-	-	-	-	-	+	-	-	-
Cluster_5927	hypothetical protein	-	-	-	-	-	-	-	-	-	-	+	-	-	-
Cluster_595	FIG01232333: hypothetical protein	-	-	-	-	-	-	-	-	-	-	-	+	-	-
Cluster_5950	hypothetical protein	-	-	-	-	-	-	-	-	-	+	-	-	-	-
Cluster_596	Phage tail protein	-	-	-	-	-	-	-	-	-	-	-	-	+	-
Cluster_5974	hypothetical protein	-	-	-	-	-	-	-	-	-	+	-	-	-	-
Cluster_5977	hypothetical protein	-	-	-	-	-	-	-	-	-	-	-	-	-	+
Cluster_5987	hypothetical protein	-	-	-	-	-	-	-	-	-	+	-	-	-	-
Cluster_599	Cytidine/deoxycytidylate deaminase family protein	-	-	-	-	-	-	-	-	-	-	-	+	-	-
Cluster_5999	hypothetical protein	-	-	-	-	-	-	-	-	-	-	+	-	-	-
Cluster_6000	hypothetical protein	-	-	-	-	-	-	-	-	-	-	+	-	-	-
Cluster_6016	hypothetical protein	-	-	-	-	-	-	-	-	-	-	+	-	-	-
Cluster_6020	Rep	-	-	-	-	-	-	-	-	-	-	+	-	-	-
Cluster_6022	FIG01202014: hypothetical protein	-	-	-	-	-	-	-	-	-	-	-	+	+	-
Cluster_6023	hypothetical protein	-	-	-	-	-	-	-	-	-	-	-	-	+	-
Cluster_6035	hypothetical protein	-	-	-	-	-	-	-	-	-	+	-	+	-	-
Cluster_6046	hypothetical protein	-	-	-	-	-	-	-	-	-	-	+	-	-	-
Cluster_6067	hypothetical protein	-	-	-	-	-	-	-	-	-	-	-	+	-	-
Cluster_6072	hypothetical protein	-	-	-	-	-	-	-	-	-	-	-	-	+	-
Cluster_6075	ParE toxin protein	-	-	-	-	-	-	-	-	-	+	-	-	-	+
Cluster_6078	probable hydrolase	-	-	-	-	-	-	-	-	-	-	+	-	-	-
Cluster_6079	hypothetical protein	-	-	-	-	-	-	-	-	-	-	-	-	+	-
Cluster_6099	FIG01202864: hypothetical protein	-	-	-	-	-	-	-	-	-	+	-	-	-	-
Cluster_6109	hypothetical protein	-	-	-	-	-	-	-	-	-	-	-	-	+	-
Cluster_611	RNA polymerase sigma factor RpoD	-	-	-	-	-	-	-	-	-	-	-	+	-	-
Cluster_6111	hypothetical protein	-	-	-	-	-	-	-	-	-	-	+	-	-	-
Cluster_6113	Phage major capsid protein	-	-	-	-	-	-	-	-	-	-	-	+	-	-
Cluster_6118	hypothetical protein	-	-	-	-	-	-	-	-	-	-	-	-	-	+
Cluster_6123	hypothetical protein	-	-	-	-	-	-	-	-	-	-	+	-	-	-
Cluster_615	FIG01200582: hypothetical protein	-	-	-	-	-	-	-	-	-	+	-	+	+	-
Cluster_6159	hypothetical protein	-	-	-	-	-	-	-	-	-	-	-	-	+	-
Cluster_6160	hypothetical protein	-	-	-	-	-	-	-	-	-	-	+	-	-	-
Cluster_6171	hypothetical protein	-	-	-	-	-	-	-	-	-	-	-	-	-	+
Cluster_6184	hypothetical protein	-	-	-	-	-	-	-	-	-	-	-	-	+	-
Cluster_6189	hypothetical protein	-	-	-	-	-	-	-	-	-	-	-	-	+	-
Cluster_62	Type I restriction-modification system, restriction subunit R (EC 3.1.21.3)	-	-	-	-	-	-	-	-	-	-	-	+	-	-
Cluster_6201	hypothetical protein	-	-	-	-	-	-	-	-	-	+	-	-	-	-
Cluster_6203	hypothetical protein	-	-	-	-	-	-	-	-	-	+	-	+	-	-
Cluster_6214	hypothetical protein	-	-	-	-	-	-	-	-	-	-	-	-	+	-
Cluster_6225	COG3311: Predicted transcriptional regulator	-	-	-	-	-	-	-	-	-	+	-	-	-	-
Cluster_6230	hypothetical protein	-	-	-	-	-	-	-	-	-	+	-	-	-	-
Cluster_6255	Mobile element protein	-	-	-	-	-	-	-	-	-	-	+	-	-	-
Cluster_6256	hypothetical protein	-	-	-	-	-	-	-	-	-	-	+	-	-	-
Cluster_6257	hypothetical protein	-	-	-	-	-	-	-	-	-	-	+	-	-	-
Cluster_6267	FIG01202262: hypothetical protein	-	-	-	-	-	-	-	-	-	-	-	+	+	-
Cluster_6270	Mobile element protein	-	-	-	-	-	-	-	-	-	-	+	-	-	-
Cluster_6272	hypothetical protein	-	-	-	-	-	-	-	-	-	-	-	+	-	-

Cluster_6274	hypothetical protein	-	-	-	-	-	-	-	-	-	+	-	-	-	-
Cluster_6289	hypothetical protein	-	-	-	-	-	-	-	-	-	-	+	-	-	-
Cluster_6298	hypothetical protein	-	-	-	-	-	-	-	-	-	-	-	+	+	-
Cluster_6299	hypothetical protein	-	-	-	-	-	-	-	-	-	-	-	+	+	-
Cluster_6301	hypothetical protein	-	-	-	-	-	-	-	-	-	+	-	-	-	-
Cluster_6305	hypothetical protein	-	-	-	-	-	-	-	-	-	-	-	+	+	-
Cluster_6311	Uncharacterized protein slr1025	-	-	-	-	-	-	-	-	-	-	-	+	-	-
Cluster_6318	hypothetical protein	-	-	-	-	-	-	-	-	-	+	-	-	-	-
Cluster_6323	putative acetyltransferase	-	-	-	-	-	-	-	-	-	+	-	-	-	-
Cluster_6330	hypothetical protein	-	-	-	-	-	-	-	-	-	-	-	-	-	+
Cluster_6334	hypothetical protein	-	-	-	-	-	-	-	-	-	-	-	-	-	+
Cluster_6344	hypothetical protein	-	-	-	-	-	-	-	-	-	-	-	-	+	-
Cluster_6345	hypothetical protein	-	-	-	-	-	-	-	-	-	-	-	+	-	-
Cluster_6348	hypothetical protein	-	-	-	-	-	-	-	-	-	-	-	+	+	-
Cluster_6364	hypothetical protein	-	-	-	-	-	-	-	-	-	-	-	+	+	-
Cluster_6371	hypothetical protein	-	-	-	-	-	-	-	-	-	+	-	-	-	-
Cluster_6372	hypothetical protein	-	-	-	-	-	-	-	-	-	-	-	-	+	-
Cluster_6380	ParD protein (antitoxin to ParE)	-	-	-	-	-	-	-	-	-	+	-	-	-	+
Cluster_6381	hypothetical protein	-	-	-	-	-	-	-	-	-	-	-	+	+	-
Cluster_6383	hypothetical protein	-	-	-	-	-	-	-	-	-	-	-	+	+	-
Cluster_6384	hypothetical protein	-	-	-	-	-	-	-	-	-	-	+	-	-	-
Cluster_6385	hypothetical protein	-	-	-	-	-	-	-	-	-	-	-	+	+	-
Cluster_6390	hypothetical protein	-	-	-	-	-	-	-	-	-	-	+	-	-	-
Cluster_6394	hypothetical protein	-	-	-	-	-	-	-	-	-	+	-	-	-	-
Cluster_64	Type I restriction-modification system, restriction subunit R (EC 3.1.21.3)	-	-	-	-	-	-	-	-	-	-	-	-	-	+
Cluster_6403	hypothetical protein	-	-	-	-	-	-	-	-	-	-	-	-	-	+
Cluster_6408	hypothetical protein	-	-	-	-	-	-	-	-	-	-	-	-	+	-
Cluster_6409	hypothetical protein	-	-	-	-	-	-	-	-	-	-	+	-	-	-
Cluster_6411	hypothetical protein	-	-	-	-	-	-	-	-	-	-	-	-	+	-
Cluster_6414	hypothetical protein	-	-	-	-	-	-	-	-	-	-	-	-	-	+
Cluster_6428	hypothetical protein	-	-	-	-	-	-	-	-	-	-	-	-	+	-
Cluster_6435	hypothetical protein	-	-	-	-	-	-	-	-	-	-	+	-	-	-
Cluster_6436	hypothetical protein	-	-	-	-	-	-	-	-	-	-	-	+	-	-
Cluster_6437	hypothetical protein	-	-	-	-	-	-	-	-	-	-	+	-	-	-
Cluster_6445	hypothetical protein	-	-	-	-	-	-	-	-	-	-	-	-	+	-
Cluster_6452	hypothetical protein	-	-	-	-	-	-	-	-	-	-	+	-	-	-
Cluster_6455	hypothetical protein	-	-	-	-	-	-	-	-	-	-	-	-	-	+
Cluster_6465	hypothetical protein	-	-	-	-	-	-	-	-	-	-	-	-	-	+
Cluster_6466	Phage protein	-	-	-	-	-	-	-	-	-	-	-	-	-	+
Cluster_6467	hypothetical protein	-	-	-	-	-	-	-	-	-	-	-	+	-	-
Cluster_6468	Phage capsid protein #Fam0066	-	-	-	-	-	-	-	-	-	-	+	-	-	-
Cluster_6472	Malonyl CoA-acyl carrier protein transacylase (EC 2.3.1.39)	-	-	-	-	-	-	-	-	-	-	-	-	+	-
Cluster_6475	hypothetical protein	-	-	-	-	-	-	-	-	-	-	-	-	-	+
Cluster_6496	hypothetical protein	-	-	-	-	-	-	-	-	-	-	+	-	-	-
Cluster_6502	hypothetical protein	-	-	-	-	-	-	-	-	-	-	-	-	-	+
Cluster_6504	hypothetical protein	-	-	-	-	-	-	-	-	-	-	+	-	-	-
Cluster_6508	Type I restriction-modification system, specificity subunit S (EC 3.1.21.3)	-	-	-	-	-	-	-	-	-	+	-	-	-	-
Cluster_6520	Msl2237 protein	-	-	-	-	-	-	-	-	-	-	-	+	+	-
Cluster_6528	HYPOTHETICAL/UNKNOWN PROTEIN	-	-	-	-	-	-	-	-	-	-	-	-	-	+
Cluster_6530	hypothetical protein	-	-	-	-	-	-	-	-	-	+	-	-	-	-
Cluster_6542	hypothetical protein	-	-	-	-	-	-	-	-	-	+	-	-	-	-
Cluster_6543	hypothetical protein	-	-	-	-	-	-	-	-	-	-	-	+	+	-
Cluster_6546	hypothetical protein	-	-	-	-	-	-	-	-	-	-	+	-	-	-
Cluster_6553	hypothetical protein	-	-	-	-	-	-	-	-	-	-	+	-	-	-
Cluster_6573	hypothetical protein	-	-	-	-	-	-	-	-	-	-	+	-	-	-
Cluster_6580	hypothetical protein	-	-	-	-	-	-	-	-	-	-	+	-	-	-
Cluster_6585	hypothetical protein	-	-	-	-	-	-	-	-	-	-	+	-	-	-
Cluster_6586	hypothetical protein	-	-	-	-	-	-	-	-	-	-	-	-	+	-
Cluster_661	hypothetical protein	-	-	-	-	-	-	-	-	-	-	-	+	-	-
Cluster_6610	hypothetical protein	-	-	-	-	-	-	-	-	-	+	-	-	-	-
Cluster_6613	hypothetical protein	-	-	-	-	-	-	-	-	-	+	-	+	-	-
Cluster_6616	hypothetical protein	-	-	-	-	-	-	-	-	-	-	-	-	-	+
Cluster_6618	probable phage tail protein	-	-	-	-	-	-	-	-	-	-	-	-	+	-
Cluster_6632	hypothetical protein	-	-	-	-	-	-	-	-	-	-	-	+	+	-
Cluster_6635	Mobile element protein	-	-	-	-	-	-	-	-	-	-	+	-	-	-
Cluster_6652	hypothetical protein	-	-	-	-	-	-	-	-	-	-	-	-	+	-
Cluster_6654	hypothetical protein	-	-	-	-	-	-	-	-	-	-	-	+	-	-
Cluster_6657	hypothetical protein	-	-	-	-	-	-	-	-	-	-	+	-	-	-
Cluster_6659	Conjugative transfer protein TrbL	-	-	-	-	-	-	-	-	-	+	-	-	-	-
Cluster_6673	hypothetical protein	-	-	-	-	-	-	-	-	-	-	-	-	-	+
Cluster_6676	FIG01201495: hypothetical protein	-	-	-	-	-	-	-	-	-	-	-	+	+	-
Cluster_6679	hypothetical protein	-	-	-	-	-	-	-	-	-	-	-	-	+	-
Cluster_6684	hypothetical protein	-	-	-	-	-	-	-	-	-	-	+	-	-	-
Cluster_6702	Msl2237 protein	-	-	-	-	-	-	-	-	-	-	-	+	+	-
Cluster_6717	hypothetical protein	-	-	-	-	-	-	-	-	-	-	-	-	-	+
Cluster_6718	hypothetical protein	-	-	-	-	-	-	-	-	-	-	-	-	+	-
Cluster_673	putative transport protein	-	-	-	-	-	-	-	-	-	-	-	+	+	-
Cluster_6730	Error-prone, lesion bypass DNA polymerase V (UmuC)	-	-	-	-	-	-	-	-	-	-	-	+	-	-
Cluster_6734	putative acetyltransferase	-	-	-	-	-	-	-	-	-	+	-	-	-	-
Cluster_6736	hypothetical protein	-	-	-	-	-	-	-	-	-	-	+	-	-	-
Cluster_6738	hypothetical protein	-	-	-	-	-	-	-	-	-	-	-	+	-	-
Cluster_674	hypothetical protein	-	-	-	-	-	-	-	-	-	+	-	-	-	-
Cluster_6744	hypothetical protein	-	-	-	-	-	-	-	-	-	-	-	-	+	-
Cluster_6748	hypothetical protein	-	-	-	-	-	-	-	-	-	-	+	-	-	-
Cluster_6764	SAM-dependent methyltransferase	-	-	-	-	-	-	-	-	-	+	-	-	-	-
Cluster_6765	FIG01205740: hypothetical protein	-	-	-	-	-	-	-	-	-	-	-	+	+	-
Cluster_6766	hypothetical protein	-	-	-	-	-	-	-	-	-	+	-	-	-	-
Cluster_6768	hypothetical protein	-	-	-	-	-	-	-	-	-	-	-	-	+	-
Cluster_678	hypothetical protein	-	-	-	-	-	-	-	-	-	-	+	-	-	-
Cluster_6783	hypothetical protein	-	-	-	-	-	-	-	-	-	-	-	-	+	-
Cluster_6808	hypothetical protein	-	-	-	-	-	-	-	-	-	-	-	-	+	-
Cluster_6812	hypothetical protein	-	-	-	-	-	-	-	-	-	+	-	-	-	-
Cluster_6821	hypothetical protein	-	-	-	-	-	-	-	-	-	-	+	-	-	-

Cluster_6822	Mobile element protein	-	-	-	-	-	-	-	-	-	-	+	-	-	-
Cluster_6823	FIG01200664: hypothetical protein	-	-	-	-	-	-	-	-	-	-	-	+	+	-
Cluster_6843	no significant match	-	-	-	-	-	-	-	-	-	-	+	-	-	-
Cluster_6844	hypothetical protein	-	-	-	-	-	-	-	-	-	-	-	-	+	-
Cluster_6845	hypothetical protein	-	-	-	-	-	-	-	-	-	-	+	-	-	-
Cluster_6846	no significant match	-	-	-	-	-	-	-	-	-	+	-	-	-	-
Cluster_6850	hypothetical protein	-	-	-	-	-	-	-	-	-	-	+	-	-	-
Cluster_6854	hypothetical protein	-	-	-	-	-	-	-	-	-	-	+	-	-	-
Cluster_6859	hypothetical protein	-	-	-	-	-	-	-	-	-	-	+	-	-	-
Cluster_6885	hypothetical protein	-	-	-	-	-	-	-	-	-	+	-	-	-	-
Cluster_6889	hypothetical protein	-	-	-	-	-	-	-	-	-	-	-	-	-	+
Cluster_6899	hypothetical protein	-	-	-	-	-	-	-	-	-	-	+	-	-	-
Cluster_690	FIG131328: Predicted ATP-dependent endonuclease of the OLD family	-	-	-	-	-	-	-	-	-	-	-	+	+	-
Cluster_6915	hypothetical protein	-	-	-	-	-	-	-	-	-	-	-	-	+	-
Cluster_6917	hypothetical protein	-	-	-	-	-	-	-	-	-	+	-	-	-	-
Cluster_694	hypothetical protein	-	-	-	-	-	-	-	-	-	-	+	-	-	-
Cluster_6940	hypothetical protein	-	-	-	-	-	-	-	-	-	-	-	+	+	-
Cluster_6975	hypothetical protein	-	-	-	-	-	-	-	-	-	-	-	-	+	-
Cluster_6984	no significant match	-	-	-	-	-	-	-	-	-	-	+	-	-	-
Cluster_6992	Rhs family protein	-	-	-	-	-	-	-	-	-	-	-	+	+	-
Cluster_6995	hypothetical protein	-	-	-	-	-	-	-	-	-	-	-	+	+	-
Cluster_7002	hypothetical protein	-	-	-	-	-	-	-	-	-	-	-	+	-	-
Cluster_7005	hypothetical protein	-	-	-	-	-	-	-	-	-	-	-	-	+	-
Cluster_7013	Mll3428 protein	-	-	-	-	-	-	-	-	-	-	-	-	+	-
Cluster_705	2-isopropylmalate synthase (EC 2.3.3.13)	-	-	-	-	-	-	-	-	-	+	-	-	-	-
Cluster_7054	hypothetical protein	-	-	-	-	-	-	-	-	-	-	-	-	+	-
Cluster_7057	hypothetical protein	-	-	-	-	-	-	-	-	-	-	+	-	-	-
Cluster_7069	hypothetical protein	-	-	-	-	-	-	-	-	-	+	-	-	-	-
Cluster_7080	ISSod21 transposase, pseudogene # TnpA_ISSod21	-	-	-	-	-	-	-	-	-	-	+	-	-	-
Cluster_7090	hypothetical protein	-	-	-	-	-	-	-	-	-	-	+	-	-	-
Cluster_7107	hypothetical protein	-	-	-	-	-	-	-	-	-	+	-	-	-	-
Cluster_711	Type I restriction-modification system, DNA-methyltransferase subunit M (EC 2.1.1.72)	-	-	-	-	-	-	-	-	-	+	-	-	-	-
Cluster_7116	hypothetical protein	-	-	-	-	-	-	-	-	-	+	-	-	-	-
Cluster_7118	hypothetical protein	-	-	-	-	-	-	-	-	-	-	+	-	-	-
Cluster_7129	hypothetical protein	-	-	-	-	-	-	-	-	-	-	+	-	-	-
Cluster_7131	hypothetical protein	-	-	-	-	-	-	-	-	-	-	-	-	+	-
Cluster_7135	hypothetical protein	-	-	-	-	-	-	-	-	-	-	-	+	+	-
Cluster_7154	hypothetical protein	-	-	-	-	-	-	-	-	-	+	-	-	-	-
Cluster_7174	no significant match	-	-	-	-	-	-	-	-	-	+	-	-	-	-
Cluster_7175	hypothetical protein	-	-	-	-	-	-	-	-	-	-	-	+	+	-
Cluster_7178	hypothetical protein	-	-	-	-	-	-	-	-	-	-	-	+	-	-
Cluster_7189	hypothetical protein	-	-	-	-	-	-	-	-	-	-	-	-	+	-
Cluster_7194	hypothetical protein	-	-	-	-	-	-	-	-	-	+	-	+	-	-
Cluster_7206	hypothetical protein	-	-	-	-	-	-	-	-	-	+	-	-	-	-
Cluster_722	DNA-cytosine methyltransferase (EC 2.1.1.37)	-	-	-	-	-	-	-	-	-	-	-	+	-	-
Cluster_7234	hypothetical protein	-	-	-	-	-	-	-	-	-	-	-	+	-	-
Cluster_7237	no significant match	-	-	-	-	-	-	-	-	-	-	-	-	+	-
Cluster_7245	rolling circle replication protein, Rep63 protein	-	-	-	-	-	-	-	-	-	-	+	-	-	-
Cluster_7247	hypothetical protein	-	-	-	-	-	-	-	-	-	-	-	-	+	-
Cluster_7266	no significant match	-	-	-	-	-	-	-	-	-	-	-	+	+	-
Cluster_7274	hypothetical protein	-	-	-	-	-	-	-	-	-	-	+	-	-	-
Cluster_7276	hypothetical protein	-	-	-	-	-	-	-	-	-	-	+	-	-	-
Cluster_7288	Mobile element protein	-	-	-	-	-	-	-	-	-	-	-	-	+	-
Cluster_7300	Mobile element protein	-	-	-	-	-	-	-	-	-	-	-	+	+	-
Cluster_7317	hypothetical protein	-	-	-	-	-	-	-	-	-	-	-	+	+	+
Cluster_7319	hypothetical protein	-	-	-	-	-	-	-	-	-	-	+	-	-	-
Cluster_7326	hypothetical protein	-	-	-	-	-	-	-	-	-	-	+	-	-	-
Cluster_7334	hypothetical protein	-	-	-	-	-	-	-	-	-	-	+	-	-	-
Cluster_7335	hypothetical protein	-	-	-	-	-	-	-	-	-	-	-	-	+	-
Cluster_7343	hypothetical protein	-	-	-	-	-	-	-	-	-	-	-	+	+	-
Cluster_7351	hypothetical protein	-	-	-	-	-	-	-	-	-	-	-	-	+	-
Cluster_7353	hypothetical protein	-	-	-	-	-	-	-	-	-	+	-	-	-	-
Cluster_7361	hypothetical protein	-	-	-	-	-	-	-	-	-	-	+	-	-	-
Cluster_7387	hypothetical protein	-	-	-	-	-	-	-	-	-	+	-	-	-	-
Cluster_7389	hypothetical protein	-	-	-	-	-	-	-	-	-	+	-	-	-	+
Cluster_7402	hypothetical protein	-	-	-	-	-	-	-	-	-	+	-	-	-	-
Cluster_7421	Rep	-	-	-	-	-	-	-	-	-	-	+	-	-	-
Cluster_743	Phage T7 exclusion protein	-	-	-	-	-	-	-	-	-	-	+	-	-	-
Cluster_744	FIG01205715: hypothetical protein	-	-	-	-	-	-	-	-	-	-	-	+	+	-
Cluster_7468	hypothetical protein	-	-	-	-	-	-	-	-	-	-	-	+	+	-
Cluster_7469	hypothetical protein	-	-	-	-	-	-	-	-	-	-	+	-	-	-
Cluster_7518	hypothetical protein	-	-	-	-	-	-	-	-	-	-	-	-	+	-
Cluster_7537	hypothetical protein	-	-	-	-	-	-	-	-	-	-	-	-	+	-
Cluster_7539	hypothetical protein	-	-	-	-	-	-	-	-	-	-	-	-	-	+
Cluster_7546	hypothetical protein	-	-	-	-	-	-	-	-	-	-	-	-	+	-
Cluster_7563	FIG01205007: hypothetical protein	-	-	-	-	-	-	-	-	-	-	-	+	+	-
Cluster_7588	hypothetical protein	-	-	-	-	-	-	-	-	-	-	-	+	+	-
Cluster_7592	hypothetical protein	-	-	-	-	-	-	-	-	-	-	+	-	-	-
Cluster_7595	hypothetical protein	-	-	-	-	-	-	-	-	-	-	-	-	-	+
Cluster_7621	hypothetical protein	-	-	-	-	-	-	-	-	-	-	+	-	-	-
Cluster_7641	hypothetical protein	-	-	-	-	-	-	-	-	-	-	-	+	-	-
Cluster_7654	Phage DNA binding protein	-	-	-	-	-	-	-	-	-	-	-	+	-	-
Cluster_7659	hypothetical protein	-	-	-	-	-	-	-	-	-	-	-	+	+	-
Cluster_768	Predicted sodium-dependent galactose transporter	-	-	-	-	-	-	-	-	-	+	-	-	-	+
Cluster_7684	Dolichol-phosphate mannosyltransferase (EC 2.4.1.83) in lipid-linked oligosaccharide synthesis cluster	-	-	-	-	-	-	-	-	-	-	-	-	+	-
Cluster_7727	hypothetical protein	-	-	-	-	-	-	-	-	-	-	+	-	-	-
Cluster_7737	hypothetical protein	-	-	-	-	-	-	-	-	-	-	+	-	-	-
Cluster_7739	hypothetical protein	-	-	-	-	-	-	-	-	-	-	-	-	+	-
Cluster_7748	hypothetical protein	-	-	-	-	-	-	-	-	-	+	-	-	-	-
Cluster_7760	hypothetical protein	-	-	-	-	-	-	-	-	-	-	+	-	-	-
Cluster_7766	hypothetical protein	-	-	-	-	-	-	-	-	-	+	-	-	-	-
Cluster_7769	Conjugative transfer protein TrbL	-	-	-	-	-	-	-	-	-	+	-	-	-	-
Cluster_7778	hypothetical protein	-	-	-	-	-	-	-	-	-	-	-	-	-	+

Cluster_7782	hypothetical protein	-	-	-	-	-	-	-	-	-	-	-	-	-	+
Cluster_780	FIG002337: predicted inner membrane protein	-	-	-	-	-	-	-	-	-	-	-	+	+	-
Cluster_8	hypothetical protein	-	-	-	-	-	-	-	-	-	-	-	-	-	+
Cluster_805	L-aspartate-beta-decarboxylase	-	-	-	-	-	-	-	-	-	-	-	+	+	-
Cluster_816	hypothetical protein	-	-	-	-	-	-	-	-	-	-	+	-	-	-
Cluster_827	FIG005274: hypothetical protein	-	-	-	-	-	-	-	-	-	-	-	+	+	-
Cluster_830	hypothetical protein	-	-	-	-	-	-	-	-	-	-	-	-	-	+
Cluster_857	Phage-related portal protein	-	-	-	-	-	-	-	-	-	-	-	-	+	-
Cluster_865	hypothetical protein	-	-	-	-	-	-	-	-	-	-	+	-	-	-
Cluster_888	Phage DNA replication protein	-	-	-	-	-	-	-	-	-	-	-	+	-	-
Cluster_898	Mobile element protein	-	-	-	-	-	-	-	-	-	-	+	-	-	-
Cluster_906	Recombinase	-	-	-	-	-	-	-	-	-	-	-	+	-	-
Cluster_920	Modification methylase PstI (EC 2.1.1.72)	-	-	-	-	-	-	-	-	-	-	-	-	+	-
Cluster_929	hypothetical protein	-	-	-	-	-	-	-	-	-	-	+	-	-	-
Cluster_937	hypothetical protein	-	-	-	-	-	-	-	-	-	-	-	-	-	+
Cluster_941	hypothetical protein	-	-	-	-	-	-	-	-	-	-	-	-	-	+
Cluster_98	Putative insecticidal toxin complex	-	-	-	-	-	-	-	-	-	-	+	-	-	-
Cluster_985	hypothetical protein	-	-	-	-	-	-	-	-	-	-	-	-	+	-
Cluster_990	FIG00715517: hypothetical protein	-	-	-	-	-	-	-	-	-	-	-	+	-	-
Cluster_998	Phage tail sheath monomer	-	-	-	-	-	-	-	-	-	-	-	-	+	-

Supplemental Table 3. Predicted protein function of LS-BSR gene clusters that are unique to individual genomes sequenced in this study

LS-BSR Cluster ID	Predicted Protein Function
**In K1461 only and no other genomes[a]**	
Cluster_2936	Potassium efflux system KefA protein / Small-conductance mechanosensitive channel
Cluster_7365	Glycine cleavage system transcriptional antiactivator GcvR
Cluster_4004	Lipid A core-O-antigen ligase
Cluster_3697	Acriflavin resistance protein
Cluster_4221	Long-chain-fatty-acid--CoA ligase (EC 6.2.1.3)
Cluster_1999	Cytochrome c-type biogenesis protein CcmD, interacts with CcmCE
Cluster_4567	COG4325: Predicted membrane protein
Cluster_5932	hypothetical protein
Cluster_5480	hypothetical protein
Cluster_6229	ABC-type transport system, involved in lipoprotein release, permease component
Cluster_4681	Multidrug resistance protein D
Cluster_254	Nucleotide sugar epimerase
Cluster_6393	Putative transport protein
Cluster_5079	Chromosome segregation ATPase
Cluster_5649	Ubiquinone biosynthesis monooxygenase UbiB
Cluster_4735	putative hemagglutinin/hemolysin-related protein
Cluster_4450	Osmosensitive K+ channel histidine kinase KdpD (EC 2.7.3.-)
Cluster_44	hypothetical protein
Cluster_3752	hypothetical protein
Cluster_5953	integrase, phage family
**In K1275 only and no other genomes[a]**	
Cluster_7223	hypothetical protein
Cluster_3408	hypothetical protein
Cluster_2909	putative transcriptional regulator
Cluster_6252	hypothetical protein
Cluster_7597	hypothetical protein
Cluster_992	hypothetical protein
Cluster_3888	FIG01202005: hypothetical protein
Cluster_1427	hypothetical protein
Cluster_1531	hypothetical protein
Cluster_1755	hypothetical protein
Cluster_6800	hypothetical protein
Cluster_2925	sensory box protein
Cluster_3946	ThiJ/PfpI family protein
Cluster_6614	hypothetical protein
Cluster_515	hypothetical protein
Cluster_818	L-lactate permease
Cluster_1271	Signal transduction histidine kinase
Cluster_3806	Two-component system response regulator QseB
Cluster_4438	Outer membrane protein
Cluster_7037	hypothetical protein
Cluster_6448	hypothetical protein
Cluster_2747	hypothetical protein
Cluster_1549	hypothetical protein
Cluster_5936	transcriptional regulator, XRE family
Cluster_5338	hypothetical protein
Cluster_3925	hypothetical protein
Cluster_5487	hypothetical protein
Cluster_3983	hypothetical protein
Cluster_4025	hypothetical protein
Cluster_1650	hypothetical protein
Cluster_5970	hypothetical protein
Cluster_1019	hypothetical protein

Cluster_3204	hypothetical protein
Cluster_4850	FIG01206151: hypothetical protein
Cluster_5523	hypothetical protein
Cluster_5684	hypothetical protein
Cluster_6841	hypothetical protein
Cluster_1272	hypothetical protein
Cluster_5553	hypothetical protein
Cluster_5432	hypothetical protein
Cluster_3021	hypothetical protein
Cluster_4161	hypothetical protein
Cluster_5358	hypothetical protein
Cluster_5067	hypothetical protein
Cluster_3380	hypothetical protein
Cluster_6401	hypothetical protein
Cluster_5846	hypothetical protein
Cluster_5524	NTP pyrophosphohydrolase
Cluster_5534	hypothetical protein
Cluster_2324	regulatory protein, LysR:LysR, substrate-binding
Cluster_3159	hypothetical protein
Cluster_5958	hypothetical protein
Cluster_1155	hypothetical protein
Cluster_5113	hypothetical protein
Cluster_4882	hypothetical protein
Cluster_5028	CI
Cluster_5902	FIG01204896: hypothetical protein
Cluster_4542	orf4
Cluster_4653	COG0030: Dimethyladenosine transferase (rRNA methylation)
Cluster_6412	hypothetical protein
Cluster_5337	hypothetical protein
Cluster_197	Phage-related tail fiber protein
Cluster_6290	hypothetical protein
Cluster_6786	hypothetical protein
Cluster_6878	hypothetical protein
Cluster_3790	hypothetical protein
Cluster_5355	hypothetical protein
Cluster_2106	hypothetical protein
Cluster_5570	hypothetical protein
Cluster_5190	hypothetical protein
Cluster_5710	MaoC-like dehydratase
Cluster_2997	transferase
Cluster_1622	Predicted membrane protein
Cluster_3169	hypothetical protein
Cluster_788	ABC-type transporter ATP-binding protein
Cluster_3468	hypothetical protein
Cluster_3486	hypothetical protein
Cluster_3253	hypothetical protein
Cluster_1782	hypothetical protein
Cluster_5702	hypothetical protein
Cluster_4637	hypothetical protein
Cluster_5663	hypothetical protein
Cluster_1670	FIG01205001: hypothetical protein
Cluster_3202	hypothetical protein
Cluster_897	hypothetical protein
Cluster_78	hypothetical protein
Cluster_451	IncF plasmid conjugative transfer protein TraD
Cluster_6351	hypothetical protein
Cluster_1464	hypothetical protein
Cluster_5046	hypothetical protein
Cluster_3736	replication P family protein
Cluster_6350	hypothetical protein

Cluster_4711	hypothetical protein
Cluster_6427	hypothetical protein
Cluster_4436	hypothetical protein
Cluster_5700	hypothetical protein
Cluster_358	hypothetical protein
Cluster_139	RecD-like DNA helicase YrrC
Cluster_4380	ThiJ/PfpI family protein
Cluster_5444	hypothetical protein
Cluster_3518	hypothetical protein
Cluster_5988	hypothetical protein
Cluster_6703	ABC-type maltose transport system, permease component
Cluster_2071	hypothetical protein
Cluster_6521	hypothetical protein
Cluster_5142	hypothetical protein
Cluster_3205	hypothetical protein
Cluster_4042	hypothetical protein
Cluster_4985	hypothetical protein
Cluster_3178	hypothetical protein
Cluster_4778	hypothetical protein
Cluster_7596	hypothetical protein
Cluster_2147	hypothetical protein
Cluster_2603	hypothetical protein
Cluster_3059	hypothetical protein
Cluster_5572	hypothetical protein
Cluster_3840	hypothetical protein
Cluster_6790	hypothetical protein
Cluster_3324	putative cytoplasmic protein
Cluster_2647	hypothetical protein
Cluster_5153	Putative translation initiation inhibitor, yjgF family
Cluster_4405	hypothetical protein
Cluster_5525	hypothetical protein
Cluster_6362	hypothetical protein
Cluster_3612	hypothetical protein
Cluster_2246	hypothetical protein
Cluster_5160	hypothetical protein
Cluster_6097	hypothetical protein
Cluster_205	hypothetical protein
Cluster_2573	hypothetical protein
Cluster_3867	hypothetical protein
Cluster_1830	DNA polymerase I - 3'-5' exonuclease and polymerase domains-like
Cluster_938	hypothetical protein
Cluster_725	hypothetical protein
Cluster_6500	CopG-like DNA-binding protein
Cluster_6215	RelE/StbE replicon stabilization toxin
Cluster_1724	phage integrase family protein
Cluster_5774	hypothetical protein
Cluster_3632	hypothetical protein
Cluster_5455	hypothetical protein
Cluster_7171	hypothetical protein
Cluster_5305	hypothetical protein
Cluster_6751	hypothetical protein
Cluster_234	conserved hypothetical protein
Cluster_4590	hypothetical protein
Cluster_6876	hypothetical protein
Cluster_4823	hypothetical protein
Cluster_2280	Mrr restriction protein-related
Cluster_7770	DNA ligase (EC 6.5.1.2)
Cluster_5290	Uncharacterized protein ImpI/VasC
**In AF91 only and no other genomes[a]**	

Cluster_6657	hypothetical protein
Cluster_5029	thioredoxin-dependent thiol peroxidase
Cluster_3676	hypothetical protein
Cluster_6496	hypothetical protein
Cluster_6546	hypothetical protein
Cluster_5826	hypothetical protein
Cluster_6850	hypothetical protein
Cluster_2896	hypothetical protein
Cluster_865	hypothetical protein
Cluster_5999	hypothetical protein
Cluster_6585	hypothetical protein
Cluster_4266	hypothetical protein
Cluster_3328	hypothetical protein
Cluster_2622	hypothetical protein
Cluster_3231	hypothetical protein
Cluster_4021	hypothetical protein
Cluster_1320	hypothetical protein
Cluster_6289	hypothetical protein
Cluster_4632	Response regulator
Cluster_6046	hypothetical protein
Cluster_6684	hypothetical protein
Cluster_2497	Phenazine biosynthesis protein PhzF like
Cluster_3531	3-oxoacyl-[acyl-carrier protein
Cluster_2687	Transcriptional regulator
Cluster_3452	Cellobiose phosphotransferase system YdjC-like protein
Cluster_6078	probable hydrolase
Cluster_4480	putative hydrolase
Cluster_1769	putative sugar transport protein
Cluster_6580	hypothetical protein
Cluster_2446	hypothetical protein
Cluster_2241	Mobile element protein
Cluster_7276	hypothetical protein
Cluster_3295	hypothetical protein
Cluster_6255	Mobile element protein
Cluster_7760	hypothetical protein
Cluster_6635	Mobile element protein
Cluster_4837	Mobile element protein
Cluster_5413	hypothetical protein
Cluster_4213	hypothetical protein
Cluster_5363	ATP-dependent DNA helicase UvrD/PcrA
Cluster_5422	hypothetical protein
Cluster_6854	hypothetical protein
Cluster_2708	COGs COG2378
Cluster_5280	hypothetical protein
Cluster_6256	hypothetical protein
Cluster_2799	hypothetical protein
Cluster_6435	hypothetical protein
Cluster_3700	hypothetical protein
Cluster_1093	hypothetical protein
Cluster_4232	Protein cII
Cluster_2057	hypothetical protein
Cluster_4654	Zn peptidase
Cluster_3995	Predicted HD superfamily hydrolase
Cluster_4195	hypothetical protein
Cluster_2529	Mobile element protein
Cluster_3584	Mobile element protein
Cluster_898	Mobile element protein
Cluster_1666	hypothetical protein
Cluster_148	ATPase involved in DNA repair
Cluster_5453	hypothetical protein

Cluster_6123	hypothetical protein
Cluster_2133	transposase
Cluster_2867	putative integrase
Cluster_5715	hypothetical protein
Cluster_5412	hypothetical protein
Cluster_6859	hypothetical protein
Cluster_2866	hypothetical protein
Cluster_3911	hypothetical protein
Cluster_743	Phage T7 exclusion protein
Cluster_6257	hypothetical protein
Cluster_4307	hypothetical protein
Cluster_1336	HipA protein
Cluster_7621	hypothetical protein
Cluster_816	hypothetical protein
Cluster_6452	hypothetical protein
Cluster_3218	hypothetical protein
Cluster_6016	hypothetical protein
Cluster_6160	hypothetical protein
Cluster_6821	hypothetical protein
Cluster_4078	hypothetical protein
Cluster_1787	hypothetical protein
Cluster_2477	hypothetical protein
Cluster_6748	hypothetical protein
Cluster_7129	hypothetical protein
Cluster_7469	hypothetical protein
Cluster_6384	hypothetical protein
Cluster_7361	hypothetical protein
Cluster_5844	hypothetical protein
Cluster_4306	hypothetical protein
Cluster_5812	hypothetical protein
Cluster_5098	hypothetical protein
Cluster_6736	hypothetical protein
Cluster_4562	hypothetical protein
Cluster_694	hypothetical protein
Cluster_5927	hypothetical protein
Cluster_2619	5'-nucleotidase (EC 3.1.3.5)
Cluster_3778	type II restriction enzyme (Eco47II, Sau96I)
Cluster_1108	Modification methylase Sau96I (EC 2.1.1.37)
Cluster_1463	hypothetical protein
Cluster_5718	hypothetical protein
Cluster_5165	hypothetical protein
Cluster_5893	hypothetical protein
Cluster_2535	Rep protein
Cluster_5013	hypothetical protein
Cluster_6390	hypothetical protein
Cluster_6845	hypothetical protein
Cluster_2354	rolling circle replication protein, Rep63 protein
Cluster_7245	rolling circle replication protein, Rep63 protein
Cluster_5925	hypothetical protein
Cluster_7592	hypothetical protein
Cluster_6020	Rep
Cluster_2554	mobilization protein MobA
Cluster_2724	Phage capsid protein #Fam0066
Cluster_1648	hypothetical protein
Cluster_6409	hypothetical protein
Cluster_4070	hypothetical protein
Cluster_1960	putative DNA helicase
Cluster_6504	hypothetical protein
Cluster_7421	Rep
Cluster_6468	Phage capsid protein #Fam0066

Cluster_7057	hypothetical protein
Cluster_7274	hypothetical protein
Cluster_3535	Mobile element protein
Cluster_7080	ISSod21 transposase, pseudogene # TnpA_ISSod21
Cluster_6822	Mobile element protein
Cluster_6899	hypothetical protein
Cluster_6437	hypothetical protein
Cluster_3356	hypothetical protein
Cluster_220	hypothetical protein
Cluster_3891	hypothetical protein
Cluster_1384	hypothetical protein
Cluster_4887	hypothetical protein
Cluster_3201	Mobile element protein
Cluster_7334	hypothetical protein
Cluster_2951	hypothetical protein
Cluster_7319	hypothetical protein
Cluster_7326	hypothetical protein
Cluster_3924	Mobile element protein
Cluster_2058	AfaG
Cluster_2522	OmpA/MotB domain protein
Cluster_3613	hypothetical protein
Cluster_6111	hypothetical protein
Cluster_262	Outer membrane usher protein faeD precursor
Cluster_3858	Putative fimbrial chaperone protein
Cluster_6553	hypothetical protein
Cluster_7737	hypothetical protein
Cluster_574	hypothetical protein
Cluster_4467	Opacity protein-related protein
Cluster_4613	hypothetical protein
Cluster_5014	Mobile element protein
Cluster_98	Putative insecticidal toxin complex
Cluster_258	Putative insecticidal toxin complex
Cluster_6000	hypothetical protein
Cluster_297	Putative insecticidal toxin complex
Cluster_1609	hypothetical protein
Cluster_106	Putative insecticidal toxin complex
Cluster_3882	hypothetical protein
Cluster_543	hypothetical protein
Cluster_4766	hypothetical protein
Cluster_446	hypothetical protein
Cluster_678	hypothetical protein
Cluster_46	hypothetical protein
Cluster_29	Long-chain-fatty-acid--CoA ligase (EC 6.2.1.3)
Cluster_6270	Mobile element protein
Cluster_929	hypothetical protein
Cluster_378	hypothetical protein
Cluster_293	hypothetical protein
Cluster_7090	hypothetical protein
Cluster_7118	hypothetical protein
Cluster_3335	Prophage MuSo2, transcriptional regulator, Cro/CI family
Cluster_4910	Secreted trypsin-like serine protease
Cluster_6984	Nucleoid-associated protein NdpA
Cluster_6843	FIG138517: Putative lipid carrier protein
**In SG176 only and no other genomes[a]**	
Cluster_5097	Predicted transcriptional regulator
Cluster_1235	FIG01200656: hypothetical protein
Cluster_5648	hypothetical protein
Cluster_4686	Cold shock protein CspA
Cluster_7206	hypothetical protein

Cluster_2381	Permease of the drug/metabolite transporter (DMT) superfamily
Cluster_1972	Membrane fusion component of tripartite multidrug resistance system
Cluster_4931	FIG01206295: hypothetical protein
Cluster_3783	protein of unknown function
Cluster_58	Type I restriction-modification system, restriction subunit R (EC 3.1.21.3)
Cluster_6508	Type I restriction-modification system, specificity subunit S (EC 3.1.21.3)
Cluster_3308	Type I restriction-modification system, specificity subunit S (EC 3.1.21.3)
Cluster_711	Type I restriction-modification system, DNA-methyltransferase subunit M (EC 2.1.1.72)
Cluster_232	hypothetical protein
Cluster_6225	COG3311: Predicted transcriptional regulator
Cluster_2598	hypothetical protein
Cluster_6766	hypothetical protein
Cluster_2570	Predicted transcriptional regulator
Cluster_6394	hypothetical protein
Cluster_3767	hypothetical protein
Cluster_5076	UBA/THIF-type NAD/FAD binding protein
Cluster_5356	hypothetical protein
Cluster_6318	hypothetical protein
Cluster_6659	Conjugative transfer protein TrbL
Cluster_2174	Conjugative transfer protein TrbL
Cluster_7769	Conjugative transfer protein TrbL
Cluster_3406	Conjugative transfer protein TrbJ
Cluster_2284	plasmid encoded RepA protein
Cluster_1257	FIG00642059: hypothetical protein
Cluster_2874	hypothetical protein
Cluster_2661	hypothetical protein
Cluster_1714	Integrase
Cluster_383	hypothetical protein
Cluster_4523	hypothetical protein
Cluster_4381	putative pancortin-3
Cluster_1954	hypothetical protein
Cluster_4566	hypothetical protein
Cluster_6812	hypothetical protein
Cluster_4102	hypothetical protein
Cluster_5044	putative acetyltransferase
Cluster_6371	hypothetical protein
Cluster_5974	hypothetical protein
Cluster_5204	hypothetical protein
Cluster_3585	hypothetical protein
Cluster_4993	putative cytoplasmic protein
Cluster_5538	hypothetical protein
Cluster_3203	hypothetical protein
Cluster_3570	hypothetical protein
Cluster_3693	hypothetical protein
Cluster_5437	hypothetical protein
Cluster_5410	hypothetical protein
Cluster_3824	hypothetical protein
Cluster_5385	hypothetical protein
Cluster_3723	Riboflavin synthase eubacterial/eukaryotic (EC 2.5.1.9)
Cluster_7748	hypothetical protein
Cluster_3391	hypothetical protein
Cluster_4182	hypothetical protein
Cluster_5245	Probable transcription regulator
Cluster_3467	hypothetical protein
Cluster_5429	glyoxalase family protein
Cluster_6885	hypothetical protein
Cluster_4895	hypothetical protein
Cluster_1947	putative cytoplasmic protein
Cluster_4075	hypothetical protein
Cluster_5104	hypothetical protein

Cluster_4736	hypothetical protein
Cluster_4284	hypothetical protein
Cluster_6201	hypothetical protein
Cluster_7353	hypothetical protein
Cluster_6764	SAM-dependent methyltransferase
Cluster_5212	Excinuclease ATPase subunit
Cluster_5987	hypothetical protein
Cluster_6230	hypothetical protein
Cluster_6099	FIG01202864: hypothetical protein
Cluster_6734	putative acetyltransferase
Cluster_6323	putative acetyltransferase
Cluster_6530	hypothetical protein
Cluster_6610	hypothetical protein
Cluster_3132	FIG01202005: hypothetical protein
Cluster_674	hypothetical protein
Cluster_1034	hypothetical protein
Cluster_2221	putative restriction endonuclease
Cluster_5419	hypothetical protein
Cluster_2932	Permease of the drug/metabolite transporter (DMT) superfamily
Cluster_3735	Transcriptional regulator, AraC family
Cluster_5226	putative DNA hydrolase
Cluster_2530	hypothetical protein
Cluster_2054	FIG01201088: hypothetical protein
Cluster_5843	hypothetical protein
Cluster_6301	hypothetical protein
Cluster_705	2-isopropylmalate synthase (EC 2.3.3.13)
Cluster_3407	Transcriptional regulator, AraC family
Cluster_1258	hypothetical protein
Cluster_3721	hypothetical protein
Cluster_7069	hypothetical protein
Cluster_2567	Glycosyltransferase
Cluster_3282	hypothetical protein
Cluster_5950	hypothetical protein
Cluster_7766	hypothetical protein
Cluster_7116	hypothetical protein
Cluster_4072	hypothetical protein
Cluster_4201	hypothetical protein
Cluster_1853	hypothetical protein
Cluster_7154	hypothetical protein
Cluster_5749	hypothetical protein
Cluster_3892	hypothetical protein
Cluster_6917	hypothetical protein
Cluster_5532	hypothetical protein
Cluster_4545	Uncharacterized protein COG3236
Cluster_3567	hypothetical protein
Cluster_5163	hypothetical protein
Cluster_3647	hypothetical protein
Cluster_6274	hypothetical protein
Cluster_3809	hypothetical protein
Cluster_5032	hypothetical protein
Cluster_5568	hypothetical protein
Cluster_5477	hypothetical protein
Cluster_4777	protein of unknown function UPF0157
Cluster_6542	hypothetical protein
Cluster_5914	hypothetical protein
Cluster_5425	hypothetical protein
Cluster_1100	Putative membrane protein
Cluster_5431	GCN5-related N-acetyltransferase
Cluster_2689	hypothetical protein
Cluster_1240	2-C-methyl-D-erythritol 4-phosphate cytidylyltransferase (EC 2.7.7.60)

Cluster_1439	hypothetical protein
Cluster_2695	FkbM family methyltransferase
Cluster_2392	hypothetical protein
Cluster_1937	Alanine dehydrogenase (EC 1.4.1.1)
Cluster_1591	polysaccharide biosynthesis protein
Cluster_1989	Glycosyl transferase, group 1
Cluster_2492	hypothetical protein
Cluster_2170	Glycosyltransferase (EC 2.4.1.-)
Cluster_5433	Glycosyltransferase (EC 2.4.1.-)
Cluster_2868	Glycosyltransferase (EC 2.4.1.-)
Cluster_3357	polysaccharide biosynthesis protein
Cluster_1897	hypothetical protein
Cluster_2901	Glycosyltransferase(EC:2.4.1.-)
Cluster_2125	Glycosyl transferase, group 1
Cluster_1127	hypothetical protein
Cluster_2022	capsule biosynthesis protein, putative
Cluster_3182	conserved hypothetical protein
Cluster_3168	glycosyltransferase
Cluster_7387	hypothetical protein
Cluster_7107	hypothetical protein
Cluster_492	ATP-dependent DNA helicase pcrA (EC 3.6.1.-)
Cluster_382	FIG131328: Predicted ATP-dependent endonuclease of the OLD family
Cluster_7402	hypothetical protein
Cluster_6846	Type II/IV secretion system protein TadC, associated with Flp pilus assembly
Cluster_7174	Methylglyoxal synthase (EC 4.2.3.3)
**In 22702 only and no other genomes[a]**	
Cluster_3595	hypothetical protein
Cluster_6311	Uncharacterized protein slr1025
Cluster_4328	hypothetical protein
Cluster_6467	hypothetical protein
Cluster_2498	hypothetical protein
Cluster_4322	hypothetical protein
Cluster_2656	hypothetical protein
Cluster_3649	hypothetical protein
Cluster_2403	hypothetical protein
Cluster_7178	hypothetical protein
Cluster_3454	hypothetical protein
Cluster_6654	hypothetical protein
Cluster_4502	hypothetical protein
Cluster_3181	hypothetical protein
Cluster_5402	hypothetical protein
Cluster_5603	hypothetical protein
Cluster_4177	hypothetical protein
Cluster_7641	hypothetical protein
Cluster_5051	hypothetical protein
Cluster_4561	hypothetical protein
Cluster_5166	hypothetical protein
Cluster_4709	syc0482_c
Cluster_3997	FIG01205831: hypothetical protein
Cluster_906	Recombinase
Cluster_411	hypothetical protein
Cluster_1581	hypothetical protein
Cluster_2643	mobilization protein
Cluster_6272	hypothetical protein
Cluster_661	hypothetical protein
Cluster_2605	hypothetical protein
Cluster_2231	regulatory protein CII
Cluster_2571	hypothetical protein
Cluster_7654	Phage DNA binding protein

Cluster_5189	Phage external scaffolding protein #Protein D
Cluster_888	Phage DNA replication protein
Cluster_2404	Phage minor capsid protein - DNA pilot protein
Cluster_2081	Phage major capsid protein
Cluster_6113	Phage major capsid protein
Cluster_1909	hypothetical protein
Cluster_7002	hypothetical protein
Cluster_599	Cytidine/deoxycytidylate deaminase family protein
Cluster_1465	Type I restriction-modification system, specificity subunit S (EC 3.1.21.3)
Cluster_4862	hypothetical protein
Cluster_1106	hypothetical protein
Cluster_496	hypothetical protein
Cluster_456	Phage Integrase
Cluster_5757	hypothetical protein
Cluster_1814	Lipid carrier : UDP-N-acetylgalactosaminyltransferase (EC 2.4.1.-)
Cluster_2780	Glycosyltransferase (EC 2.4.1.-)
Cluster_1892	Alpha-1,4-N-acetylgalactosamine transferase PglJ (EC 2.4.1.-)
Cluster_1498	hypothetical protein
Cluster_1761	O-antigen flippase Wzx
Cluster_2367	hypothetical protein
Cluster_1843	conserved hypothetical protein
Cluster_3239	Nitroreductase family protein
Cluster_1362	hypothetical protein
Cluster_3283	Glycosyl transferase, group 2 family protein
Cluster_3119	Glycosyltransferase (EC 2.4.1.-)
Cluster_1377	hypothetical protein
Cluster_3674	hypothetical protein
Cluster_3420	FIG01206255: hypothetical protein
Cluster_4799	hypothetical protein
Cluster_5414	hypothetical protein
Cluster_2433	hypothetical protein
Cluster_4138	FIG01204826: hypothetical protein
Cluster_6067	hypothetical protein
Cluster_6738	hypothetical protein
Cluster_595	FIG01232333: hypothetical protein
Cluster_2494	hypothetical protein
Cluster_122	Endonuclease
Cluster_990	FIG00715517: hypothetical protein
Cluster_611	RNA polymerase sigma factor RpoD
Cluster_2070	Chromosome (plasmid) partitioning protein ParB
Cluster_1671	Chromosome (plasmid) partitioning protein ParA
Cluster_4915	hypothetical protein
Cluster_1500	Error-prone, lesion bypass DNA polymerase V (UmuC)
Cluster_5522	Error-prone repair protein UmuD
Cluster_6730	Error-prone, lesion bypass DNA polymerase V (UmuC)
Cluster_2496	hypothetical protein
Cluster_6345	hypothetical protein
Cluster_1580	Type II restriction endonuclease
Cluster_722	DNA-cytosine methyltransferase (EC 2.1.1.37)
Cluster_3092	hypothetical protein
Cluster_7234	hypothetical protein
Cluster_6436	hypothetical protein
Cluster_4616	Phage DNA invertase
Cluster_4231	Mobile element protein

In J-C2-34 only and no other genomes[a]

Cluster_7237	Ferric iron ABC transporter, iron-binding protein
Cluster_5075	Predicted signal transduction protein
Cluster_3252	Mobile element protein
Cluster_7288	Mobile element protein

Cluster_2023	capsular polysaccharide biosynthesis protein
Cluster_1882	UDP-2,3-diacetamido-2,3-dideoxy-D-mannuronic acid transferase
Cluster_3140	hypothetical protein
Cluster_1389	hypothetical protein
Cluster_2460	Glycosyl transferase, group 2 family protein
Cluster_3088	Glycosyltransferase (EC 2.4.1.-)
Cluster_1168	coenzyme F420-reducing hydrogenase, beta subunit homolog
Cluster_1522	Membrane protein involved in the export of O-antigen, teichoic acid lipoteichoic acids
Cluster_1988	hypothetical protein
Cluster_2823	Glycosyltransferase
Cluster_2684	Glycosyltransferase
Cluster_2187	Glycosyltransferase (EC 2.4.1.-)
Cluster_4459	hypothetical protein
Cluster_5066	hypothetical protein
Cluster_5533	Phage protein
Cluster_2108	hypothetical protein
Cluster_4011	hypothetical protein
Cluster_2963	hypothetical protein
Cluster_7131	hypothetical protein
Cluster_6189	hypothetical protein
Cluster_3907	hypothetical protein
Cluster_5249	FIG01200735: hypothetical protein
Cluster_5343	hypothetical protein
Cluster_7247	hypothetical protein
Cluster_6344	hypothetical protein
Cluster_4451	hypothetical protein
Cluster_2944	hypothetical protein
Cluster_4692	hypothetical protein
Cluster_4907	hypothetical protein
Cluster_3732	N-methylhydantoinase A/acetone carboxylase, beta subunit
Cluster_4061	hypothetical protein
Cluster_5478	TolA protein
Cluster_6023	hypothetical protein
Cluster_4242	hypothetical protein
Cluster_2334	Phage protein D
Cluster_6618	probable phage tail protein
Cluster_5535	Phage tail protein
Cluster_596	Phage tail protein
Cluster_7546	hypothetical protein
Cluster_6109	hypothetical protein
Cluster_4820	Phage major tail tube protein
Cluster_998	Phage tail sheath monomer
Cluster_425	Phage tail fiber protein
Cluster_4174	Tail protein I
Cluster_2842	Baseplate assembly protein J
Cluster_5890	Phage baseplate assembly protein
Cluster_4113	Baseplate assembly protein V
Cluster_4734	hypothetical protein
Cluster_4263	hypothetical protein
Cluster_6718	hypothetical protein
Cluster_453	Phage protein
Cluster_857	Phage-related portal protein
Cluster_4741	FIG01217126: hypothetical protein
Cluster_573	Phage terminase large subunit GpA
Cluster_4510	hypothetical protein
Cluster_1623	Phage integrase
Cluster_6768	hypothetical protein
Cluster_5188	hypothetical protein
Cluster_1119	hypothetical protein
Cluster_5894	hypothetical protein

Cluster_1233	Putative phage protein
Cluster_4439	FIG01204320: hypothetical protein
Cluster_3631	DNA replication protein DnaC
Cluster_3610	hypothetical protein
Cluster_7351	hypothetical protein
Cluster_4607	hypothetical protein
Cluster_6586	hypothetical protein
Cluster_3982	Phage repressor protein C2
Cluster_6214	hypothetical protein
Cluster_3803	Phage protein
Cluster_6072	hypothetical protein
Cluster_1231	hypothetical protein
Cluster_1328	hypothetical protein
Cluster_1661	Probable phiRv1 integrase
Cluster_4357	hypothetical protein
Cluster_3346	hypothetical protein
Cluster_7054	hypothetical protein
Cluster_346	ATP-dependent DNA helicase pcrA (EC 3.6.1.-)
Cluster_274	FIG131328: Predicted ATP-dependent endonuclease of the OLD family
Cluster_2356	hypothetical protein
Cluster_4470	Tellurite resistance protein-related protein
Cluster_2493	hypothetical protein
Cluster_2169	hypothetical protein
Cluster_4414	hypothetical protein
Cluster_3206	hypothetical protein
Cluster_4763	hypothetical protein
Cluster_4563	hypothetical protein
Cluster_5793	hypothetical protein
Cluster_4360	hypothetical protein
Cluster_7013	Mll3428 protein
Cluster_5745	hypothetical protein
Cluster_7537	hypothetical protein
Cluster_4968	Purine nucleoside phosphorylase (EC 2.4.2.1)
Cluster_2620	hypothetical protein
Cluster_7739	hypothetical protein
Cluster_7518	hypothetical protein
Cluster_7335	hypothetical protein
Cluster_5609	hypothetical protein
Cluster_5115	hypothetical protein
Cluster_4044	hypothetical protein
Cluster_5250	GCN5-related N-acetyltransferase
Cluster_2827	hypothetical protein
Cluster_6783	hypothetical protein
Cluster_3310	hypothetical protein
Cluster_6844	hypothetical protein
Cluster_2380	hypothetical protein
Cluster_5876	hypothetical protein
Cluster_4144	hypothetical protein
Cluster_6408	hypothetical protein
Cluster_5119	hypothetical protein
Cluster_5845	hypothetical protein
Cluster_7189	hypothetical protein
Cluster_4980	Histone acetyltransferase HPA2 and related acetyltransferases
Cluster_5612	Mobile element protein
Cluster_6975	hypothetical protein
Cluster_6159	hypothetical protein
Cluster_163	hypothetical protein
Cluster_1931	Phage protein
Cluster_2829	Type II restriction enzyme BsuBI (EC 3.1.21.4)
Cluster_6428	hypothetical protein

Cluster_7005	hypothetical protein
Cluster_2444	Type II restriction enzyme BsuBI (EC 3.1.21.4)
Cluster_6372	hypothetical protein
Cluster_5794	hypothetical protein
Cluster_5161	hypothetical protein
Cluster_5428	hypothetical protein
Cluster_920	Modification methylase PstI (EC 2.1.1.72)
Cluster_5573	hypothetical protein
Cluster_3268	hypothetical protein
Cluster_4539	hypothetical protein
Cluster_4892	hypothetical protein
Cluster_6744	hypothetical protein
Cluster_5100	hypothetical protein
Cluster_4447	hypothetical protein
Cluster_6679	hypothetical protein
Cluster_1130	hypothetical protein
Cluster_6079	hypothetical protein
Cluster_4222	Repressor protein
Cluster_5871	hypothetical protein
Cluster_6652	hypothetical protein
Cluster_6411	hypothetical protein
Cluster_514	Phage replication protein
Cluster_3267	hypothetical protein
Cluster_6808	hypothetical protein
Cluster_242	Probable tail fiber protein
Cluster_6915	hypothetical protein
Cluster_1766	hypothetical protein
Cluster_3935	uncharacterized phage-encoded protein
Cluster_5695	hypothetical protein
Cluster_6184	hypothetical protein
Cluster_5435	hypothetical protein
Cluster_3598	hypothetical protein
Cluster_985	hypothetical protein
Cluster_6445	hypothetical protein

[a]LS-BSR gene clusters that are highly conserved (LS-BSR value ≥0.8) in one genome, and absent (LS-BSR value <0.4) in all other genomes analyzed.

Supplemental Table 4. Predicted protein functions of the LS-BSR gene clusters that were highly-conserved among the *V. parahaemolyticus* O3:K6 isolate genomes analyzed

LS-BSR Cluster ID	Predicted Protein Function
**Post-1995 O3:K6 Isolate Genomes (including AF91)[a]**	
Cluster_1800	hypothetical protein
Cluster_6903	putative transcriptional regulator
Cluster_3170	putative transmembrane protein
Cluster_1118	putative Y4mE
Cluster_5903	hypothetical protein
Cluster_5427	hypothetical protein
Cluster_6606	hypothetical protein
Cluster_5790	conserved hypothetical protein
Cluster_5101	hypothetical protein
Cluster_5632	hypothetical protein
Cluster_6980	hypothetical protein
Cluster_7375	hypothetical protein
Cluster_6388	conserved hypothetical protein
Cluster_7251	hypothetical protein
Cluster_7295	putative integrase
Cluster_7373	hypothetical protein
Cluster_7761	hypothetical protein
**Post-1995 O3:K6 Isolate Genomes (not including AF91)[a]**	
Cluster_1205	putative integrase
Cluster_6130	hypothetical protein
Cluster_2366	putative inner membrane protein
Cluster_610	putative HsdS polypeptide, part of CfrA family
Cluster_960	type I restriction enzyme M protein
Cluster_5563	hypothetical protein
Cluster_7047	hypothetical protein
Cluster_5754	hypothetical protein
Cluster_204	type I restriction enzyme R protein
Cluster_815	hypothetical protein
Cluster_3644	hypothetical protein
Cluster_1861	hypothetical protein
Cluster_594	putative helicase
Cluster_4198	hypothetical protein
Cluster_4773	hypothetical protein
Cluster_976	bacteriophage f237 ORF8
Cluster_5889	hypothetical protein
Cluster_2076	hypothetical protein
Cluster_6793	hypothetical protein
Cluster_4127	hypothetical protein
Cluster_3933	hypothetical protein
Cluster_5562	hypothetical protein
Cluster_3934	hypothetical protein
Cluster_5381	putative acetyltransferase
Cluster_4129	hypothetical protein
Cluster_3157	putative protein Ymh
Cluster_5519	hypothetical protein
Cluster_4640	hypothetical protein
Cluster_6799	hypothetical protein
Cluster_7053	hypothetical protein
Cluster_4149	putative threonine efflux protein
Cluster_1283	hypothetical protein
Cluster_6675	hypothetical protein
Cluster_1598	hypothetical protein
Cluster_327	putative phage-related protein
Cluster_7161	putative phage-related protein
Cluster_1944	putative pore-forming cytotoxin integrase
Cluster_932	hypothetical protein
Cluster_2304	hypothetical protein
Cluster_4740	hypothetical protein
Cluster_196	hypothetical protein
Cluster_545	hypothetical protein

Cluster_957	hypothetical protein
Cluster_1610	hypothetical protein
Cluster_1871	hypothetical protein
Cluster_1237	putative phage protein
Cluster_245	hypothetical protein
Cluster_156	hypothetical protein
Cluster_799	hypothetical protein
Cluster_1391	hypothetical protein
Cluster_654	putative site-specific recombinase
Cluster_4145	hypothetical protein
Cluster_2138	hypothetical protein
Cluster_2273	putative IS1328 transposase
Cluster_272	hypothetical protein
Cluster_798	hypothetical protein
Cluster_4095	hypothetical protein
Cluster_1109	putative ATP-binding protein
Cluster_6650	putative ATP-binding protein
Cluster_60	hypothetical protein
Cluster_179	hypothetical protein
Cluster_6246	putative colicin polypeptide
Cluster_7732	putative ATP-dependent exoDNAse (exonuclease V), alpha subunit
Cluster_7733	putative ATP-dependent exoDNAse (exonuclease V), alpha subunit
Cluster_3915	hypothetical protein
Cluster_147	hypothetical protein
Cluster_1156	hypothetical protein
Cluster_6879	hypothetical protein
Cluster_7158	hypothetical protein
Cluster_6103	hypothetical protein
Cluster_1818	hypothetical protein
Cluster_7390	hypothetical protein
Cluster_7502	conserved hypothetical protein
Cluster_7448	hypothetical protein
Cluster_6689	hypothetical protein
Cluster_7521	hypothetical protein
Cluster_6004	conserved hypothetical protein
Cluster_6148	bacteriocin immunity protein
**Pre-1995 O3:K6 Isolate Genomes**[a]	
Cluster_1046	Rhamnulokinase (EC 2.7.1.5)
Cluster_1065	6-phospho-beta-glucosidase (EC 3.2.1.86)
Cluster_1359	PTS system, cellobiose-specific IIC component (EC 2.7.1.69)
Cluster_1442	hypothetical protein
Cluster_1486	L-rhamnose isomerase (EC 5.3.1.14)
Cluster_151	Alfa-L-rhamnosidase (EC 3.2.1.40)
Cluster_1768	NADH-dependent butanol dehydrogenase A (EC 1.1.1.-)
Cluster_1842	Alcohol dehydrogenase (EC 1.1.1.1)
Cluster_2483	Transcriptional regulator of rhamnose utilization, AraC family
Cluster_2595	FIG01201305: hypothetical protein
Cluster_2969	oxidoreductase, aldo/keto reductase family
Cluster_3117	L-rhamnose operon regulatory protein RhaS
Cluster_3166	Mobile element protein
Cluster_3281	Rhamnulose-1-phosphate aldolase (EC 4.1.2.19)
Cluster_5613	hypothetical protein
Cluster_5798	hypothetical protein
Cluster_5835	COG4925: Uncharacterized conserved protein
Cluster_6039	PTS system, cellobiose-specific IIA component (EC 2.7.1.69)
Cluster_6045	PTS system, cellobiose-specific IIB component (EC 2.7.1.69)
Cluster_6186	Mobile element protein
Cluster_6218	hypothetical protein
Cluster_7084	Mobile element protein
Cluster_7159	hypothetical protein
Cluster_7207	hypothetical protein
Cluster_7464	Glucose 1-dehydrogenase (EC 1.1.1.47)
Cluster_870	hypothetical protein

[a]LS-BSR gene clusters that were highly-conserved (LS-BSR value ≥ 0.8) and divergent (LS-BSR value <0.8 and ≥0.4) or absent (LS-BSR value <0.4) from all other genomes analyzed.

Supplemental Table 5. Primers used in this study

Gene	Predicted Protein Function	Primer ID	Amplicon (bp)	Sequence 5'-3'	Source
tl	thermolabile hemolysin	tlF	450	AAAGCGGATTATGCAGAAGCACTG	Bej *et al*. (1999) J. Microbiol. Methods
		tlR		GCTACTTTCTAGCATTTTCTCTGC	"
tdh	thermostabile direct hemolysin	tdhF	269	GTAAAGGTCTCTGACTTTTGGAC	"
		tdhR		TGGAATAGAACCTTCATCTTCACC	"
trh	thermostabile direct-related hemolysin	trhF	359	CTGAATCMCCAGTTAASGC	Meador *et al.* (2007) J. Clin. Microbiol.
		trhR		ATGYCCATTKCCGCTCTC	"
ORF8	hypothetical protein	O3MM824	369	AGGACGCAGTTACGCTTGATG	Myers *et al*. (2003) Appl. Environ. Microbiol.
		O3MM1192		CTAACGCATTGTCCCTTTGTAG	"
vp1670 (T3SS1)	YscP-like translocation protein	vp1670F	338	GCGTCACTGCATTCGACAGG	Meador *et al.* (2007) J. Clin. Microbiol.
		vp1670R		GACTCGTGTGACTCTGCTG	"
vp1680 (T3SS1)	type III secreted effector	vp1680F	999	CAGGCATCAGCCAATCCCAATCTTC	This study
		vp1680R		TCAGTCCGTCAGGCTACCGAG	"
vp1686 (T3SS1)	type III secreted effector	vp1686F	991	GCCATCGGTAAAGCCGTGATAACC	This study
		vp1686R		TGAAGGCAAACTCAGCATTGGTGG	"
vpa1346 (T3SS2α)	type III secreted effector	YopPF	751	CGTCCAACTCTATTGTTGTGATATGGCG	Vora *et al.* (2005) Proc. Natl. Acad. Sci. USA
		vpa1346R		GGGCTCTGATCTTCGTGAACAG	This study
vpa1354 (T3SS2α)	EscU-like export protein	vpa1354F	522	CTGGCGAGCCTTCCGTCTC	Meador *et al.* (2007) J. Clin. Microbiol.
		vpa1354R		CTGGTGAATGGTTCTCCGCAG	"
vpa1362 (T3SS2α)	type III secreted effector	vpa1362F	950	GAGCAACCGAACGCATCGCT	This study
		vpa1362R		CGCAGAGAACCAAGAATCTCTGTGG	"
vopP (*vpa1346* of T3SS2β)	type III secreted effector	vopPF	791	GCAGGACCGGTTGAGAGTAATC	This study
		vopPR		GTAATCTGCTTATCTTCTGCCTC	"
vscU2 (*vpa1354* of T3SS2β)	EscU-like export protein	vscU2F	1,006	GGAGTCAACAGAACCTAAGAAATACCC	This study
		vscU2R		GGTTGATATAATCACGGCGACATCTC	"
vopD2 (*vpa1362* of T3SS2β)	type III secreted effector	vopD2F	666	GCAGCCGCTTCTGCAAACGTATC	This study
		vopD2R		CTCATTGAGTTTGACCGCTTCTGTGG	"
rstA	phage replication initiation protein	rstAM13F	906	TGTAAAACGACGGCCAGTCGATTGCACAAGCATGAGGA	This study
		rstAM13R		CAGGAAACAGCTATGACCGGATTTTCCCGTACTGACGA	"

[a]The M13 sequence is underlined.

References

Bej, A.K., Patterson, D.P., Brasher, C.W., Vickery, M.C., Jones, D.D., and Kaysner, C.A. (1999) Detection of total and hemolysin-producing *Vibrio parahaemolyticus* in shellfish using multiplex PCR amplification of *tl, tdh,* and *trh. J Microbiol Methods* **36**: 215-225.

Gertz, E.M., Yu, Y.K., Agarwala, R., Schaffer, A.A., and Altschul, S.F. (2006) Composition-based statistics and translated nucleotide searches: improving the TBLASTN module of BLAST. *BMC Biol* **4**: 41.

Hazen, T.H., Wu, D., Eisen, J.A., and Sobecky, P.A. (2007) Sequence characterization and comparative analysis of three plasmids isolated from environmental *Vibrio* spp. *Appl Environ Microbiol* **73**: 7703-7710.

Krzywinski, M., Schein, J., Birol, I., Connors, J., Gascoyne, R., Horsman, D. et al. (2009) Circos: an information aesthetic for comparative genomics. *Genome research* **19**: 1639-1645.

Lobocka, M.B., Rose, D.J., Plunkett, G., 3rd, Rusin, M., Samojedny, A., Lehnherr, H. et al. (2004) Genome of bacteriophage P1. *J Bacteriol* **186**: 7032-7068.

Meador, C.E., Parsons, M.B., Bopp, C.A., Gerner-Smidt, P., Painter, J.A., and Vora, G.J. (2007) Virulence gene- and pandemic group-specific marker profiling of clinical *Vibrio parahaemolyticus* isolates. *J Clin Microbiol* **45**: 1133-1139.

Myers, M.L., Panicker, G., and Bej, A.K. (2003) PCR detection of a newly emerged pandemic *Vibrio parahaemolyticus* O3:K6 pathogen in pure cultures and seeded waters from the Gulf of Mexico. *Appl Environ Microbiol* **69**: 2194-2200.

Nasu, H., Iida, T., Sugahara, T., Yamaichi, Y., Park, K.S., Yokoyama, K. et al. (2000) A filamentous phage associated with recent pandemic *Vibrio parahaemolyticus* O3:K6 strains. *J Clin Microbiol* **38**: 2156-2161.

Rasko, D.A., Myers, G.S., and Ravel, J. (2005) Visualization of comparative genomic analyses by BLAST score ratio. *BMC Bioinformatics* **6**: 2.

Stamatakis, A. (2006) RAxML-VI-HPC: maximum likelihood-based phylogenetic analyses with thousands of taxa and mixed models. *Bioinformatics* **22**: 2688-2690.

Tamura, K., Peterson, D., Peterson, N., Stecher, G., Nei, M., and Kumar, S. (2011) MEGA5: molecular evolutionary genetics analysis using maximum likelihood, evolutionary distance, and maximum parsimony methods. *Mol Biol Evol* **28**: 2731-2739.

Vora, G.J., Meador, C.E., Bird, M.M., Bopp, C.A., Andreadis, J.D., and Stenger, D.A. (2005) Microarray-based detection of genetic heterogeneity, antimicrobial resistance, and the viable but nonculturable state in human pathogenic *Vibrio* spp. *Proc Natl Acad Sci USA* **102**: 19109-19114.
